# Pluripotent Stem Cell‐Derived Hematopoietic Progenitors Are Unable to Downregulate Key Epithelial‐Mesenchymal Transition‐Associated miRNAs

**DOI:** 10.1002/stem.2724

**Published:** 2017-10-27

**Authors:** Ellie Meader, Tomas Barta, Dario Melguizo‐Sanchis, Katarzyna Tilgner, David Montaner, Ashraf A. El‐Harouni, Lyle Armstrong, Majlinda Lako

**Affiliations:** ^1^ Institute of Genetic Medicine, Newcastle University Newcastle upon Tyne United Kingdom; ^2^ Department of Histology and Embryology Faculty of Medicine, Masaryk University Brno Czech Republic; ^3^ Centro de Investigación Príncipe Felipe Valencia Spain; ^4^ Princess Al Jawhara Al‐Brahim Center of Excellence in Research of Hereditary Disorders, King Abdulaziz University Jeddah Saudi Arabia

**Keywords:** Human embryonic stem cells, miRNAs, Epithelial‐mesenchymal transition, Hematopoietic differentiation

## Abstract

Hematopoietic stem cells derived from pluripotent stem cells could be used as an alternative to bone marrow transplants. Deriving these has been a long‐term goal for researchers. However, the success of these efforts has been limited with the cells produced able to engraft in the bone marrow of recipient animals only in very low numbers. There is evidence that defects in the migratory and homing capacity of the cells are due to mis‐regulation of miRNA expression and are responsible for their failure to engraft. We compared the miRNA expression profile of hematopoietic progenitors derived from pluripotent stem cells to those derived from bone marrow and found that numerous miRNAs are too highly expressed in hematopoietic progenitors derived from pluripotent stem cells, and that most of these are inhibitors of epithelial‐mesenchymal transition or metastasis (including miR‐200b, miR‐200c, miR‐205, miR‐148a, and miR‐424). We hypothesize that the high expression of these factors, which promote an adherent phenotype, may be causing the defect in hematopoietic differentiation. However, inhibiting these miRNAs, individually or in multiplex, was insufficient to improve hematopoietic differentiation in vitro, suggesting that other miRNAs and/or genes may be involved in this process. Stem Cells *2018;36:55–64*

**Significance Statement**

Hematopoietic stem cell (HSC) transplants are an effective treatment for leukemia; however, there is a shortage of appropriate donors. HSCs made from pluripotent stem cells could provide a valuable alternative, but deriving these cells has proved difficult. This study shows that high expression of a network of microRNAs may be a factor preventing this cell type from arising in vitro. Although inhibiting them was insufficient to improve in vitro function due to redundancy between microRNAs, this work provides insight into the mechanisms of differentiation which may prove useful in future experiments.

## Introduction

Hematopoietic stem cell (HSC) transplants are an effective treatment for leukemia. The patient is transplanted with HSCs from a healthy donor, which then migrate‐to and engraft‐in the bone marrow niche. Once there, they resume their normal function: producing replacement blood cells of all lineages throughout the patient's lifetime. The transplanted cells must match the patient's human leukocyte antigen type or risk causing potentially deadly graft‐versus‐host disease and unfortunately there is a shortage of matching donors.

Generating HSCs from pluripotent stem cells has been proposed as a potential solution. However, attempts to do this in vitro using human cells have not yielded clinically viable results: in spite of recent advances (reviewed by Slukvin 1), the pluripotent stem cell‐derived hematopoietic progenitors (P‐HPCs) do not engraft in sufficient numbers in the bone marrow niche when transplanted. The few cells that do engraft fail to reconstitute an entire hematopoietic system; they produce mostly myeloid lineage progeny, and survive only in the short or medium term 2, 3, 4, 5, 6, whereas HSCs derived from donor marrow can reconstitute an entire hematopoietic system over serial transplants.

It is now possible to generate HSCs capable of engrafting and reconstituting the hematopoietic system at low frequency by ectopically expressing certain hematopoietic transcription factors 7, 8. However, this technique is too risky to be used clinically due to the oncogenic potential of these factors 9, 10.

Risueño et al. achieved higher engraftment by injecting P‐HPCs directly into the bone marrow, rather than introducing them to the bloodstream 2. However, this is limited to the femur into which they were injected and they did not colonize the contra‐lateral femur or the other hematopoietic organs. The means that cells can engraft when they are introduced directly into the niche but are unable to migrate to other hematopoietic niches, unlike donor‐derived HSCs. This suggests that they are unable to gain a motile phenotype in response to chemotactic signals.

In vitro systems for hematopoietic differentiation have attempted to mimic embryonic development, either by exposing the cells to a combination of hematopoietic cytokines or by coculturing them with cells from the in vivo niche for hematopoietic development 11. In the embryo, mesoderm gives rise to hemogenic endothelium which in turn produces hematopoietic cells 12. The process by which the hemogenic endothelial cells round‐up and detach from the surrounding endothelium is known as endothelial‐hematopoietic transition (EHT), so‐called because of its similarity to epithelial‐mesenchymal transition (EMT) 13, 14. Once free of the endothelium the cells migrate to the fetal liver, and eventually to their adult niche in the bone marrow 15. Hematopoietic cells of various types arise from the hemogenic endothelium throughout the vascular system during embryonic development, but HSCs with self‐renewal potential arise in a short time‐period and only from the floor of the fetal aorta 16. The EHT process has recently received a lot of attention from groups attempting to make HSC from pluripotent stem cells, because all blood cells arise via EHT both in vivo and in vitro 1.

Inappropriate miRNA expression was suggested as a potential explanation for the failure of P‐HPCs to engraft by Risueño et al. when they compared miRNA expression in HSCs to that in P‐HPCs and found that P‐HPCs over‐express a number of them. miRNAs inhibit expression of their target genes by binding to specific sequences on the untranslated region of the mRNA. A miRNA can modulate the expression of multiple genes and they are therefore key regulators of development.

Incorrect expression levels of miRNAs could have drastic effects on the function of P‐HPCs; hence we studied miRNA expression in P‐HPCs in comparison to HSCs to identify candidate miRNAs, whose expression could be manipulated to result in successful P‐HPC derivation. In this article, we present a comprehensive profile of miRNA expression in P‐HPCs. We identify key miRNA candidates whose expression is mis‐regulated during human embryonic stem cell/human induced pluripotent stem cell (hESC/hiPSC) differentiation, although our data suggest that miRNA inhibition on its own is insufficient to improve hematopoietic differentiation in vitro, highlighting the complexity of this process.

## Methods

### Tissue Culture

hESC line H9 (WiCell, Madison, WI, USA) and hiPSC line SB‐Ad3 were cultured in STEMPRO medium (Thermo Fisher Scientific, Cramlington, UK) on vitronectin (Stem cell technologies, Cambridge, UK) coated 6‐well plates. The cells were mechanically passaged at a ratio of 1:3 every 4 to 5 days or when they reached 75% confluence.

### Hematopoietic Differentiation

Experiments were done using the protocol described by Olivier et al. 17, modified by using only the first 12 days of the protocol, that is, stopping before the cytokines for erythroid specific differentiation are added.

### Microarray Analysis

Two replicates of undifferentiated H9 hESC line and CD31 + CD34 + KDR + CD45‐ subpopulation from day 4 of differentiation were subjected to RNA extraction and hybridization to an Agilent *G4470C‐021827* array using the Agilent protocol “miRNA Microarray System with miRNA Complete Labeling and Hyb Kit,” Version 2.1. Human placenta cells were used as an internal control. This data was compared with the expression profile of human CD34+ bone marrow cells obtained from Gene Expression Omnibus, accession number *GSM595699*. Expression data were normalized using quantile normalization 18 and differential miRNA expression was estimated using Limma package 19 from Bioconductor. Statistical significance (adjusted *p* values) was used to select the miRNAs that were differentially expressed. Analysis of all the mis‐regulated miRNAs was carried out using information from various databases including Targetscan 20 and miRbase 21 as well as literature searches on NCBI.

### Quantitative Reverse Transcription Polymerase Chain Reaction (qRT‐PCR)

RNA was extracted using the Reliaprep RNA cell miniprep system (Promega, Madison, WI, USA). For qRT‐PCR analysis of miRNA, reverse transcription was done using the TaqMan Micro‐RNA RT Kit with a specific TaqMan Micro‐RNA primer. TaqMan Universal PCR Mastermix II and TaqMan MicroRNA Assays were used for the qRT‐PCR (all from Thermo Fisher Scientific) RNU44 and RNU48 were chosen as internal controls. CD34+ human adult bone marrow and CD34+ cord blood cells (AllCells, Alameda, CA, USA) were used as positive controls for qPCR analysis.

For the analysis of gene expression by qRT‐PCR, 1 μg of RNA was used in a 20 μl GoScript (Promega) reverse transcription reaction, according to the manufacturer's instructions. qRT‐PCR was then performed using the SYBR Green qRT‐PCR kit (Life Technologies, Cramlington, UK). Glyceraldehyde 3‐phosphate dehydrogenase (GAPDH) was used as the internal control. All qRT‐PCR reactions were done at 10 µl in triplicate on a 386 well plate in a TaqMan 7900 or a QuantStudio 7 (Thermo Fisher Scientific) machine. Primers were designed using the NCBI Primer‐BLAST tool and analyzed for primer dimers and secondary structure formation using Thermo Fisher's multiple primer analyzer software. Primer sequences are in Supporting Information Table 3. Significance was determined by one‐way ANOVA with three replicates.

### Flow Cytometry and Cell Sorting

Cells were sorted on a FACSARIA (BD Biosciences, San Jose, CA, USA) with markers CD34, CD45, CD41a, CD235a, (BD Biosciences), and CD43 (Life Technologies). Cells were analyzed after miRNA inhibition on a LSRII (BD Biosciences) flow cytometry machine using the same markers. Results were analyzed using BD FACSDIVA software. At least 10,000 events were acquired for each experiment.

### Colony Forming Unit Assays

Six thousand unsorted cells were taken from differentiation culture and transferred to a 3‐ml aliquot of MethoCult media and plated into two 10‐mm cell culture dishes scored with 2‐mm grids. These were then incubated for 14 days. Colonies in each dish were counted and scored based on their morphology. Inhibitions were compared with control data using univariate one‐way analysis of covariance with effect of passage number on numbers of hematopoietic progenitors as the covariate (see Supporting Information Figs. 1, 2). *p* values are calculated as Sidak corrected bootstrapped significance. Analysis was performed using SPSS statistics software. Figures show estimated marginal means.

### Lipofection

Lipofectamine‐RNAi complexes were prepared by diluting 20 pmol of mirVana miRNA inhibitor (Life technologies) or control (mirVana miRNA inhibitor negative control, Life technologies, or Flourescein conjugate control, Santa Cruz biotechnology) in 50 μl Opti‐MEM I Medium (Life technologies) and 6 μl of Lipofectamine RNAiMax (Life technologies) in 50ul Opti‐MEM I Medium. Both were incubated at room temperature for 5 minutes, then mixed and incubated at room temperature for a further 20 minutes. For Multiplex reactions, 20 pmol of each inhibitor was used, while increasing the quantity of lipofectamine to maintain the miRNA‐inhibitor to lipofectamine ratio. The complexes were added to one well of a 12 well plate with 1 ml of differentiation medium at day 10 of differentiation. Cells were analyzed after 48 hours.

### Network Analysis

A list of all experimentally validated target genes of the selected miRNAs was downloaded from http://mirtarbase.mbc.nctu.edu.tw/ 22, a list of genes associated with EMT was downloaded from http://dbemt.bioinfo-minzhao.org/ 23. Cytoscape v3.5.1 24 was used to generate and visualize the genes which are both regulated by the chosen miRNAs and which are validated regulators of EMT.

## Results

### Hematopoietic Progenitors Derived from Pluripotent Cells Over‐Express EMT Suppressing miRNAs

**Figure 1:**
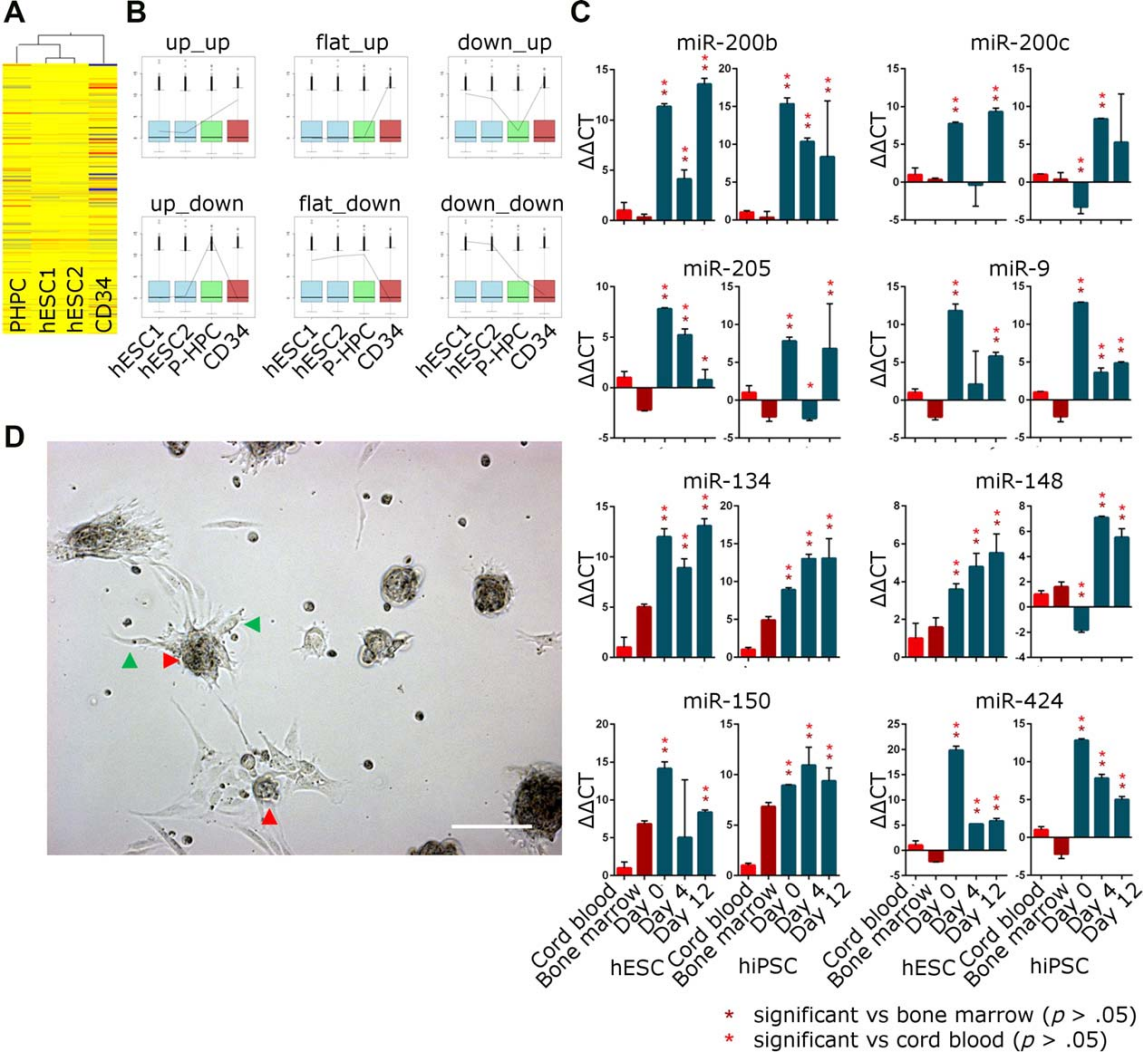
miRNA expression in pluripotent stem cells and bone marrow. **(A):** Microarray data showing the relationship between miRNA expression in day 4 differentiated CD34 + CD31 + KDR + CD45‐ (P‐HPC), two replicates of undifferentiated hESC (hESC1 and hESC2) and CD34+ cells derived from human bone marrow. **(B):** Schematic graphs showing representative examples of estimated differential gene expression from microarray data in different cell types outlined in 1(A). The distribution of normalized intensity values for each sample is displayed in the box plots, and the relative fluorescence of the individual miRNA is shown as a line graph. miRNAs have been sorted according to the shape of the graph, for example, flat‐down, flat‐up, and so forth. For example, the flat down group miRNAs are expressed at similar levels in the hESC lines and in the day 4 differentiated hESC cells but at a lower level in the CD34+ bone marrow cells. **(C):** qRT‐PCR validation of miRNA data in a hESC and an hiPSC cell line (H9 and SB‐AD3, respectively), compared with CD34+ cells derived from human cord blood and bone marrow. Day 0 = unsorted undifferentiated cells; day 4 = CD31+ CD34+ KDR+ CD45‐ (hemogenic endothelium); day 12 = CD34low/‐CD41a‐CD43 + CD235a‐CD45+ (committed hematopoietic progenitors). Data are presented as mean ± SEM, *n* = 3. **(D):** Representative micrograph of day 6 hESC‐derived hematopoietic colonies. The bar represents 100 µm. Cells with endothelial morphology can be seen around the edges of the colonies (green arrows) and some cells are beginning to round up and “bud off” the colonies (red arrows), similar to in vivo hematopoietic cluster formation. Abbreviations: hESC, human embryonic stem cells; hiPSC, human induced pluripotent stem cell; P‐HPCs, pluripotent stem cell‐derived hematopoietic progenitor's cells.

A microarray approach was used to compare miRNA expression between undifferentiated pluripotent stem cells, CD31 + CD34 + KDR + CD45‐P‐HPCs derived from pluripotent stem cells and CD34+ bone marrow cells (Fig. 1A). The differentially expressed miRNAs were grouped based on the pattern of their expression (Fig. 1B, Supporting Information Table 1). The flat‐down group, that is, the group of miRNAs which were expressed at high levels in both undifferentiated pluripotent stem cells and in P‐HPCs, but at low levels in the bone marrow hematopoietic progenitors contained 36 miRNAs. Of these miRNAs the majority are described in the literature as having functions as tumor or EMT suppressors (see Supporting Information Table 2). Several of these miRNAs (miR‐148a, miR‐200c and miR‐200b, the miR‐205, miR‐34a, miR‐424, miR‐9, miR‐18b, and miR‐34a) were chosen for validation by qRT‐PCR in differentiated cells derived from one hESC and one hiPSC line. A more advanced differentiation protocol allowed analysis of their expression in both early stage hemogenic endothelial cells (day 4: CD31 + CD34 + KDR + CD45‐), and at later stages of differentiation when committed hematopoietic progenitors have undergone EHT (day 12: CD34low/–CD43 + CD45+). The qRT‐PCR results confirm that these miRNAs maintain their high expression throughout differentiation from pluripotent stem cells to hematopoietic progenitors, compared with bone marrow and cord blood CD34+ cells, which have low expression (Fig. 1C).

The continued high expression of these EMT‐suppressing miRNAs indicates that the miRNA regulation in the differentiation process fails to recapitulate that of embryonic hematopoietic differentiation. It is unsurprising that pluripotent stem cells would have high levels of EMT suppressors as they are an epithelial cell type, and bone marrow CD34+ cells have low levels of these miRNAs as they are of a migratory nature. That P‐HPCs express high levels of EMT suppressors suggests that despite appearing to “bud” from the hemogenic endothelium as normal blood cells do (see Fig. 1D), they have not fully undergone the transformation from an adherent endothelial phenotype to a motile hematopoietic phenotype. Failure to upregulate motility related genes as well as EMT suppressors’ tendency to inhibit self‐renewal could explain the issues that have been reported in transplant experiments using this cell type. By inhibiting these EMT suppressing miRNAs, we hoped to improve the ability of these hematopoietic cells to home to the niche.

### Lipofection Is an Effective Short‐Term Method of Inhibiting miRNAs in P‐HPCs

**Figure 2:**
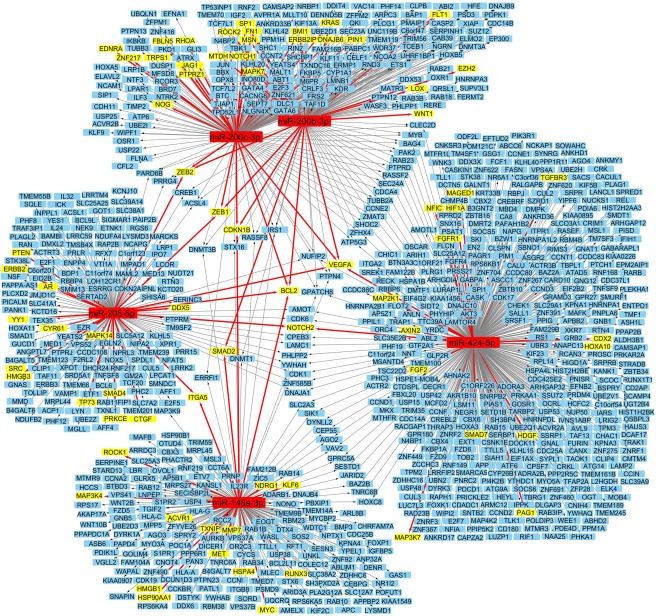
Highly expressed miRNAs and their involvement in endothelial‐hematopoietic transition. A graphical representation of the genes targeted by the five selected miRNAs. All experimentally validated targets of these miRNAs are shown, with arrows pointing from the miRNAs to the genes which they regulate. Genes known to be involved in epithelial‐mesenchymal transition are highlighted in yellow, red arrows point to these genes.

Five of the miRNAs which were demonstrated to maintain high expression in P‐HPCs but not HSCs were chosen for inhibition, based on their well‐defined and validated roles in the EMT process: miR‐148a, miR‐200c, and miR‐200b, the miR‐205, miR‐424. Network analysis shows that between them, these miRNAs regulate 72 out of 344 genes involved in EMT. Eight of these genes are targets of more than one of these miRNAs, including key EMT transcription factors *ZEB1* and *ZEB2* (Fig. 2)

We inhibited the miRNAs by introducing small complimentary RNA molecules into the differentiating cells via lipofection. Although lipofection of inhibitors is a short‐term method of inhibiting miRNAs, it has the advantage of having no effect on the genome, limiting the risk of oncogenic transformation.

**Figure 3:**
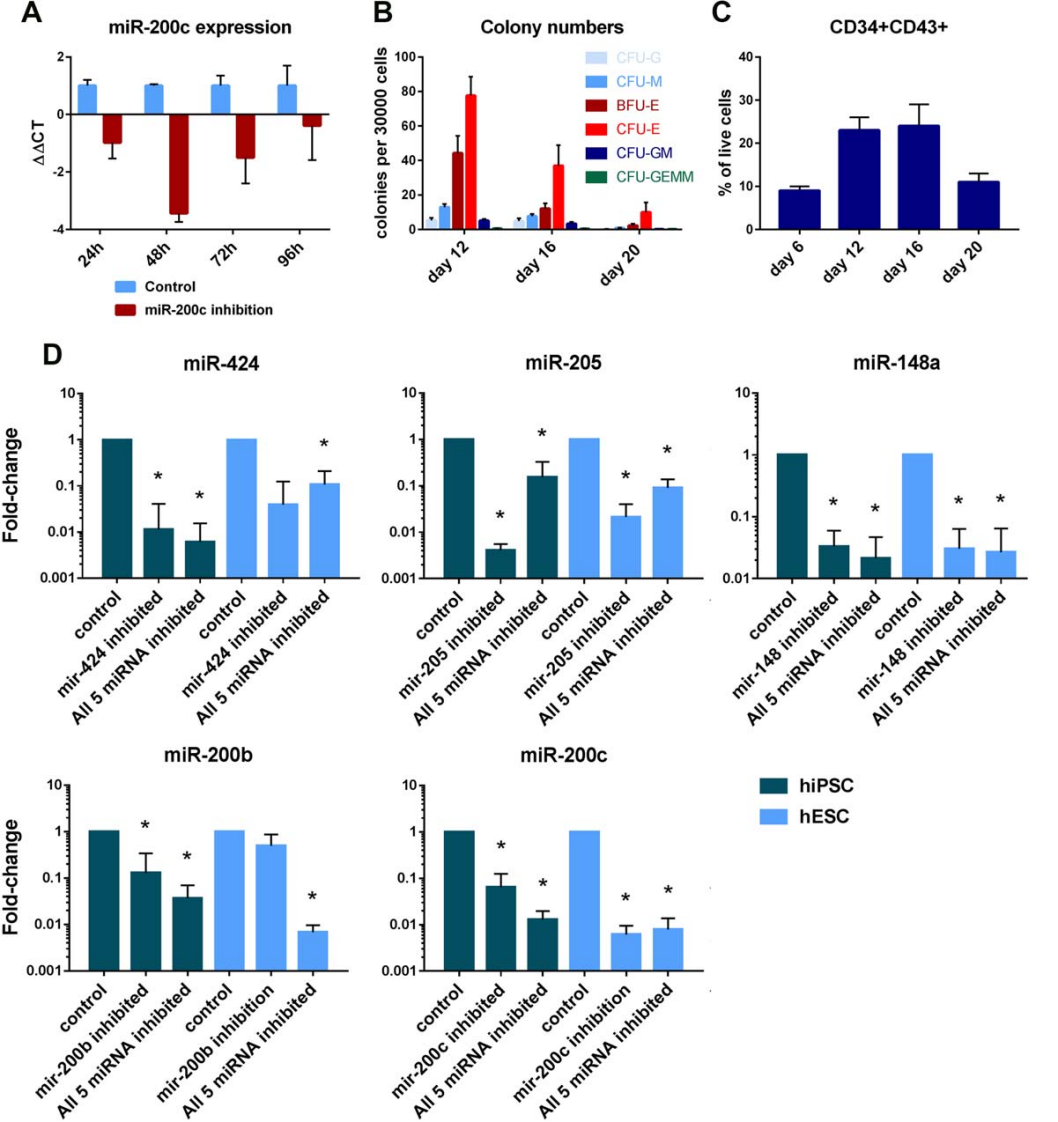
Inhibition of miRNA expression in pluripotent stem cell‐derived hematopoietic progenitor's cells. **(A):** qRT‐PCR expression analysis of mir‐200c in hESCs at 24, 48, 72, and 96 hours after lipofection. Data are shown as mean ± SEM, *n* = 3. **(B):** Schematic graph showing numbers of hematopoietic colonies produced from an unsorted sample of hiPSC cells at day 12, day 16, and day 20 of differentiation. Data are shown as mean ± SEM, *n* = 3. Representative micrographs of typical hematopoietic colonies are shown in Supporting Information Figure 1. **(C):** Flow cytometry data showing the percentage of CD34 + CD43+ hematopoietic progenitors produced from hiPSCs at day 6, day 12, day 16, and day 20. Data are shown as mean ± SEM, *n* = 3. **(D):** qRT‐PCR data showing fold change in miRNA expression after inhibition of either a single miRNA or a combination of miR‐424, miR‐205, miR‐148a, miR‐200b, and miR‐200c in the hiPSC and the hESC lines. Fold change was calculated using the ΔΔCT method. miRNAs were inhibited at day 10 of inhibition, and analyzed at day 12. Data are presented as fold‐change derived from mean 2^–ΔΔCT^ values ±SEM, *n* = 3, *, *p* < .05. Abbreviations: BFU‐E, burst forming unit‐erythroid; CFU‐E, colony forming unit erythroid; CFU‐G, colony forming unit granulocyte; CFU‐M, colony forming unit macrophage; CFU‐GM, colony forming unit granulocyte‐macrphage; CFU‐GEMM, colony forming unit granulocyte‐erythrocyte‐monocyte‐macrphage; hESC, human embryonic stem cells; hiPSC, human induced pluripotent stem cell.

miRNA inhibition was timed to coincide with the EHT of definitive progenitors as this is when we predict EMT‐suppressing miRNAs will have the greatest negative effect on hematopoiesis. Pilot experiments showed that miRNA inhibition is at peak effectiveness up to 48 hours after inhibition (see Fig. 3A). CD43 + CD34+ cells begin to appear at day 6 but numbers peak between day 12 and day 16 and hematopoietic colony forming units peak at day 12 (Fig. 3B, 3C). Therefore, miRNAs were inhibited at day 10, and the cells analyzed at day 12. The inhibitions significantly reduced the amount of their target miRNAs both individually and in combination with other inhibitors (Fig. 3D).

### Inhibition of Highly‐Expressed miRNAs Is Insufficient to Improve Hematopoietic Function in Hematopoietic Progenitors Derived from Pluripotent Cells

To test the effect of inhibition on hematopoietic differentiation from pluripotent cells, we analyzed hematopoietic marker expression, colony forming potential, and expression of key miRNA target genes in day 12 differentiated cells. Hematopoietic differentiation is controlled by numerous transcription factors which are engaged in a stepwise manner but since we are focusing on the EHT process we studied those transcription factors specifically involved in that step. *RUNX1* is essential for EHT and *GATA2* and *SCL* are key upstream regulators of this process 16, 25. *c‐MYB* is essential for definitive hematopoiesis as well as being a direct target of miR‐200c 26, 27 and RORA has been shown as a transcription factor able to promote EHT in hematopoietic cells derived in vitro. ZEB1 and ZEB2 are essential regulators of EMT 28 as well as direct targets of several of the inhibited miRNAs. If the miRNA inhibition was promoting EHT one would expect to see increases in the expression of these genes.

Inhibition was effective for all the miRNAs, both individually and in combination. miR‐205 inhibition alone inhibits miR‐205 transcript levels more than miR‐205 in combination with the other inhibitors, although miR‐205 is still inhibited to a significant degree in the combinatorial experiments. This is an interesting result for which we do not have a complete explanation but we do know that “crosstalk” occurs between miRNA transcripts and that these miRNAs are involved in complex feedback loops 29 with the genes that they regulate (see Fig. 2).

**Figure 4:**
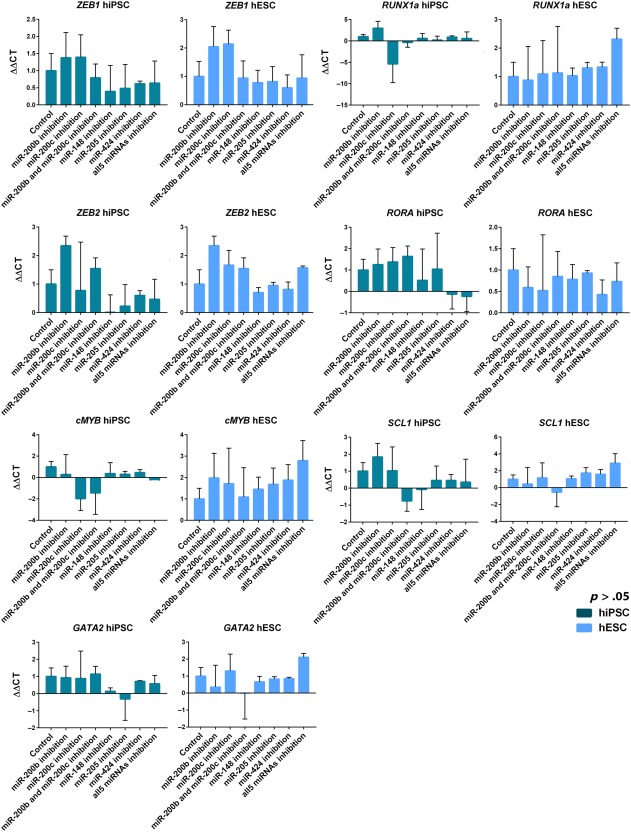
Impact of miRNA downregulation on target gene expression. qRT‐PCR data showing expression of key epithelial‐mesenchymal transition and hematopoiesis associated genes in hiPSCs and hESCs at day 12, 48 hours after inhibition. Data are shown as mean ΔΔCT values ± SEM, *n* = 3. Abbreviations: hESC, human embryonic stem cells; hiPSC, human induced pluripotent stem cell.

**Figure 5:**
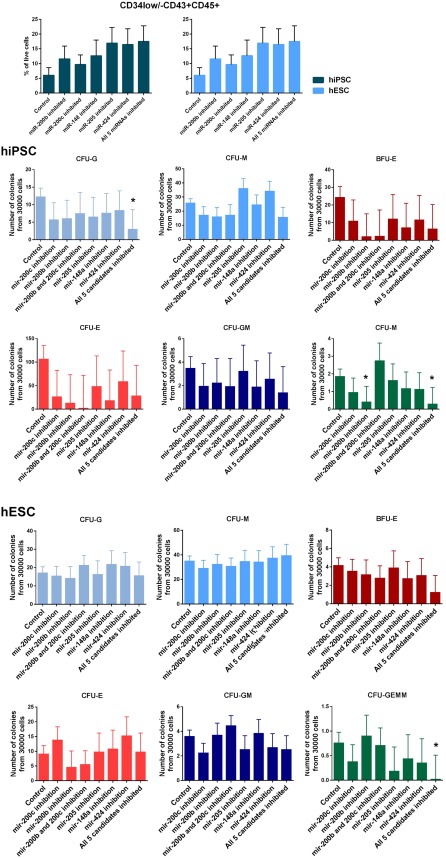
Impacts of miRNA downregulation on hematopoietic differentiation of hESC and hiPSC**. (A):** Flow cytometry data: the percentage of CD34low/‐CD43 + CD45+ cells at day 12 of differentiation after miRNA inhibition. Data are shown as mean % of live cells ± SEM, *n* = 3. **(B):** Schematic charts showing the impact of miRNA downregulation on various types of CFCs obtained from the differentiation of hESC and hiPSC lines. Data are shown as mean ±SEM, *n* = 3, *, *p* < .05. Abbreviations: BFU‐E, burst forming unit‐erythroid; CFU‐E, colony forming unit erythroid; CFU‐G, colony forming unit granulocyte; CFU‐M, colony forming unit macrophage; CFU‐GM, colony forming unit granulocyte‐macrphage; CFU‐GEMM, colony forming unit granulocyte‐erythrocyte‐monocyte‐macrphage; hESC, human embryonic stem cells; hiPSC, human induced pluripotent stem cell.

Although the expression of target miRNAs was robustly inhibited, the effect on the expression of key EMT‐associated transcription factors which are targets of some or all of the inhibited miRNAs was positive but fell short of being statistically significant (see Fig. 4); this may be due to the function of miRNAs as modulators, which fine tune expression of multiple genes but do not have a large impact on any individual gene. Neither did it have a significant effect on numbers of committed hematopoietic progenitors (CD34low/‐CD43 + CD45+), (Fig. 5A). Few colony types were significantly different in cells inhibited with any of the miRNAs, in fact the only significant change which occurs in both cell lines is in colony forming unit granulocyte, erythrocyte, monocyte, megakaryocyte (CFU)‐GEMM potential, which is significantly decreased in both hESCs and hiPSCs (Fig. 5B). This is the opposite result to what was anticipated as it indicates a decrease in the fraction of multi‐potent hematopoietic progenitors (Fig. 5B). The results show that the hESC line produces more colonies overall, but variations in the differentiation capacity of different pluripotent stem cell lines are an established factor in stem cell research, and have been shown to be due to epigenetic factors. For example, the chromatin accessibility in the genes of the insulin‐like growth factor 2 pathway affects the hematopoietic differentiation of hiPSC and hESC lines 30.

## Discussion

We have demonstrated that EMT‐suppressing miRNAs are expressed at aberrantly high levels in P‐HPCs, which likely negatively affects their success in vivo. However, inhibiting these miRNAs is insufficient to improve hematopoietic colony formation in vitro so it is likely, though not guaranteed, that inhibition of these miRNAs would produce no functional improvements in vivo.

There are several possible explanations for this negative result. First, there is a great deal of redundancy between miRNAs suppressing the same EMT pathways, as can be seen in the network analysis data, so inhibiting only five of them may not have been sufficient. Second, miRNAs are modulators of gene expression, with many only having a small effect on any individual gene's expression. These miRNAs are also part of a network which contains stabilizing feedback loops, for example the ZEB proteins targeted by the miR‐200 cluster inhibit the expression of the miR‐200 cluster as well as several other miRNAs 29. This produces a compensatory effect, so that if one EMT suppressor is inhibited others can be upregulated. Sugimura et al. recently showed that it is possible to derive functional HSCs from pluripotent stem cells; however, it required them to ectopically express seven transcription factors and the final step of the process was carried out in the in vivo bone marrow niche 31. miRNAs are more subtle in their effect, and this article shows the necessity of more drastic interventions.

What is not clear is why attempting to induce HSC emergence causes expression of these EMT‐inhibiting miRNAs. HSC generation is a unique developmental process in that it involves self‐renewing cells arising from the endothelium and achieving the ability to migrate independently of contact with the tissue of origin. This process must be precisely controlled because in any other circumstances self‐renewing cells moving freely around the body is equivalent to metastasis. This is reflected in the precise control of HSC emergence in the embryo; although hematopoietic cells arise from blood vessels throughout the developing embryo and extra‐embryonic tissues 32, 33, 34, 35, hematopoietic cells capable of indefinite self‐renewal and hematopoietic reconstitution arise in a narrow time frame and small anatomical area 16, 36. The over‐expression of EMT‐inhibiting miRNAs in differentiation cultures can be explained by induction of a robust network of tumor suppressors in response to self‐renewing cells attempting to leave their tissue of origin.

Given the similar expression patterns and functions of the mis‐expressed miRNAs it is possible that there is a common regulatory element or system which is controlling their expression. Further study will be needed to elucidate this mechanism. It is clear that the current in vitro conditions in which we differentiate P‐HPCs are not effective in creating HSCs, although we know it is possible to generate HSC from pluripotent stem cells by introducing the undifferentiated cells into a rodent model along with agents that promote hematopoiesis 37, 38, or by transplanting cells before day 3 of differentiation and allowing them to develop in vivo 39. This shows that pluripotent stem cells have the potential to form HSC but that this potential is lost very early in differentiation using current protocols, suggesting that a more precise combination of signals is required, but although there has been recent progress 34, the combination has not yet been discovered.

## Author Contributions

E.M.: performed the experimental work, data analysis, manuscript writing; D.M. and T.B.: data analysis; D.M.‐S., K.T., and E.A.H.: performed experimental work; L.A. conception and design, fund raising, and manuscript writing; M.L.: conception and design, data analysis, manuscript writing, and raised funds for this work. E.M., D.M., T.B., D.M.‐S., K.T., E.A.H., L.A., and M.L.: final approval of manuscript.

## Disclosure of Potential Conflicts of Interest

The authors indicated no potential conflicts of interest.

## Supporting information

Supplementary Figure 1

Supplementary Table 1

Supplementary Table 2

Supplementary Table 3
