# Supplementary figures and images for: Pluripotent Stem Cell‐Derived Hematopoietic Progenitors Are Unable to Downregulate Key Epithelial‐Mesenchymal Transition‐Associated miRNAs
Source: Stem Cells. 2017 Oct 27;36(1):55–64. doi: 10.1002/stem.2724 (PMC5765482; doi:10.1002/stem.2724)

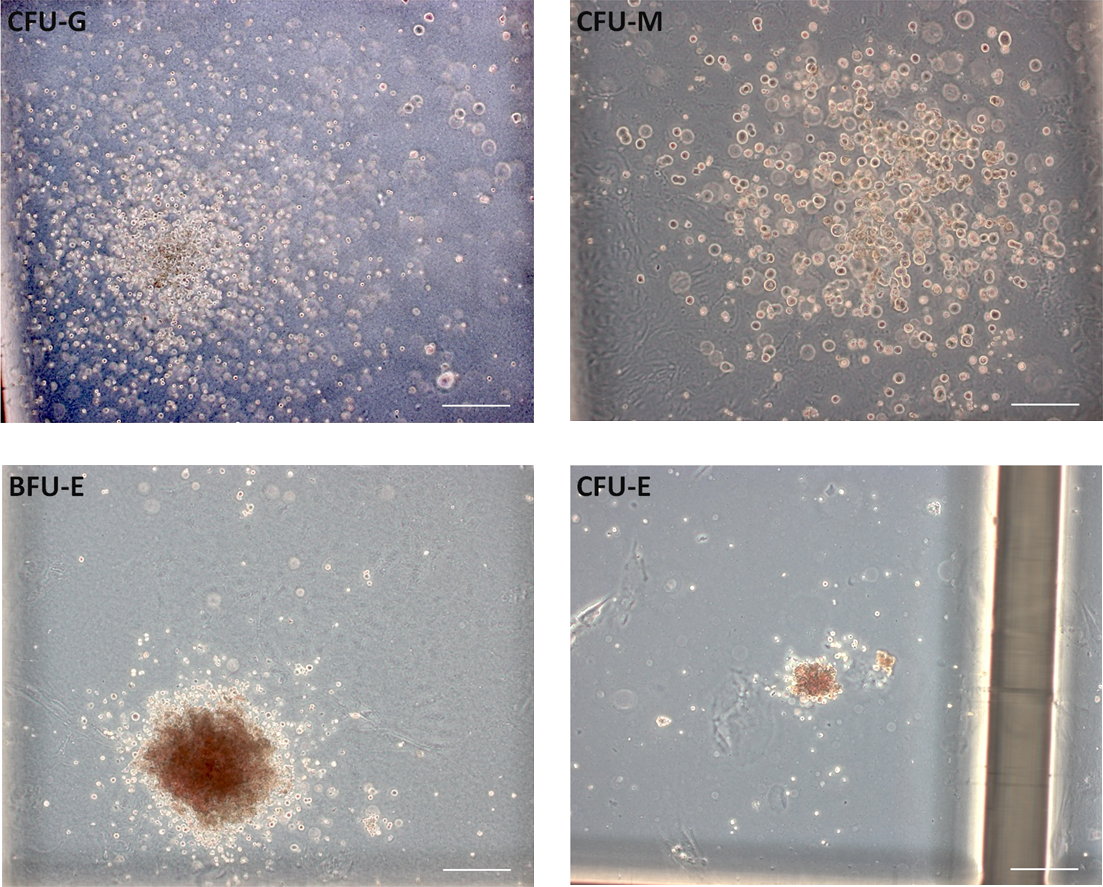

Supplement: Supplementary file 1 — Supplementary Figure 1 [file STEM-36-55-s001.tif]
