# Supplementary material for: Pluripotent Stem Cell‐Derived Hematopoietic Progenitors Are Unable to Downregulate Key Epithelial‐Mesenchymal Transition‐Associated miRNAs
Source: Stem Cells. 2017 Oct 27;36(1):55–64. doi: 10.1002/stem.2724 (PMC5765482; doi:10.1002/stem.2724)
Supplement: Supplementary file 2 — Supplementary Table 1 [file STEM-36-55-s002.docx]

|  | esc1 | esc2 | shc4 | cd34 | logFC : bm - ha | t : bm - ha | p.value : bm - ha | p.adj : bm - ha | logFC : ha - em | t : ha - em | p.value : ha - em | p.adj : ha - em | logFC : bm - em | t : bm - em | p.value : bm - em | p.adj : bm - em | SIGNIF : bm - ha | SIGNIF : ha - em | SIGNIF : bm - em | PATTERN |
| --- | --- | --- | --- | --- | --- | --- | --- | --- | --- | --- | --- | --- | --- | --- | --- | --- | --- | --- | --- | --- |
| hsa-miR-10b | 0.044 | 0.217 | 4.246 | 7.814 | 3.568 | 8.655 | 0.002 | 0.033 | 4.115 | 11.525 | 0.001 | 0.024 | 7.684 | 21.519 | 0.000 | 0.004 | 1 | 1 | 1 | up_up |
| hsa-miR-186 | 1.588 | 1.254 | 5.102 | 8.929 | 3.826 | 8.717 | 0.001 | 0.033 | 3.681 | 9.684 | 0.001 | 0.034 | 7.508 | 19.749 | 0.000 | 0.005 | 1 | 1 | 1 | up_up |
| hsa-miR-451 | 0.523 | -0.020 | 5.233 | 13.306 | 8.073 | 16.375 | 0.000 | 0.009 | 4.981 | 11.666 | 0.001 | 0.024 | 13.055 | 30.574 | 0.000 | 0.002 | 1 | 1 | 1 | up_up |
| hsa-let-7b | 7.003 | 8.364 | 2.693 | 13.025 | 10.332 | 12.598 | 0.000 | 0.015 | -4.990 | -7.026 | 0.003 | 0.073 | 5.341 | 7.520 | 0.002 | 0.042 | 1 | 0 | 1 | flat_up |
| hsa-let-7b* | 3.153 | 3.273 | 4.513 | 9.085 | 4.572 | 11.229 | 0.001 | 0.019 | 1.300 | 3.687 | 0.025 | 0.242 | 5.872 | 16.652 | 0.000 | 0.006 | 1 | 0 | 1 | flat_up |
| hsa-let-7c | 3.935 | 3.205 | 1.635 | 11.299 | 9.664 | 17.393 | 0.000 | 0.008 | -1.935 | -4.022 | 0.019 | 0.206 | 7.729 | 16.062 | 0.000 | 0.007 | 1 | 0 | 1 | flat_up |
| hsa-let-7d | 2.390 | 1.556 | 1.099 | 12.469 | 11.370 | 19.120 | 0.000 | 0.007 | -0.873 | -1.696 | 0.172 | 0.884 | 10.496 | 20.382 | 0.000 | 0.005 | 1 | 0 | 1 | flat_up |
| hsa-let-7d* | 1.727 | 1.990 | 0.084 | 4.513 | 4.430 | 10.416 | 0.001 | 0.022 | -1.775 | -4.820 | 0.011 | 0.147 | 2.654 | 7.207 | 0.003 | 0.047 | 1 | 0 | 1 | flat_up |
| hsa-let-7f-1* | 2.894 | 2.735 | 3.465 | 8.252 | 4.787 | 11.652 | 0.001 | 0.017 | 0.651 | 1.830 | 0.149 | 0.795 | 5.438 | 15.284 | 0.000 | 0.007 | 1 | 0 | 1 | flat_up |
| hsa-let-7g | 5.330 | 4.630 | 1.496 | 12.090 | 10.594 | 19.449 | 0.000 | 0.007 | -3.484 | -7.385 | 0.003 | 0.066 | 7.110 | 15.072 | 0.000 | 0.007 | 1 | 0 | 1 | flat_up |
| hsa-miR-10a* | -2.276 | -1.763 | -0.913 | 2.693 | 3.606 | 7.449 | 0.003 | 0.048 | 1.107 | 2.641 | 0.064 | 0.443 | 4.713 | 11.242 | 0.001 | 0.015 | 1 | 0 | 1 | flat_up |
| hsa-miR-1225-3p | 2.848 | 2.818 | 4.695 | 8.112 | 3.417 | 8.489 | 0.002 | 0.035 | 1.862 | 5.341 | 0.008 | 0.120 | 5.279 | 15.143 | 0.000 | 0.007 | 1 | 0 | 1 | flat_up |
| hsa-miR-1237 | 1.264 | 1.545 | 2.453 | 6.080 | 3.627 | 8.466 | 0.002 | 0.035 | 1.049 | 2.827 | 0.053 | 0.391 | 4.676 | 12.603 | 0.000 | 0.012 | 1 | 0 | 1 | flat_up |
| hsa-miR-1238 | 5.894 | 5.396 | 7.093 | 10.798 | 3.705 | 7.723 | 0.002 | 0.044 | 1.447 | 3.484 | 0.030 | 0.271 | 5.153 | 12.402 | 0.000 | 0.012 | 1 | 0 | 1 | flat_up |
| hsa-miR-127-3p | 0.291 | 0.033 | -0.049 | 3.843 | 3.892 | 9.169 | 0.001 | 0.030 | -0.211 | -0.573 | 0.600 | 1.000 | 3.681 | 10.015 | 0.001 | 0.020 | 1 | 0 | 1 | flat_up |
| hsa-miR-138-2* | -1.218 | -1.804 | -2.483 | 5.438 | 7.922 | 15.642 | 0.000 | 0.009 | -0.972 | -2.216 | 0.098 | 0.593 | 6.950 | 15.846 | 0.000 | 0.007 | 1 | 0 | 1 | flat_up |
| hsa-miR-142-3p | 0.017 | -0.126 | 0.163 | 13.143 | 12.981 | 31.721 | 0.000 | 0.005 | 0.217 | 0.612 | 0.577 | 1.000 | 13.197 | 37.241 | 0.000 | 0.002 | 1 | 0 | 1 | flat_up |
| hsa-miR-142-5p | -1.591 | -2.136 | -1.263 | 12.990 | 14.253 | 28.878 | 0.000 | 0.005 | 0.601 | 1.405 | 0.240 | 1.000 | 14.854 | 34.751 | 0.000 | 0.002 | 1 | 0 | 1 | flat_up |
| hsa-miR-146a | 1.519 | 1.318 | 2.965 | 11.170 | 8.205 | 19.736 | 0.000 | 0.007 | 1.547 | 4.296 | 0.016 | 0.182 | 9.752 | 27.086 | 0.000 | 0.003 | 1 | 0 | 1 | flat_up |
| hsa-miR-146b-5p | 0.418 | 0.182 | 2.325 | 11.396 | 9.070 | 21.546 | 0.000 | 0.006 | 2.025 | 5.556 | 0.007 | 0.112 | 11.096 | 30.435 | 0.000 | 0.002 | 1 | 0 | 1 | flat_up |
| hsa-miR-1470 | 1.489 | 1.727 | 1.153 | 5.102 | 3.949 | 9.373 | 0.001 | 0.028 | -0.455 | -1.246 | 0.287 | 1.000 | 3.494 | 9.577 | 0.001 | 0.022 | 1 | 0 | 1 | flat_up |
| hsa-miR-150 | 0.423 | 0.608 | -1.142 | 13.961 | 15.103 | 36.493 | 0.000 | 0.005 | -1.657 | -4.624 | 0.012 | 0.159 | 13.446 | 37.514 | 0.000 | 0.002 | 1 | 0 | 1 | flat_up |
| hsa-miR-1539 | 2.068 | 2.533 | 2.583 | 6.382 | 3.799 | 8.076 | 0.002 | 0.039 | 0.282 | 0.692 | 0.531 | 1.000 | 4.081 | 10.017 | 0.001 | 0.020 | 1 | 0 | 1 | flat_up |
| hsa-miR-155 | 4.747 | 4.348 | 7.453 | 11.842 | 4.389 | 9.680 | 0.001 | 0.025 | 2.905 | 7.398 | 0.003 | 0.066 | 7.294 | 18.575 | 0.000 | 0.005 | 1 | 0 | 1 | flat_up |
| hsa-miR-16-2* | 1.600 | 0.898 | 2.157 | 7.059 | 4.902 | 8.990 | 0.001 | 0.031 | 0.908 | 1.922 | 0.134 | 0.742 | 5.810 | 12.302 | 0.000 | 0.012 | 1 | 0 | 1 | flat_up |
| hsa-miR-181a* | 0.886 | 0.388 | 0.908 | 12.866 | 11.958 | 24.917 | 0.000 | 0.005 | 0.271 | 0.651 | 0.554 | 1.000 | 12.229 | 29.422 | 0.000 | 0.002 | 1 | 0 | 1 | flat_up |
| hsa-miR-181a-2* | 1.099 | 1.035 | 2.894 | 7.093 | 4.199 | 10.402 | 0.001 | 0.022 | 1.827 | 5.226 | 0.008 | 0.125 | 6.025 | 17.237 | 0.000 | 0.006 | 1 | 0 | 1 | flat_up |
| hsa-miR-181b | 7.182 | 7.182 | 9.279 | 13.681 | 4.402 | 10.943 | 0.001 | 0.019 | 2.097 | 6.020 | 0.005 | 0.100 | 6.499 | 18.656 | 0.000 | 0.005 | 1 | 0 | 1 | flat_up |
| hsa-miR-181c | 0.115 | -0.648 | 0.103 | 12.142 | 12.039 | 21.195 | 0.000 | 0.007 | 0.369 | 0.751 | 0.499 | 1.000 | 12.408 | 25.225 | 0.000 | 0.003 | 1 | 0 | 1 | flat_up |
| hsa-miR-191* | 3.961 | 3.576 | 5.657 | 9.502 | 3.844 | 8.541 | 0.002 | 0.035 | 1.889 | 4.846 | 0.011 | 0.147 | 5.733 | 14.708 | 0.000 | 0.008 | 1 | 0 | 1 | flat_up |
| hsa-miR-1913 | -0.489 | -0.146 | -1.546 | 3.890 | 5.436 | 12.330 | 0.000 | 0.016 | -1.229 | -3.219 | 0.037 | 0.300 | 4.207 | 11.019 | 0.001 | 0.016 | 1 | 0 | 1 | flat_up |
| hsa-miR-202 | 0.121 | 0.902 | 0.523 | 4.747 | 4.224 | 7.349 | 0.003 | 0.049 | 0.011 | 0.023 | 0.983 | 1.000 | 4.235 | 8.508 | 0.002 | 0.032 | 1 | 0 | 1 | flat_up |
| hsa-miR-212 | 1.153 | 1.335 | 2.706 | 12.067 | 9.361 | 22.645 | 0.000 | 0.006 | 1.461 | 4.082 | 0.018 | 0.200 | 10.823 | 30.230 | 0.000 | 0.002 | 1 | 0 | 1 | flat_up |
| hsa-miR-221* | 3.273 | 2.848 | -0.079 | 5.894 | 5.973 | 12.983 | 0.000 | 0.014 | -3.139 | -7.880 | 0.002 | 0.057 | 2.833 | 7.111 | 0.003 | 0.049 | 1 | 0 | 1 | flat_up |
| hsa-miR-223 | 2.012 | 1.466 | 5.201 | 12.727 | 7.526 | 15.230 | 0.000 | 0.009 | 3.462 | 8.090 | 0.002 | 0.053 | 10.988 | 25.677 | 0.000 | 0.003 | 1 | 0 | 1 | flat_up |
| hsa-miR-223* | -1.659 | -1.565 | 0.093 | 4.358 | 4.265 | 10.525 | 0.001 | 0.021 | 1.705 | 4.858 | 0.011 | 0.147 | 5.970 | 17.011 | 0.000 | 0.006 | 1 | 0 | 1 | flat_up |
| hsa-miR-23a* | -1.092 | -0.383 | 1.478 | 8.011 | 6.533 | 11.918 | 0.000 | 0.017 | 2.216 | 4.668 | 0.012 | 0.158 | 8.749 | 18.429 | 0.000 | 0.005 | 1 | 0 | 1 | flat_up |
| hsa-miR-338-3p | 1.073 | 0.485 | 1.564 | 7.399 | 5.835 | 11.509 | 0.001 | 0.018 | 0.785 | 1.789 | 0.155 | 0.817 | 6.620 | 15.079 | 0.000 | 0.007 | 1 | 0 | 1 | flat_up |
| hsa-miR-338-5p | 0.093 | 1.025 | -1.248 | 8.890 | 10.138 | 16.002 | 0.000 | 0.009 | -1.806 | -3.292 | 0.035 | 0.295 | 8.332 | 15.185 | 0.000 | 0.007 | 1 | 0 | 1 | flat_up |
| hsa-miR-361-3p | 1.201 | 0.416 | 2.488 | 8.060 | 5.573 | 9.674 | 0.001 | 0.025 | 1.679 | 3.365 | 0.033 | 0.286 | 7.252 | 14.535 | 0.000 | 0.008 | 1 | 0 | 1 | flat_up |
| hsa-miR-376c | 0.036 | -0.855 | -1.117 | 3.907 | 5.024 | 8.142 | 0.002 | 0.038 | -0.707 | -1.324 | 0.263 | 1.000 | 4.317 | 8.078 | 0.002 | 0.036 | 1 | 0 | 1 | flat_up |
| hsa-miR-378 | 2.111 | 1.582 | 1.285 | 9.556 | 8.272 | 16.923 | 0.000 | 0.008 | -0.562 | -1.327 | 0.262 | 1.000 | 7.710 | 18.213 | 0.000 | 0.005 | 1 | 0 | 1 | flat_up |
| hsa-miR-378* | 0.591 | 0.336 | -1.105 | 4.473 | 5.578 | 13.159 | 0.000 | 0.014 | -1.569 | -4.275 | 0.016 | 0.184 | 4.009 | 10.920 | 0.001 | 0.016 | 1 | 0 | 1 | flat_up |
| hsa-miR-431* | 0.070 | -0.475 | -0.431 | 5.051 | 5.482 | 11.104 | 0.001 | 0.019 | -0.228 | -0.533 | 0.625 | 1.000 | 5.254 | 12.288 | 0.000 | 0.012 | 1 | 0 | 1 | flat_up |
| hsa-miR-486-5p | 1.943 | 2.641 | 0.182 | 8.550 | 8.368 | 15.377 | 0.000 | 0.009 | -2.110 | -4.477 | 0.014 | 0.170 | 6.258 | 13.279 | 0.000 | 0.010 | 1 | 0 | 1 | flat_up |
| hsa-miR-564 | 0.054 | 0.070 | 0.858 | 7.003 | 6.145 | 15.275 | 0.000 | 0.009 | 0.796 | 2.284 | 0.091 | 0.559 | 6.941 | 19.921 | 0.000 | 0.005 | 1 | 0 | 1 | flat_up |
| hsa-miR-634 | 1.318 | 2.068 | 1.876 | 6.180 | 4.303 | 7.644 | 0.002 | 0.045 | 0.183 | 0.375 | 0.728 | 1.000 | 4.486 | 9.202 | 0.001 | 0.025 | 1 | 0 | 1 | flat_up |
| hsa-miR-650 | -0.260 | 0.030 | -0.317 | 7.453 | 7.769 | 18.062 | 0.000 | 0.008 | -0.202 | -0.541 | 0.620 | 1.000 | 7.567 | 20.315 | 0.000 | 0.005 | 1 | 0 | 1 | flat_up |
| hsa-miR-664 | 1.408 | 2.039 | 2.785 | 9.344 | 6.559 | 12.581 | 0.000 | 0.015 | 1.062 | 2.352 | 0.085 | 0.534 | 7.621 | 16.879 | 0.000 | 0.006 | 1 | 0 | 1 | flat_up |
| hsa-miR-665 | -0.166 | 0.050 | -1.399 | 2.785 | 4.185 | 10.012 | 0.001 | 0.023 | -1.341 | -3.705 | 0.025 | 0.241 | 2.844 | 7.856 | 0.002 | 0.038 | 1 | 0 | 1 | flat_up |
| hsa-miR-7-1* | 0.892 | 0.716 | 1.264 | 4.630 | 3.366 | 8.158 | 0.002 | 0.038 | 0.460 | 1.287 | 0.274 | 1.000 | 3.826 | 10.707 | 0.001 | 0.017 | 1 | 0 | 1 | flat_up |
| hsa-miR-766 | 10.676 | 10.307 | 8.342 | 16.195 | 7.854 | 17.583 | 0.000 | 0.008 | -2.150 | -5.558 | 0.007 | 0.112 | 5.704 | 14.746 | 0.000 | 0.008 | 1 | 0 | 1 | flat_up |
| hsa-miR-767-3p | 0.030 | 0.838 | -0.253 | 4.566 | 4.819 | 8.246 | 0.002 | 0.038 | -0.687 | -1.358 | 0.253 | 1.000 | 4.132 | 8.164 | 0.002 | 0.036 | 1 | 0 | 1 | flat_up |
| hsa-miR-770-5p | 0.042 | 0.678 | -0.363 | 8.660 | 9.024 | 17.256 | 0.000 | 0.008 | -0.723 | -1.598 | 0.193 | 0.959 | 8.300 | 18.328 | 0.000 | 0.005 | 1 | 0 | 1 | flat_up |
| hsa-miR-933 | 2.208 | 2.157 | 1.185 | 8.135 | 6.949 | 17.237 | 0.000 | 0.008 | -0.997 | -2.856 | 0.052 | 0.384 | 5.952 | 17.048 | 0.000 | 0.006 | 1 | 0 | 1 | flat_up |
| hsa-miR-98 | -0.845 | -0.485 | 0.044 | 7.707 | 7.663 | 17.241 | 0.000 | 0.008 | 0.710 | 1.844 | 0.146 | 0.786 | 8.373 | 21.752 | 0.000 | 0.004 | 1 | 0 | 1 | flat_up |
| hsa-miR-1306 | 1.967 | 2.368 | 0.470 | 3.935 | 3.465 | 7.634 | 0.002 | 0.045 | -1.697 | -4.318 | 0.015 | 0.181 | 1.767 | 4.497 | 0.014 | 0.146 | 1 | 0 | 0 | flat_up |
| hsa-miR-324-5p | 8.342 | 7.554 | 6.600 | 10.942 | 4.342 | 7.527 | 0.002 | 0.047 | -1.348 | -2.699 | 0.060 | 0.423 | 2.994 | 5.993 | 0.005 | 0.074 | 1 | 0 | 0 | flat_up |
| hsa-miR-487b | 2.735 | 5.004 | -0.436 | 9.892 | 10.328 | 8.212 | 0.002 | 0.038 | -4.306 | -3.953 | 0.020 | 0.215 | 6.023 | 5.530 | 0.007 | 0.089 | 1 | 0 | 0 | flat_up |
| hsa-miR-92b | 2.884 | 2.754 | 0.678 | 4.801 | 4.123 | 10.106 | 0.001 | 0.023 | -2.141 | -6.059 | 0.005 | 0.100 | 1.982 | 5.611 | 0.007 | 0.086 | 1 | 0 | 0 | flat_up |
| hsa-miR-1826 | 5.982 | 6.261 | 1.701 | 10.307 | 8.606 | 20.100 | 0.000 | 0.007 | -4.420 | -11.921 | 0.000 | 0.024 | 4.185 | 11.288 | 0.001 | 0.015 | 1 | -1 | 1 | down_up |
| hsa-miR-222 | 4.045 | 3.961 | 0.418 | 10.431 | 10.013 | 24.743 | 0.000 | 0.005 | -3.585 | -10.229 | 0.001 | 0.030 | 6.428 | 18.342 | 0.000 | 0.005 | 1 | -1 | 1 | down_up |
| hsa-let-7a | 12.872 | 12.490 | 8.060 | 13.076 | 5.015 | 11.159 | 0.001 | 0.019 | -4.620 | -11.871 | 0.000 | 0.024 | 0.395 | 1.014 | 0.373 | 1.000 | 1 | -1 | 0 | down_up |
| hsa-let-7e | 12.152 | 11.685 | 7.707 | 12.490 | 4.782 | 10.152 | 0.001 | 0.023 | -4.211 | -10.322 | 0.001 | 0.029 | 0.571 | 1.401 | 0.241 | 1.000 | 1 | -1 | 0 | down_up |
| hsa-let-7f | 9.863 | 9.679 | 5.330 | 11.096 | 5.766 | 13.936 | 0.000 | 0.012 | -4.441 | -12.395 | 0.000 | 0.024 | 1.325 | 3.697 | 0.025 | 0.225 | 1 | -1 | 0 | down_up |
| hsa-let-7i | 10.340 | 9.199 | 1.727 | 13.272 | 11.544 | 16.000 | 0.000 | 0.009 | -8.042 | -12.870 | 0.000 | 0.023 | 3.502 | 5.605 | 0.007 | 0.086 | 1 | -1 | 0 | down_up |
| hsa-miR-1305 | 12.216 | 12.976 | 6.832 | 13.840 | 7.008 | 12.375 | 0.000 | 0.016 | -5.764 | -11.752 | 0.001 | 0.024 | 1.244 | 2.537 | 0.071 | 0.473 | 1 | -1 | 0 | down_up |
| hsa-miR-21* | 7.453 | 7.093 | 1.191 | 6.794 | 5.603 | 12.608 | 0.000 | 0.015 | -6.082 | -15.801 | 0.000 | 0.016 | -0.479 | -1.243 | 0.288 | 1.000 | 1 | -1 | 0 | down_up |
| hsa-miR-29a | 12.866 | 12.990 | 8.550 | 12.216 | 3.666 | 8.997 | 0.001 | 0.031 | -4.378 | -12.405 | 0.000 | 0.024 | -0.712 | -2.017 | 0.121 | 0.689 | 1 | -1 | 0 | down_up |
| hsa-miR-29b | 8.890 | 9.085 | 4.105 | 9.012 | 4.907 | 11.824 | 0.001 | 0.017 | -4.883 | -13.586 | 0.000 | 0.021 | 0.024 | 0.067 | 0.950 | 1.000 | 1 | -1 | 0 | down_up |
| hsa-miR-551b | 13.260 | 11.891 | 3.702 | 14.928 | 11.226 | 13.625 | 0.000 | 0.012 | -8.874 | -12.436 | 0.000 | 0.024 | 2.352 | 3.296 | 0.035 | 0.282 | 1 | -1 | 0 | down_up |
| hsa-miR-100 | 12.794 | 12.507 | 3.843 | 10.046 | 6.203 | 14.441 | 0.000 | 0.011 | -8.808 | -23.678 | 0.000 | 0.007 | -2.605 | -7.003 | 0.003 | 0.050 | 1 | -1 | -1 | down_up |
| hsa-miR-10a | 0.365 | 0.443 | 11.657 | 13.260 | 1.604 | 3.967 | 0.020 | 0.198 | 11.253 | 32.141 | 0.000 | 0.007 | 12.856 | 36.721 | 0.000 | 0.002 | 0 | 1 | 1 | up_flat |
| hsa-miR-126 | 7.059 | 6.382 | 12.773 | 12.170 | -0.604 | -1.124 | 0.330 | 1.000 | 6.053 | 13.020 | 0.000 | 0.022 | 5.449 | 11.722 | 0.001 | 0.014 | 0 | 1 | 1 | up_flat |
| hsa-miR-126* | -0.146 | -0.845 | 11.396 | 7.856 | -3.540 | -6.497 | 0.004 | 0.065 | 11.891 | 25.202 | 0.000 | 0.007 | 8.352 | 17.701 | 0.000 | 0.006 | 0 | 1 | 1 | up_flat |
| hsa-miR-140-3p | 5.657 | 5.330 | 8.820 | 11.475 | 2.656 | 6.070 | 0.005 | 0.076 | 3.326 | 8.779 | 0.001 | 0.045 | 5.982 | 15.788 | 0.000 | 0.007 | 0 | 1 | 1 | up_flat |
| hsa-miR-196b | 0.359 | 0.375 | 9.863 | 12.523 | 2.660 | 6.612 | 0.004 | 0.062 | 9.496 | 27.256 | 0.000 | 0.007 | 12.156 | 34.890 | 0.000 | 0.002 | 0 | 1 | 1 | up_flat |
| hsa-miR-30c | 6.832 | 6.731 | 10.862 | 12.269 | 1.407 | 3.467 | 0.030 | 0.262 | 4.080 | 11.612 | 0.001 | 0.024 | 5.487 | 15.615 | 0.000 | 0.007 | 0 | 1 | 1 | up_flat |
| hsa-miR-30d | 6.300 | 5.549 | 12.638 | 13.926 | 1.288 | 2.286 | 0.091 | 0.573 | 6.714 | 13.758 | 0.000 | 0.020 | 8.002 | 16.397 | 0.000 | 0.007 | 0 | 1 | 1 | up_flat |
| hsa-miR-148b | 5.549 | 5.438 | 8.620 | 7.531 | -1.088 | -2.678 | 0.061 | 0.430 | 3.126 | 8.881 | 0.001 | 0.044 | 2.037 | 5.789 | 0.006 | 0.080 | 0 | 1 | 0 | up_flat |
| hsa-miR-194 | -0.060 | 0.243 | 4.348 | 1.341 | -3.008 | -6.953 | 0.003 | 0.057 | 4.256 | 11.363 | 0.001 | 0.024 | 1.249 | 3.334 | 0.034 | 0.279 | 0 | 1 | 0 | up_flat |
| hsa-miR-452 | -1.851 | -1.795 | 1.524 | -1.242 | -2.766 | -6.859 | 0.003 | 0.058 | 3.347 | 9.583 | 0.001 | 0.034 | 0.581 | 1.664 | 0.179 | 0.934 | 0 | 1 | 0 | up_flat |
| hsa-miR-502-3p | -0.002 | -0.323 | 4.074 | 1.086 | -2.988 | -6.851 | 0.003 | 0.058 | 4.236 | 11.215 | 0.001 | 0.024 | 1.248 | 3.305 | 0.034 | 0.282 | 0 | 1 | 0 | up_flat |
| hsa-miR-502-5p | 0.403 | 0.447 | 3.674 | 1.419 | -2.255 | -5.597 | 0.007 | 0.092 | 3.249 | 9.311 | 0.001 | 0.038 | 0.993 | 2.847 | 0.052 | 0.374 | 0 | 1 | 0 | up_flat |
| hsa-miR-532-3p | 0.791 | -0.106 | 5.975 | 3.113 | -2.862 | -4.619 | 0.012 | 0.146 | 5.632 | 10.498 | 0.001 | 0.029 | 2.770 | 5.164 | 0.009 | 0.105 | 0 | 1 | 0 | up_flat |
| hsa-miR-92a-1* | 0.478 | 0.428 | 4.422 | 2.735 | -1.688 | -4.186 | 0.017 | 0.174 | 3.969 | 11.370 | 0.001 | 0.024 | 2.282 | 6.536 | 0.004 | 0.059 | 0 | 1 | 0 | up_flat |
| hsa-miR-1227 | 1.367 | 1.519 | 1.173 | 3.989 | 2.816 | 6.868 | 0.003 | 0.058 | -0.270 | -0.759 | 0.494 | 1.000 | 2.546 | 7.171 | 0.003 | 0.048 | 0 | 0 | 1 | flat_flat |
| hsa-miR-1228 | 7.554 | 7.219 | 9.403 | 10.517 | 1.114 | 2.537 | 0.071 | 0.474 | 2.016 | 5.302 | 0.008 | 0.123 | 3.130 | 8.231 | 0.002 | 0.035 | 0 | 0 | 1 | flat_flat |
| hsa-miR-128 | 8.060 | 7.925 | 9.344 | 12.111 | 2.767 | 6.773 | 0.004 | 0.059 | 1.351 | 3.820 | 0.022 | 0.229 | 4.118 | 11.640 | 0.001 | 0.014 | 0 | 0 | 1 | flat_flat |
| hsa-miR-129* | 1.054 | 1.185 | 2.754 | 4.045 | 1.291 | 3.164 | 0.039 | 0.315 | 1.634 | 4.624 | 0.012 | 0.159 | 2.925 | 8.277 | 0.002 | 0.035 | 0 | 0 | 1 | flat_flat |
| hsa-miR-181a | 7.219 | 6.669 | 9.457 | 12.638 | 3.181 | 6.425 | 0.004 | 0.066 | 2.513 | 5.861 | 0.006 | 0.105 | 5.694 | 13.280 | 0.000 | 0.010 | 0 | 0 | 1 | flat_flat |
| hsa-miR-185 | 6.119 | 5.102 | 8.437 | 10.267 | 1.829 | 2.737 | 0.058 | 0.420 | 2.827 | 4.884 | 0.010 | 0.146 | 4.656 | 8.045 | 0.002 | 0.036 | 0 | 0 | 1 | flat_flat |
| hsa-miR-23b | 9.128 | 8.820 | 11.982 | 11.635 | -0.347 | -0.801 | 0.473 | 1.000 | 3.008 | 8.012 | 0.002 | 0.055 | 2.661 | 7.088 | 0.003 | 0.049 | 0 | 0 | 1 | flat_flat |
| hsa-miR-29c | 8.364 | 7.059 | 10.517 | 14.392 | 3.875 | 4.877 | 0.010 | 0.130 | 2.805 | 4.076 | 0.018 | 0.200 | 6.681 | 9.707 | 0.001 | 0.021 | 0 | 0 | 1 | flat_flat |
| hsa-miR-324-3p | 10.426 | 10.267 | 11.635 | 14.220 | 2.585 | 6.293 | 0.005 | 0.069 | 1.289 | 3.623 | 0.026 | 0.251 | 3.874 | 10.889 | 0.001 | 0.016 | 0 | 0 | 1 | flat_flat |
| hsa-miR-335* | 2.039 | 1.073 | 1.341 | 5.613 | 4.272 | 6.596 | 0.004 | 0.062 | -0.215 | -0.384 | 0.722 | 1.000 | 4.057 | 7.233 | 0.003 | 0.047 | 0 | 0 | 1 | flat_flat |
| hsa-miR-342-5p | 0.867 | 0.591 | 3.248 | 5.073 | 1.825 | 4.269 | 0.016 | 0.170 | 2.519 | 6.804 | 0.003 | 0.078 | 4.344 | 11.734 | 0.001 | 0.014 | 0 | 0 | 1 | flat_flat |
| hsa-miR-423-3p | 0.501 | 0.322 | 1.466 | 3.082 | 1.616 | 3.912 | 0.021 | 0.203 | 1.055 | 2.948 | 0.047 | 0.359 | 2.671 | 7.466 | 0.003 | 0.043 | 0 | 0 | 1 | flat_flat |
| hsa-miR-423-5p | 4.473 | 5.142 | 7.881 | 9.998 | 2.117 | 3.963 | 0.020 | 0.198 | 3.074 | 6.645 | 0.004 | 0.082 | 5.190 | 11.221 | 0.001 | 0.015 | 0 | 0 | 1 | flat_flat |
| hsa-miR-425* | 2.269 | 1.683 | 3.544 | 6.464 | 2.920 | 5.769 | 0.006 | 0.086 | 1.568 | 3.577 | 0.027 | 0.258 | 4.488 | 10.239 | 0.001 | 0.019 | 0 | 0 | 1 | flat_flat |
| hsa-miR-484 | 4.105 | 3.248 | 7.003 | 9.199 | 2.196 | 3.640 | 0.026 | 0.238 | 3.326 | 6.366 | 0.004 | 0.092 | 5.523 | 10.569 | 0.001 | 0.017 | 0 | 0 | 1 | flat_flat |
| hsa-miR-550 | 1.800 | 0.867 | 1.830 | 5.657 | 3.827 | 6.037 | 0.005 | 0.077 | 0.496 | 0.904 | 0.422 | 1.000 | 4.324 | 7.874 | 0.002 | 0.038 | 0 | 0 | 1 | flat_flat |
| hsa-miR-550* | 2.965 | 2.303 | 4.566 | 6.911 | 2.346 | 4.412 | 0.014 | 0.160 | 1.931 | 4.195 | 0.017 | 0.191 | 4.277 | 9.289 | 0.001 | 0.024 | 0 | 0 | 1 | flat_flat |
| hsa-miR-582-5p | 0.847 | 0.789 | 2.848 | 3.296 | 0.448 | 1.111 | 0.335 | 1.000 | 2.031 | 5.813 | 0.006 | 0.106 | 2.479 | 7.096 | 0.003 | 0.049 | 0 | 0 | 1 | flat_flat |
| hsa-miR-625 | 3.113 | 2.269 | 5.982 | 6.731 | 0.749 | 1.252 | 0.285 | 1.000 | 3.291 | 6.347 | 0.004 | 0.092 | 4.040 | 7.792 | 0.002 | 0.039 | 0 | 0 | 1 | flat_flat |
| hsa-miR-652 | 2.533 | 2.208 | 3.961 | 5.233 | 1.272 | 2.912 | 0.049 | 0.372 | 1.590 | 4.202 | 0.017 | 0.191 | 2.862 | 7.565 | 0.002 | 0.042 | 0 | 0 | 1 | flat_flat |
| hsa-miR-99a | 6.600 | 5.734 | 9.892 | 11.982 | 2.090 | 3.443 | 0.031 | 0.264 | 3.725 | 7.087 | 0.003 | 0.072 | 5.815 | 11.063 | 0.001 | 0.015 | 0 | 0 | 1 | flat_flat |
| hsa-let-7a* | 0.086 | 0.705 | -0.867 | 2.848 | 3.715 | 7.183 | 0.003 | 0.052 | -1.262 | -2.818 | 0.054 | 0.394 | 2.453 | 5.476 | 0.007 | 0.090 | 0 | 0 | 0 | flat_flat |
| hsa-let-7c* | -1.051 | 0.067 | -2.056 | 2.641 | 4.697 | 6.602 | 0.004 | 0.062 | -1.564 | -2.539 | 0.070 | 0.476 | 3.133 | 5.084 | 0.009 | 0.108 | 0 | 0 | 0 | flat_flat |
| hsa-let-7e* | 1.139 | 0.642 | -0.652 | 0.892 | 1.543 | 3.217 | 0.037 | 0.306 | -1.542 | -3.712 | 0.025 | 0.241 | 0.001 | 0.003 | 0.998 | 1.000 | 0 | 0 | 0 | flat_flat |
| hsa-let-7f-2* | -0.079 | -0.009 | -0.931 | 1.556 | 2.487 | 6.157 | 0.005 | 0.074 | -0.887 | -2.536 | 0.071 | 0.476 | 1.600 | 4.573 | 0.013 | 0.141 | 0 | 0 | 0 | flat_flat |
| hsa-let-7g* | 0.222 | -0.421 | -1.840 | -1.051 | 0.789 | 1.502 | 0.215 | 1.000 | -1.740 | -3.825 | 0.022 | 0.229 | -0.951 | -2.091 | 0.112 | 0.657 | 0 | 0 | 0 | flat_flat |
| hsa-let-7i* | -0.699 | -1.462 | -0.953 | 0.388 | 1.341 | 2.363 | 0.084 | 0.544 | 0.127 | 0.258 | 0.810 | 1.000 | 1.468 | 2.986 | 0.046 | 0.345 | 0 | 0 | 0 | flat_flat |
| hsa-miR-1 | 0.153 | -0.436 | 0.269 | -1.278 | -1.547 | -3.048 | 0.043 | 0.345 | 0.411 | 0.934 | 0.408 | 1.000 | -1.136 | -2.586 | 0.067 | 0.454 | 0 | 0 | 0 | flat_flat |
| hsa-miR-100* | -1.148 | -0.877 | 0.608 | -0.812 | -1.421 | -3.329 | 0.034 | 0.285 | 1.621 | 4.386 | 0.015 | 0.177 | 0.200 | 0.542 | 0.619 | 1.000 | 0 | 0 | 0 | flat_flat |
| hsa-miR-101 | 11.475 | 10.620 | 9.767 | 10.730 | 0.963 | 1.597 | 0.193 | 1.000 | -1.281 | -2.452 | 0.077 | 0.505 | -0.318 | -0.608 | 0.579 | 1.000 | 0 | 0 | 0 | flat_flat |
| hsa-miR-101* | -0.631 | -0.280 | -1.676 | -0.147 | 1.529 | 3.457 | 0.030 | 0.262 | -1.221 | -3.186 | 0.038 | 0.305 | 0.309 | 0.805 | 0.470 | 1.000 | 0 | 0 | 0 | flat_flat |
| hsa-miR-103 | 12.090 | 12.170 | 12.794 | 12.055 | -0.739 | -1.828 | 0.149 | 0.834 | 0.665 | 1.897 | 0.138 | 0.754 | -0.075 | -0.213 | 0.843 | 1.000 | 0 | 0 | 0 | flat_flat |
| hsa-miR-103-as | -0.087 | -0.769 | -0.632 | 0.325 | 0.957 | 1.776 | 0.158 | 0.877 | -0.204 | -0.437 | 0.687 | 1.000 | 0.753 | 1.614 | 0.189 | 0.970 | 0 | 0 | 0 | flat_flat |
| hsa-miR-105 | -0.228 | -0.096 | -2.190 | -0.644 | 1.546 | 3.788 | 0.023 | 0.216 | -2.028 | -5.737 | 0.006 | 0.108 | -0.482 | -1.363 | 0.251 | 1.000 | 0 | 0 | 0 | flat_flat |
| hsa-miR-105* | -1.226 | -1.625 | -0.260 | -1.314 | -1.054 | -2.324 | 0.088 | 0.556 | 1.165 | 2.966 | 0.047 | 0.356 | 0.111 | 0.282 | 0.793 | 1.000 | 0 | 0 | 0 | flat_flat |
| hsa-miR-106b | 12.773 | 12.794 | 13.076 | 12.794 | -0.281 | -0.699 | 0.527 | 1.000 | 0.292 | 0.837 | 0.454 | 1.000 | 0.011 | 0.030 | 0.977 | 1.000 | 0 | 0 | 0 | flat_flat |
| hsa-miR-106b* | -0.904 | -1.005 | 0.176 | 0.485 | 0.309 | 0.762 | 0.493 | 1.000 | 1.131 | 3.218 | 0.037 | 0.300 | 1.440 | 4.097 | 0.018 | 0.180 | 0 | 0 | 0 | flat_flat |
| hsa-miR-107 | 12.067 | 12.111 | 12.507 | 12.152 | -0.355 | -0.882 | 0.432 | 1.000 | 0.419 | 1.200 | 0.303 | 1.000 | 0.063 | 0.181 | 0.866 | 1.000 | 0 | 0 | 0 | flat_flat |
| hsa-miR-10b* | -0.804 | -0.293 | -0.009 | -0.208 | -0.198 | -0.410 | 0.705 | 1.000 | 0.539 | 1.289 | 0.274 | 1.000 | 0.341 | 0.815 | 0.465 | 1.000 | 0 | 0 | 0 | flat_flat |
| hsa-miR-1178 | -0.255 | 0.115 | -0.814 | 0.375 | 1.189 | 2.660 | 0.062 | 0.435 | -0.744 | -1.922 | 0.134 | 0.742 | 0.445 | 1.149 | 0.321 | 1.000 | 0 | 0 | 0 | flat_flat |
| hsa-miR-1179 | -0.343 | 0.423 | 0.243 | 0.336 | 0.093 | 0.164 | 0.878 | 1.000 | 0.203 | 0.412 | 0.704 | 1.000 | 0.296 | 0.601 | 0.583 | 1.000 | 0 | 0 | 0 | flat_flat |
| hsa-miR-1180 | 2.315 | 3.082 | 0.692 | 0.577 | -0.115 | -0.202 | 0.850 | 1.000 | -2.006 | -4.069 | 0.019 | 0.200 | -2.121 | -4.303 | 0.016 | 0.159 | 0 | 0 | 0 | flat_flat |
| hsa-miR-1182 | -0.539 | -0.904 | 1.313 | -0.597 | -1.910 | -4.288 | 0.016 | 0.169 | 2.035 | 5.274 | 0.008 | 0.124 | 0.124 | 0.322 | 0.765 | 1.000 | 0 | 0 | 0 | flat_flat |
| hsa-miR-1183 | 3.340 | 4.919 | 2.123 | 0.523 | -1.600 | -1.736 | 0.165 | 0.899 | -2.006 | -2.514 | 0.072 | 0.484 | -3.606 | -4.519 | 0.013 | 0.145 | 0 | 0 | 0 | flat_flat |
| hsa-miR-1184 | -0.238 | -0.951 | -0.574 | -0.265 | 0.310 | 0.564 | 0.606 | 1.000 | 0.021 | 0.043 | 0.968 | 1.000 | 0.330 | 0.694 | 0.529 | 1.000 | 0 | 0 | 0 | flat_flat |
| hsa-miR-1185 | -0.421 | -0.946 | -0.188 | 0.705 | 0.894 | 1.833 | 0.148 | 0.834 | 0.495 | 1.173 | 0.312 | 1.000 | 1.389 | 3.289 | 0.035 | 0.282 | 0 | 0 | 0 | flat_flat |
| hsa-miR-1197 | -0.572 | -0.670 | -0.648 | -0.807 | -0.159 | -0.392 | 0.717 | 1.000 | -0.028 | -0.079 | 0.941 | 1.000 | -0.187 | -0.531 | 0.626 | 1.000 | 0 | 0 | 0 | flat_flat |
| hsa-miR-1200 | -0.127 | -0.265 | -1.982 | 0.037 | 2.019 | 4.940 | 0.010 | 0.126 | -1.786 | -5.046 | 0.009 | 0.137 | 0.233 | 0.658 | 0.550 | 1.000 | 0 | 0 | 0 | flat_flat |
| hsa-miR-1201 | -1.162 | -1.248 | -0.376 | -0.151 | 0.225 | 0.557 | 0.610 | 1.000 | 0.828 | 2.364 | 0.084 | 0.531 | 1.054 | 3.007 | 0.045 | 0.340 | 0 | 0 | 0 | flat_flat |
| hsa-miR-1202 | 13.946 | 13.946 | 13.946 | 14.131 | 0.185 | 0.460 | 0.672 | 1.000 | 0.000 | 0.000 | 1.000 | 1.000 | 0.185 | 0.531 | 0.626 | 1.000 | 0 | 0 | 0 | flat_flat |
| hsa-miR-1203 | -0.652 | -0.892 | -1.643 | -0.823 | 0.820 | 1.944 | 0.131 | 0.761 | -0.871 | -2.385 | 0.082 | 0.523 | -0.051 | -0.140 | 0.896 | 1.000 | 0 | 0 | 0 | flat_flat |
| hsa-miR-1204 | -0.468 | -0.517 | -0.288 | -0.329 | -0.041 | -0.103 | 0.924 | 1.000 | 0.205 | 0.587 | 0.592 | 1.000 | 0.164 | 0.469 | 0.666 | 1.000 | 0 | 0 | 0 | flat_flat |
| hsa-miR-1205 | -1.487 | -1.405 | 0.253 | -0.892 | -1.145 | -2.830 | 0.053 | 0.398 | 1.699 | 4.850 | 0.011 | 0.147 | 0.554 | 1.582 | 0.196 | 0.986 | 0 | 0 | 0 | flat_flat |
| hsa-miR-1206 | -0.446 | -0.583 | -0.337 | -0.591 | -0.254 | -0.622 | 0.571 | 1.000 | 0.177 | 0.500 | 0.646 | 1.000 | -0.077 | -0.219 | 0.839 | 1.000 | 0 | 0 | 0 | flat_flat |
| hsa-miR-1207-3p | 0.449 | 0.395 | -0.719 | -0.446 | 0.274 | 0.679 | 0.538 | 1.000 | -1.141 | -3.266 | 0.036 | 0.298 | -0.867 | -2.483 | 0.074 | 0.489 | 0 | 0 | 0 | flat_flat |
| hsa-miR-1207-5p | 14.220 | 13.986 | 14.131 | 13.522 | -0.609 | -1.448 | 0.228 | 1.000 | 0.028 | 0.077 | 0.943 | 1.000 | -0.581 | -1.595 | 0.193 | 0.980 | 0 | 0 | 0 | flat_flat |
| hsa-miR-1208 | 1.526 | 2.012 | -0.066 | -0.749 | -0.683 | -1.433 | 0.232 | 1.000 | -1.835 | -4.445 | 0.014 | 0.172 | -2.518 | -6.100 | 0.005 | 0.071 | 0 | 0 | 0 | flat_flat |
| hsa-miR-1224-3p | 3.205 | 4.513 | -0.509 | 0.553 | 1.062 | 1.334 | 0.260 | 1.000 | -4.368 | -6.336 | 0.004 | 0.092 | -3.306 | -4.796 | 0.011 | 0.127 | 0 | 0 | 0 | flat_flat |
| hsa-miR-1225-5p | 13.986 | 13.961 | 14.220 | 13.986 | -0.235 | -0.583 | 0.594 | 1.000 | 0.247 | 0.708 | 0.522 | 1.000 | 0.012 | 0.035 | 0.974 | 1.000 | 0 | 0 | 0 | flat_flat |
| hsa-miR-1226 | -1.023 | -1.215 | -1.879 | -0.855 | 1.024 | 2.470 | 0.075 | 0.500 | -0.760 | -2.117 | 0.109 | 0.642 | 0.264 | 0.736 | 0.507 | 1.000 | 0 | 0 | 0 | flat_flat |
| hsa-miR-1226* | 5.051 | 6.300 | 8.748 | 8.406 | -0.341 | -0.444 | 0.682 | 1.000 | 3.072 | 4.610 | 0.013 | 0.159 | 2.730 | 4.098 | 0.018 | 0.180 | 0 | 0 | 0 | flat_flat |
| hsa-miR-1228* | 1.254 | 3.465 | -1.051 | 0.253 | 1.303 | 1.060 | 0.354 | 1.000 | -3.410 | -3.204 | 0.038 | 0.302 | -2.107 | -1.979 | 0.126 | 0.708 | 0 | 0 | 0 | flat_flat |
| hsa-miR-1229 | 1.388 | 0.940 | 2.139 | 3.248 | 1.109 | 2.380 | 0.083 | 0.536 | 0.975 | 2.415 | 0.080 | 0.515 | 2.084 | 5.163 | 0.009 | 0.105 | 0 | 0 | 0 | flat_flat |
| hsa-miR-1231 | -1.599 | -2.603 | -1.426 | -2.463 | -1.036 | -1.563 | 0.200 | 1.000 | 0.675 | 1.175 | 0.311 | 1.000 | -0.362 | -0.630 | 0.566 | 1.000 | 0 | 0 | 0 | flat_flat |
| hsa-miR-1233 | -1.484 | -1.311 | -1.481 | -0.448 | 1.033 | 2.504 | 0.073 | 0.485 | -0.083 | -0.232 | 0.829 | 1.000 | 0.950 | 2.660 | 0.063 | 0.426 | 0 | 0 | 0 | flat_flat |
| hsa-miR-1234 | 11.685 | 11.635 | 9.502 | 10.166 | 0.664 | 1.647 | 0.182 | 0.967 | -2.158 | -6.183 | 0.005 | 0.098 | -1.494 | -4.281 | 0.016 | 0.161 | 0 | 0 | 0 | flat_flat |
| hsa-miR-1236 | 0.716 | 1.229 | -0.036 | 0.858 | 0.893 | 1.846 | 0.146 | 0.826 | -1.008 | -2.406 | 0.081 | 0.517 | -0.115 | -0.274 | 0.799 | 1.000 | 0 | 0 | 0 | flat_flat |
| hsa-miR-124* | 0.485 | -0.509 | -1.559 | -1.912 | -0.354 | -0.537 | 0.622 | 1.000 | -1.547 | -2.710 | 0.060 | 0.420 | -1.901 | -3.330 | 0.034 | 0.279 | 0 | 0 | 0 | flat_flat |
| hsa-miR-1243 | -0.399 | 0.097 | -0.676 | -0.461 | 0.215 | 0.449 | 0.679 | 1.000 | -0.526 | -1.267 | 0.280 | 1.000 | -0.311 | -0.749 | 0.499 | 1.000 | 0 | 0 | 0 | flat_flat |
| hsa-miR-1244 | -1.086 | -0.489 | 0.030 | -0.337 | -0.367 | -0.720 | 0.515 | 1.000 | 0.818 | 1.852 | 0.145 | 0.781 | 0.450 | 1.020 | 0.371 | 1.000 | 0 | 0 | 0 | flat_flat |
| hsa-miR-1245 | -0.794 | -1.515 | -0.892 | -0.510 | 0.382 | 0.691 | 0.531 | 1.000 | 0.262 | 0.548 | 0.616 | 1.000 | 0.644 | 1.346 | 0.256 | 1.000 | 0 | 0 | 0 | flat_flat |
| hsa-miR-1246 | 8.198 | 9.344 | 11.142 | 9.797 | -1.345 | -1.858 | 0.144 | 0.819 | 2.372 | 3.783 | 0.023 | 0.234 | 1.026 | 1.637 | 0.184 | 0.954 | 0 | 0 | 0 | flat_flat |
| hsa-miR-1247 | -1.100 | -1.226 | -0.946 | -0.838 | 0.108 | 0.265 | 0.805 | 1.000 | 0.217 | 0.614 | 0.576 | 1.000 | 0.325 | 0.920 | 0.415 | 1.000 | 0 | 0 | 0 | flat_flat |
| hsa-miR-1248 | -2.041 | -1.897 | -1.475 | -1.623 | -0.148 | -0.361 | 0.738 | 1.000 | 0.494 | 1.392 | 0.243 | 1.000 | 0.346 | 0.975 | 0.390 | 1.000 | 0 | 0 | 0 | flat_flat |
| hsa-miR-1249 | 9.085 | 9.178 | 11.685 | 9.403 | -2.282 | -5.632 | 0.007 | 0.091 | 2.553 | 7.276 | 0.003 | 0.068 | 0.271 | 0.773 | 0.487 | 1.000 | 0 | 0 | 0 | flat_flat |
| hsa-miR-1250 | -0.817 | -0.719 | -0.468 | -0.790 | -0.322 | -0.795 | 0.476 | 1.000 | 0.300 | 0.854 | 0.446 | 1.000 | -0.022 | -0.063 | 0.953 | 1.000 | 0 | 0 | 0 | flat_flat |
| hsa-miR-1251 | -0.734 | -1.381 | -1.550 | -1.575 | -0.024 | -0.046 | 0.965 | 1.000 | -0.493 | -1.081 | 0.346 | 1.000 | -0.517 | -1.134 | 0.326 | 1.000 | 0 | 0 | 0 | flat_flat |
| hsa-miR-1252 | -1.302 | -1.188 | -1.352 | -1.492 | -0.140 | -0.344 | 0.750 | 1.000 | -0.107 | -0.305 | 0.777 | 1.000 | -0.247 | -0.702 | 0.525 | 1.000 | 0 | 0 | 0 | flat_flat |
| hsa-miR-1253 | -0.977 | -1.121 | -2.170 | -1.124 | 1.047 | 2.557 | 0.069 | 0.471 | -1.121 | -3.164 | 0.039 | 0.307 | -0.075 | -0.211 | 0.844 | 1.000 | 0 | 0 | 0 | flat_flat |
| hsa-miR-1254 | -1.258 | -0.836 | -0.901 | -1.117 | -0.216 | -0.470 | 0.665 | 1.000 | 0.146 | 0.368 | 0.733 | 1.000 | -0.070 | -0.175 | 0.870 | 1.000 | 0 | 0 | 0 | flat_flat |
| hsa-miR-1255a | -2.330 | -2.190 | -1.265 | -1.248 | 0.017 | 0.042 | 0.969 | 1.000 | 0.995 | 2.811 | 0.054 | 0.395 | 1.012 | 2.859 | 0.052 | 0.371 | 0 | 0 | 0 | flat_flat |
| hsa-miR-1255b | -1.891 | -2.177 | -1.403 | -1.832 | -0.428 | -0.998 | 0.380 | 1.000 | 0.631 | 1.696 | 0.172 | 0.884 | 0.202 | 0.544 | 0.618 | 1.000 | 0 | 0 | 0 | flat_flat |
| hsa-miR-1256 | -0.020 | 0.037 | -1.160 | -0.309 | 0.851 | 2.109 | 0.110 | 0.662 | -1.169 | -3.345 | 0.033 | 0.289 | -0.318 | -0.910 | 0.419 | 1.000 | 0 | 0 | 0 | flat_flat |
| hsa-miR-1257 | -0.863 | -0.817 | -0.225 | -0.504 | -0.280 | -0.694 | 0.530 | 1.000 | 0.616 | 1.764 | 0.160 | 0.830 | 0.336 | 0.963 | 0.395 | 1.000 | 0 | 0 | 0 | flat_flat |
| hsa-miR-1258 | 0.490 | 1.626 | -0.146 | -0.788 | -0.642 | -0.892 | 0.428 | 1.000 | -1.204 | -1.931 | 0.133 | 0.742 | -1.846 | -2.961 | 0.047 | 0.350 | 0 | 0 | 0 | flat_flat |
| hsa-miR-1259 | -1.336 | -1.546 | 0.416 | 0.070 | -0.346 | -0.830 | 0.458 | 1.000 | 1.857 | 5.142 | 0.009 | 0.131 | 1.511 | 4.184 | 0.017 | 0.170 | 0 | 0 | 0 | flat_flat |
| hsa-miR-125a-3p | 8.112 | 11.824 | 7.328 | 9.679 | 2.351 | 1.181 | 0.309 | 1.000 | -2.640 | -1.532 | 0.207 | 1.000 | -0.289 | -0.168 | 0.876 | 1.000 | 0 | 0 | 0 | flat_flat |
| hsa-miR-125a-5p | 9.344 | 8.890 | 11.603 | 10.340 | -1.263 | -2.702 | 0.060 | 0.427 | 2.486 | 6.140 | 0.005 | 0.098 | 1.223 | 3.020 | 0.044 | 0.338 | 0 | 0 | 0 | flat_flat |
| hsa-miR-125b | 9.178 | 9.998 | 10.340 | 10.959 | 0.620 | 1.052 | 0.358 | 1.000 | 0.752 | 1.474 | 0.222 | 1.000 | 1.371 | 2.689 | 0.061 | 0.415 | 0 | 0 | 0 | flat_flat |
| hsa-miR-125b-1* | -0.872 | -0.972 | -1.188 | -1.346 | -0.158 | -0.391 | 0.718 | 1.000 | -0.266 | -0.758 | 0.495 | 1.000 | -0.425 | -1.209 | 0.300 | 1.000 | 0 | 0 | 0 | flat_flat |
| hsa-miR-125b-2* | 0.536 | 0.622 | 0.006 | 2.670 | 2.664 | 6.581 | 0.004 | 0.063 | -0.573 | -1.634 | 0.185 | 0.933 | 2.091 | 5.965 | 0.005 | 0.074 | 0 | 0 | 0 | flat_flat |
| hsa-miR-1260 | 9.892 | 10.166 | 11.419 | 11.580 | 0.161 | 0.377 | 0.727 | 1.000 | 1.390 | 3.758 | 0.024 | 0.236 | 1.551 | 4.194 | 0.017 | 0.169 | 0 | 0 | 0 | flat_flat |
| hsa-miR-1261 | -0.027 | 0.044 | -0.335 | 0.217 | 0.552 | 1.366 | 0.251 | 1.000 | -0.344 | -0.982 | 0.387 | 1.000 | 0.208 | 0.595 | 0.587 | 1.000 | 0 | 0 | 0 | flat_flat |
| hsa-miR-1262 | -1.242 | -1.559 | 0.395 | -1.156 | -1.551 | -3.563 | 0.028 | 0.249 | 1.795 | 4.762 | 0.011 | 0.151 | 0.244 | 0.648 | 0.556 | 1.000 | 0 | 0 | 0 | flat_flat |
| hsa-miR-1263 | -0.777 | -0.684 | -0.060 | -0.416 | -0.357 | -0.880 | 0.433 | 1.000 | 0.671 | 1.911 | 0.136 | 0.747 | 0.314 | 0.895 | 0.426 | 1.000 | 0 | 0 | 0 | flat_flat |
| hsa-miR-1264 | -0.293 | -0.049 | -0.368 | 0.286 | 0.655 | 1.550 | 0.203 | 1.000 | -0.197 | -0.540 | 0.621 | 1.000 | 0.457 | 1.251 | 0.286 | 1.000 | 0 | 0 | 0 | flat_flat |
| hsa-miR-1265 | -1.180 | -0.478 | 0.683 | -0.814 | -1.497 | -2.745 | 0.058 | 0.420 | 1.512 | 3.202 | 0.038 | 0.302 | 0.015 | 0.032 | 0.976 | 1.000 | 0 | 0 | 0 | flat_flat |
| hsa-miR-1266 | -1.540 | -2.041 | -1.049 | -1.405 | -0.356 | -0.742 | 0.503 | 1.000 | 0.742 | 1.782 | 0.157 | 0.821 | 0.385 | 0.926 | 0.412 | 1.000 | 0 | 0 | 0 | flat_flat |
| hsa-miR-1267 | 0.845 | 0.845 | 1.582 | 2.894 | 1.312 | 3.261 | 0.036 | 0.299 | 0.737 | 2.117 | 0.109 | 0.642 | 2.049 | 5.883 | 0.006 | 0.077 | 0 | 0 | 0 | flat_flat |
| hsa-miR-1268 | 13.681 | 13.653 | 13.482 | 12.407 | -1.075 | -2.671 | 0.062 | 0.431 | -0.185 | -0.530 | 0.627 | 1.000 | -1.260 | -3.614 | 0.027 | 0.236 | 0 | 0 | 0 | flat_flat |
| hsa-miR-1269 | -1.202 | -1.263 | -1.023 | -1.013 | 0.011 | 0.026 | 0.980 | 1.000 | 0.209 | 0.598 | 0.585 | 1.000 | 0.220 | 0.629 | 0.567 | 1.000 | 0 | 0 | 0 | flat_flat |
| hsa-miR-127-5p | -2.444 | -1.504 | -0.238 | -2.170 | -1.932 | -3.034 | 0.044 | 0.346 | 1.736 | 3.148 | 0.040 | 0.309 | -0.196 | -0.356 | 0.742 | 1.000 | 0 | 0 | 0 | flat_flat |
| hsa-miR-1270 | -0.583 | -0.615 | 0.898 | -1.323 | -2.222 | -5.519 | 0.007 | 0.095 | 1.497 | 4.294 | 0.016 | 0.182 | -0.725 | -2.079 | 0.113 | 0.662 | 0 | 0 | 0 | flat_flat |
| hsa-miR-1271 | 2.368 | 2.196 | 2.068 | 3.401 | 1.332 | 3.232 | 0.037 | 0.304 | -0.214 | -0.598 | 0.585 | 1.000 | 1.119 | 3.134 | 0.040 | 0.315 | 0 | 0 | 0 | flat_flat |
| hsa-miR-1272 | -1.465 | -0.572 | 0.917 | -0.901 | -1.818 | -2.941 | 0.048 | 0.368 | 1.936 | 3.616 | 0.027 | 0.251 | 0.118 | 0.220 | 0.838 | 1.000 | 0 | 0 | 0 | flat_flat |
| hsa-miR-1273 | -1.546 | -1.185 | -1.529 | -0.657 | 0.873 | 1.962 | 0.129 | 0.752 | -0.163 | -0.424 | 0.696 | 1.000 | 0.709 | 1.842 | 0.147 | 0.790 | 0 | 0 | 0 | flat_flat |
| hsa-miR-1274a | 12.142 | 12.055 | 12.727 | 12.807 | 0.080 | 0.198 | 0.854 | 1.000 | 0.629 | 1.793 | 0.155 | 0.816 | 0.709 | 2.021 | 0.121 | 0.689 | 0 | 0 | 0 | flat_flat |
| hsa-miR-1274b | 13.432 | 13.472 | 13.618 | 13.794 | 0.176 | 0.437 | 0.687 | 1.000 | 0.166 | 0.476 | 0.661 | 1.000 | 0.342 | 0.981 | 0.387 | 1.000 | 0 | 0 | 0 | flat_flat |
| hsa-miR-1275 | 10.959 | 11.142 | 11.370 | 12.976 | 1.606 | 3.883 | 0.021 | 0.206 | 0.319 | 0.890 | 0.429 | 1.000 | 1.925 | 5.374 | 0.008 | 0.095 | 0 | 0 | 0 | flat_flat |
| hsa-miR-1276 | -0.814 | -0.461 | -1.092 | -0.399 | 0.694 | 1.567 | 0.199 | 1.000 | -0.455 | -1.186 | 0.308 | 1.000 | 0.239 | 0.624 | 0.570 | 1.000 | 0 | 0 | 0 | flat_flat |
| hsa-miR-1277 | -0.919 | -0.838 | 0.228 | 0.395 | 0.166 | 0.411 | 0.704 | 1.000 | 1.107 | 3.160 | 0.039 | 0.307 | 1.273 | 3.634 | 0.026 | 0.234 | 0 | 0 | 0 | flat_flat |
| hsa-miR-1278 | -0.710 | -0.591 | -0.180 | -0.228 | -0.048 | -0.118 | 0.912 | 1.000 | 0.471 | 1.336 | 0.259 | 1.000 | 0.423 | 1.199 | 0.303 | 1.000 | 0 | 0 | 0 | flat_flat |
| hsa-miR-1279 | -0.352 | -0.416 | 0.054 | -0.027 | -0.081 | -0.202 | 0.851 | 1.000 | 0.438 | 1.253 | 0.285 | 1.000 | 0.357 | 1.020 | 0.371 | 1.000 | 0 | 0 | 0 | flat_flat |
| hsa-miR-1280 | 10.431 | 10.517 | 10.798 | 12.395 | 1.597 | 3.945 | 0.020 | 0.200 | 0.324 | 0.925 | 0.412 | 1.000 | 1.921 | 5.480 | 0.007 | 0.090 | 0 | 0 | 0 | flat_flat |
| hsa-miR-1281 | 7.881 | 9.863 | 5.734 | 9.279 | 3.545 | 3.176 | 0.039 | 0.314 | -3.138 | -3.247 | 0.036 | 0.300 | 0.407 | 0.421 | 0.698 | 1.000 | 0 | 0 | 0 | flat_flat |
| hsa-miR-1282 | 0.103 | -0.180 | -0.521 | 0.664 | 1.185 | 2.764 | 0.057 | 0.417 | -0.483 | -1.300 | 0.270 | 1.000 | 0.702 | 1.891 | 0.139 | 0.759 | 0 | 0 | 0 | flat_flat |
| hsa-miR-1283 | -1.667 | -2.483 | -2.150 | -2.398 | -0.248 | -0.422 | 0.697 | 1.000 | -0.075 | -0.147 | 0.891 | 1.000 | -0.323 | -0.635 | 0.563 | 1.000 | 0 | 0 | 0 | flat_flat |
| hsa-miR-1284 | -0.066 | -0.376 | -1.654 | 0.134 | 1.789 | 4.121 | 0.018 | 0.181 | -1.434 | -3.814 | 0.023 | 0.229 | 0.355 | 0.945 | 0.403 | 1.000 | 0 | 0 | 0 | flat_flat |
| hsa-miR-1285 | -0.588 | -0.545 | -1.274 | 0.126 | 1.400 | 3.476 | 0.030 | 0.262 | -0.708 | -2.028 | 0.120 | 0.688 | 0.693 | 1.985 | 0.125 | 0.705 | 0 | 0 | 0 | flat_flat |
| hsa-miR-1286 | -0.521 | -0.208 | -1.449 | -1.023 | 0.426 | 0.980 | 0.388 | 1.000 | -1.085 | -2.883 | 0.050 | 0.377 | -0.659 | -1.751 | 0.162 | 0.856 | 0 | 0 | 0 | flat_flat |
| hsa-miR-1287 | -0.761 | 0.054 | 0.867 | 0.251 | -0.616 | -1.049 | 0.359 | 1.000 | 1.220 | 2.400 | 0.081 | 0.517 | 0.604 | 1.188 | 0.307 | 1.000 | 0 | 0 | 0 | flat_flat |
| hsa-miR-1288 | 9.279 | 12.269 | 2.735 | 11.142 | 8.408 | 5.188 | 0.009 | 0.110 | -8.039 | -5.727 | 0.006 | 0.108 | 0.369 | 0.263 | 0.807 | 1.000 | 0 | 0 | 0 | flat_flat |
| hsa-miR-1289 | -0.882 | -0.744 | -0.131 | -0.323 | -0.192 | -0.470 | 0.665 | 1.000 | 0.683 | 1.928 | 0.133 | 0.742 | 0.491 | 1.386 | 0.245 | 1.000 | 0 | 0 | 0 | flat_flat |
| hsa-miR-129-3p | 6.080 | 5.894 | 3.649 | 3.702 | 0.053 | 0.129 | 0.904 | 1.000 | -2.338 | -6.523 | 0.004 | 0.087 | -2.285 | -6.374 | 0.004 | 0.061 | 0 | 0 | 0 | flat_flat |
| hsa-miR-129-5p | 0.950 | 0.569 | 1.070 | -0.368 | -1.438 | -3.202 | 0.038 | 0.309 | 0.310 | 0.798 | 0.474 | 1.000 | -1.128 | -2.899 | 0.050 | 0.367 | 0 | 0 | 0 | flat_flat |
| hsa-miR-1290 | 7.270 | 7.707 | 9.468 | 6.501 | -2.966 | -6.404 | 0.004 | 0.066 | 1.979 | 4.934 | 0.010 | 0.143 | -0.987 | -2.461 | 0.076 | 0.497 | 0 | 0 | 0 | flat_flat |
| hsa-miR-1291 | -0.951 | -1.465 | -1.683 | -1.515 | 0.168 | 0.347 | 0.748 | 1.000 | -0.475 | -1.132 | 0.327 | 1.000 | -0.307 | -0.731 | 0.509 | 1.000 | 0 | 0 | 0 | flat_flat |
| hsa-miR-1292 | -0.494 | -0.986 | -1.156 | -2.031 | -0.875 | -1.830 | 0.149 | 0.834 | -0.416 | -1.004 | 0.377 | 1.000 | -1.290 | -3.117 | 0.041 | 0.317 | 0 | 0 | 0 | flat_flat |
| hsa-miR-1293 | -1.387 | -0.765 | -0.817 | -0.882 | -0.065 | -0.125 | 0.907 | 1.000 | 0.259 | 0.577 | 0.598 | 1.000 | 0.194 | 0.432 | 0.690 | 1.000 | 0 | 0 | 0 | flat_flat |
| hsa-miR-1294 | -0.246 | -0.053 | 1.073 | -0.087 | -1.160 | -2.796 | 0.055 | 0.407 | 1.222 | 3.403 | 0.032 | 0.283 | 0.063 | 0.174 | 0.871 | 1.000 | 0 | 0 | 0 | flat_flat |
| hsa-miR-1295 | -0.475 | 1.600 | 4.747 | 1.635 | -3.112 | -2.679 | 0.061 | 0.430 | 4.185 | 4.160 | 0.017 | 0.195 | 1.073 | 1.066 | 0.352 | 1.000 | 0 | 0 | 0 | flat_flat |
| hsa-miR-1296 | 1.313 | 0.985 | 0.875 | 0.692 | -0.183 | -0.419 | 0.699 | 1.000 | -0.274 | -0.722 | 0.514 | 1.000 | -0.457 | -1.206 | 0.301 | 1.000 | 0 | 0 | 0 | flat_flat |
| hsa-miR-1297 | -2.140 | -1.752 | -1.114 | -1.223 | -0.109 | -0.242 | 0.822 | 1.000 | 0.831 | 2.129 | 0.107 | 0.636 | 0.722 | 1.849 | 0.145 | 0.785 | 0 | 0 | 0 | flat_flat |
| hsa-miR-1298 | -0.180 | 0.084 | -2.099 | -1.064 | 1.036 | 2.435 | 0.078 | 0.516 | -2.051 | -5.568 | 0.007 | 0.112 | -1.015 | -2.756 | 0.057 | 0.397 | 0 | 0 | 0 | flat_flat |
| hsa-miR-1299 | 0.972 | 1.926 | 2.039 | 1.161 | -0.878 | -1.367 | 0.250 | 1.000 | 0.590 | 1.061 | 0.354 | 1.000 | -0.288 | -0.517 | 0.635 | 1.000 | 0 | 0 | 0 | flat_flat |
| hsa-miR-1300 | 2.123 | 1.496 | 2.390 | 0.350 | -2.040 | -3.924 | 0.021 | 0.203 | 0.580 | 1.288 | 0.274 | 1.000 | -1.460 | -3.243 | 0.036 | 0.290 | 0 | 0 | 0 | flat_flat |
| hsa-miR-1301 | -1.035 | -1.202 | 0.823 | -0.941 | -1.763 | -4.284 | 0.016 | 0.169 | 1.941 | 5.445 | 0.007 | 0.117 | 0.178 | 0.499 | 0.647 | 1.000 | 0 | 0 | 0 | flat_flat |
| hsa-miR-1302 | -1.381 | -1.445 | -0.204 | -0.632 | -0.428 | -1.060 | 0.355 | 1.000 | 1.209 | 3.458 | 0.030 | 0.276 | 0.781 | 2.234 | 0.096 | 0.589 | 0 | 0 | 0 | flat_flat |
| hsa-miR-1303 | -0.106 | -0.100 | -0.631 | 0.898 | 1.529 | 3.802 | 0.023 | 0.216 | -0.528 | -1.515 | 0.211 | 1.000 | 1.001 | 2.875 | 0.051 | 0.370 | 0 | 0 | 0 | flat_flat |
| hsa-miR-1304 | -1.077 | -1.800 | -0.392 | -1.183 | -0.791 | -1.430 | 0.233 | 1.000 | 1.046 | 2.184 | 0.101 | 0.607 | 0.255 | 0.533 | 0.625 | 1.000 | 0 | 0 | 0 | flat_flat |
| hsa-miR-1307 | -0.053 | -0.197 | 0.403 | -0.787 | -1.190 | -2.908 | 0.049 | 0.372 | 0.528 | 1.491 | 0.217 | 1.000 | -0.662 | -1.867 | 0.143 | 0.773 | 0 | 0 | 0 | flat_flat |
| hsa-miR-1308 | 11.657 | 11.544 | 12.341 | 11.419 | -0.922 | -2.269 | 0.093 | 0.580 | 0.741 | 2.104 | 0.110 | 0.647 | -0.182 | -0.516 | 0.636 | 1.000 | 0 | 0 | 0 | flat_flat |
| hsa-miR-130a | 13.961 | 13.894 | 13.926 | 13.894 | -0.033 | -0.081 | 0.940 | 1.000 | -0.001 | -0.004 | 0.997 | 1.000 | -0.034 | -0.097 | 0.928 | 1.000 | 0 | 0 | 0 | flat_flat |
| hsa-miR-130a* | 0.762 | 0.258 | 0.115 | 1.534 | 1.419 | 2.948 | 0.048 | 0.366 | -0.395 | -0.947 | 0.402 | 1.000 | 1.024 | 2.457 | 0.076 | 0.498 | 0 | 0 | 0 | flat_flat |
| hsa-miR-130b | 13.410 | 13.272 | 13.410 | 12.702 | -0.708 | -1.732 | 0.166 | 0.901 | 0.069 | 0.195 | 0.855 | 1.000 | -0.639 | -1.804 | 0.153 | 0.815 | 0 | 0 | 0 | flat_flat |
| hsa-miR-130b* | 0.416 | 0.269 | 1.012 | 1.967 | 0.956 | 2.333 | 0.087 | 0.554 | 0.669 | 1.887 | 0.140 | 0.756 | 1.625 | 4.581 | 0.013 | 0.141 | 0 | 0 | 0 | flat_flat |
| hsa-miR-1321 | -1.286 | -0.905 | 0.222 | -1.625 | -1.847 | -4.112 | 0.018 | 0.182 | 1.318 | 3.387 | 0.032 | 0.284 | -0.529 | -1.361 | 0.252 | 1.000 | 0 | 0 | 0 | flat_flat |
| hsa-miR-1322 | -0.926 | -0.916 | -0.664 | -0.965 | -0.300 | -0.747 | 0.501 | 1.000 | 0.257 | 0.737 | 0.506 | 1.000 | -0.044 | -0.126 | 0.907 | 1.000 | 0 | 0 | 0 | flat_flat |
| hsa-miR-1324 | -0.765 | -0.823 | -0.448 | -0.451 | -0.003 | -0.007 | 0.994 | 1.000 | 0.346 | 0.991 | 0.383 | 1.000 | 0.343 | 0.983 | 0.387 | 1.000 | 0 | 0 | 0 | flat_flat |
| hsa-miR-133a | 0.608 | 0.733 | -0.267 | 1.830 | 2.097 | 5.146 | 0.009 | 0.113 | -0.938 | -2.658 | 0.063 | 0.437 | 1.159 | 3.285 | 0.035 | 0.282 | 0 | 0 | 0 | flat_flat |
| hsa-miR-133b | 1.025 | 0.847 | 0.622 | 3.381 | 2.759 | 6.681 | 0.004 | 0.061 | -0.314 | -0.877 | 0.435 | 1.000 | 2.445 | 6.837 | 0.003 | 0.053 | 0 | 0 | 0 | flat_flat |
| hsa-miR-135a | 1.327 | 1.489 | 1.077 | 1.646 | 0.569 | 1.385 | 0.245 | 1.000 | -0.331 | -0.930 | 0.410 | 1.000 | 0.238 | 0.669 | 0.544 | 1.000 | 0 | 0 | 0 | flat_flat |
| hsa-miR-135a* | 12.111 | 9.279 | 8.011 | 4.860 | -3.151 | -2.046 | 0.117 | 0.699 | -2.684 | -2.012 | 0.122 | 0.694 | -5.835 | -4.374 | 0.015 | 0.157 | 0 | 0 | 0 | flat_flat |
| hsa-miR-135b* | -1.877 | -2.747 | -2.765 | -3.098 | -0.333 | -0.547 | 0.616 | 1.000 | -0.453 | -0.859 | 0.443 | 1.000 | -0.786 | -1.491 | 0.217 | 1.000 | 0 | 0 | 0 | flat_flat |
| hsa-miR-136 | 0.428 | 0.325 | -0.794 | 1.683 | 2.477 | 6.104 | 0.005 | 0.076 | -1.170 | -3.329 | 0.034 | 0.289 | 1.307 | 3.719 | 0.024 | 0.223 | 0 | 0 | 0 | flat_flat |
| hsa-miR-136* | -0.854 | -0.600 | -1.003 | -0.531 | 0.472 | 1.114 | 0.334 | 1.000 | -0.276 | -0.752 | 0.498 | 1.000 | 0.196 | 0.534 | 0.624 | 1.000 | 0 | 0 | 0 | flat_flat |
| hsa-miR-137 | -0.696 | -0.345 | -1.134 | -0.872 | 0.261 | 0.591 | 0.589 | 1.000 | -0.613 | -1.601 | 0.192 | 0.959 | -0.352 | -0.919 | 0.415 | 1.000 | 0 | 0 | 0 | flat_flat |
| hsa-miR-138 | -0.877 | -1.423 | -0.421 | -1.364 | -0.942 | -1.908 | 0.136 | 0.783 | 0.729 | 1.703 | 0.171 | 0.881 | -0.214 | -0.500 | 0.646 | 1.000 | 0 | 0 | 0 | flat_flat |
| hsa-miR-138-1* | -0.182 | 0.213 | -2.216 | -2.238 | -0.022 | -0.049 | 0.964 | 1.000 | -2.232 | -5.696 | 0.006 | 0.109 | -2.254 | -5.752 | 0.006 | 0.081 | 0 | 0 | 0 | flat_flat |
| hsa-miR-139-3p | -0.253 | 4.246 | 1.943 | 1.070 | -0.873 | -0.364 | 0.736 | 1.000 | -0.053 | -0.026 | 0.981 | 1.000 | -0.926 | -0.446 | 0.681 | 1.000 | 0 | 0 | 0 | flat_flat |
| hsa-miR-140-5p | 8.820 | 7.453 | 9.797 | 11.544 | 1.747 | 2.123 | 0.108 | 0.656 | 1.661 | 2.331 | 0.087 | 0.542 | 3.408 | 4.782 | 0.011 | 0.128 | 0 | 0 | 0 | flat_flat |
| hsa-miR-141* | -0.787 | -1.274 | -0.504 | -0.764 | -0.260 | -0.545 | 0.617 | 1.000 | 0.526 | 1.274 | 0.278 | 1.000 | 0.266 | 0.645 | 0.558 | 1.000 | 0 | 0 | 0 | flat_flat |
| hsa-miR-143 | 0.960 | -0.255 | -0.302 | -0.990 | -0.688 | -0.912 | 0.418 | 1.000 | -0.654 | -1.001 | 0.379 | 1.000 | -1.343 | -2.055 | 0.116 | 0.675 | 0 | 0 | 0 | flat_flat |
| hsa-miR-143* | -0.509 | -1.268 | -0.020 | -0.317 | -0.297 | -0.524 | 0.631 | 1.000 | 0.869 | 1.771 | 0.159 | 0.827 | 0.572 | 1.166 | 0.314 | 1.000 | 0 | 0 | 0 | flat_flat |
| hsa-miR-144 | -1.445 | -1.475 | -0.489 | -0.545 | -0.056 | -0.138 | 0.897 | 1.000 | 0.971 | 2.785 | 0.055 | 0.401 | 0.915 | 2.625 | 0.065 | 0.439 | 0 | 0 | 0 | flat_flat |
| hsa-miR-144* | -0.896 | -0.652 | -1.342 | -0.570 | 0.772 | 1.829 | 0.149 | 0.834 | -0.568 | -1.553 | 0.203 | 0.999 | 0.204 | 0.559 | 0.609 | 1.000 | 0 | 0 | 0 | flat_flat |
| hsa-miR-145* | -0.204 | 0.103 | -0.352 | -0.543 | -0.191 | -0.442 | 0.684 | 1.000 | -0.301 | -0.802 | 0.472 | 1.000 | -0.492 | -1.312 | 0.266 | 1.000 | 0 | 0 | 0 | flat_flat |
| hsa-miR-1468 | -1.357 | -0.788 | -1.302 | -0.877 | 0.426 | 0.849 | 0.448 | 1.000 | -0.230 | -0.530 | 0.627 | 1.000 | 0.196 | 0.451 | 0.678 | 1.000 | 0 | 0 | 0 | flat_flat |
| hsa-miR-1469 | -0.451 | -0.015 | -0.845 | -1.403 | -0.558 | -1.205 | 0.301 | 1.000 | -0.613 | -1.528 | 0.208 | 1.000 | -1.170 | -2.920 | 0.049 | 0.362 | 0 | 0 | 0 | flat_flat |
| hsa-miR-146a* | -0.409 | -0.704 | -1.258 | 0.613 | 1.872 | 4.341 | 0.015 | 0.163 | -0.702 | -1.879 | 0.141 | 0.761 | 1.170 | 3.133 | 0.040 | 0.315 | 0 | 0 | 0 | flat_flat |
| hsa-miR-146b-3p | -1.766 | -2.081 | 0.113 | -1.142 | -1.255 | -2.885 | 0.050 | 0.379 | 2.036 | 5.407 | 0.007 | 0.118 | 0.782 | 2.075 | 0.114 | 0.663 | 0 | 0 | 0 | flat_flat |
| hsa-miR-147 | -1.013 | -0.574 | -1.268 | -1.514 | -0.246 | -0.531 | 0.626 | 1.000 | -0.475 | -1.183 | 0.309 | 1.000 | -0.721 | -1.796 | 0.154 | 0.819 | 0 | 0 | 0 | flat_flat |
| hsa-miR-147b | -2.082 | -1.912 | -0.941 | -2.313 | -1.372 | -3.331 | 0.034 | 0.285 | 1.057 | 2.961 | 0.047 | 0.357 | -0.316 | -0.885 | 0.431 | 1.000 | 0 | 0 | 0 | flat_flat |
| hsa-miR-148a* | -1.248 | -0.924 | -0.637 | -1.121 | -0.484 | -1.108 | 0.336 | 1.000 | 0.449 | 1.186 | 0.307 | 1.000 | -0.035 | -0.094 | 0.930 | 1.000 | 0 | 0 | 0 | flat_flat |
| hsa-miR-148b* | -0.335 | -1.683 | -0.309 | 0.365 | 0.675 | 0.829 | 0.458 | 1.000 | 0.700 | 0.992 | 0.383 | 1.000 | 1.375 | 1.949 | 0.130 | 0.728 | 0 | 0 | 0 | flat_flat |
| hsa-miR-149 | 5.142 | 4.358 | 6.794 | 5.549 | -1.245 | -2.163 | 0.104 | 0.635 | 2.044 | 4.100 | 0.018 | 0.200 | 0.799 | 1.603 | 0.192 | 0.974 | 0 | 0 | 0 | flat_flat |
| hsa-miR-149* | -0.285 | -0.494 | 0.213 | -2.136 | -2.349 | -5.633 | 0.007 | 0.091 | 0.603 | 1.669 | 0.178 | 0.901 | -1.746 | -4.835 | 0.011 | 0.125 | 0 | 0 | 0 | flat_flat |
| hsa-miR-151-5p | 11.299 | 10.959 | 12.152 | 10.676 | -1.475 | -3.354 | 0.033 | 0.281 | 1.023 | 2.685 | 0.061 | 0.428 | -0.453 | -1.188 | 0.307 | 1.000 | 0 | 0 | 0 | flat_flat |
| hsa-miR-152 | -0.757 | 0.209 | -0.972 | -1.069 | -0.097 | -0.150 | 0.889 | 1.000 | -0.698 | -1.245 | 0.288 | 1.000 | -0.795 | -1.418 | 0.236 | 1.000 | 0 | 0 | 0 | flat_flat |
| hsa-miR-1537 | -0.744 | -1.527 | -0.858 | -0.285 | 0.573 | 0.997 | 0.381 | 1.000 | 0.277 | 0.557 | 0.610 | 1.000 | 0.851 | 1.708 | 0.170 | 0.892 | 0 | 0 | 0 | flat_flat |
| hsa-miR-1538 | -0.041 | -0.163 | -0.251 | 0.413 | 0.665 | 1.631 | 0.185 | 0.982 | -0.150 | -0.424 | 0.695 | 1.000 | 0.515 | 1.460 | 0.225 | 1.000 | 0 | 0 | 0 | flat_flat |
| hsa-miR-154 | 0.109 | 0.683 | 1.035 | 1.327 | 0.292 | 0.581 | 0.595 | 1.000 | 0.639 | 1.467 | 0.223 | 1.000 | 0.931 | 2.139 | 0.106 | 0.642 | 0 | 0 | 0 | flat_flat |
| hsa-miR-154* | -0.695 | -0.676 | -1.035 | 0.733 | 1.768 | 4.395 | 0.015 | 0.161 | -0.350 | -1.003 | 0.378 | 1.000 | 1.419 | 4.071 | 0.019 | 0.181 | 0 | 0 | 0 | flat_flat |
| hsa-miR-155* | -0.858 | -1.331 | -0.950 | 1.545 | 2.495 | 5.277 | 0.008 | 0.106 | 0.145 | 0.353 | 0.743 | 1.000 | 2.639 | 6.447 | 0.004 | 0.061 | 0 | 0 | 0 | flat_flat |
| hsa-miR-15a | 13.025 | 12.638 | 13.172 | 13.472 | 0.300 | 0.665 | 0.546 | 1.000 | 0.340 | 0.872 | 0.437 | 1.000 | 0.640 | 1.640 | 0.184 | 0.954 | 0 | 0 | 0 | flat_flat |
| hsa-miR-15a* | -0.012 | -0.002 | 0.105 | 2.368 | 2.263 | 5.624 | 0.007 | 0.091 | 0.112 | 0.322 | 0.765 | 1.000 | 2.375 | 6.816 | 0.003 | 0.053 | 0 | 0 | 0 | flat_flat |
| hsa-miR-15b | 13.794 | 13.794 | 13.869 | 13.482 | -0.387 | -0.962 | 0.396 | 1.000 | 0.075 | 0.215 | 0.841 | 1.000 | -0.312 | -0.896 | 0.426 | 1.000 | 0 | 0 | 0 | flat_flat |
| hsa-miR-15b* | -0.009 | 0.523 | 0.017 | 1.254 | 1.236 | 2.524 | 0.071 | 0.477 | -0.240 | -0.565 | 0.605 | 1.000 | 0.997 | 2.350 | 0.085 | 0.537 | 0 | 0 | 0 | flat_flat |
| hsa-miR-16 | 12.507 | 12.469 | 13.006 | 12.243 | -0.762 | -1.893 | 0.139 | 0.793 | 0.518 | 1.484 | 0.219 | 1.000 | -0.245 | -0.702 | 0.525 | 1.000 | 0 | 0 | 0 | flat_flat |
| hsa-miR-16-1* | -1.283 | -1.514 | -0.919 | -0.699 | 0.220 | 0.523 | 0.631 | 1.000 | 0.480 | 1.319 | 0.264 | 1.000 | 0.700 | 1.923 | 0.134 | 0.741 | 0 | 0 | 0 | flat_flat |
| hsa-miR-17 | 12.902 | 12.727 | 13.544 | 13.172 | -0.372 | -0.902 | 0.423 | 1.000 | 0.729 | 2.041 | 0.118 | 0.682 | 0.357 | 1.000 | 0.379 | 1.000 | 0 | 0 | 0 | flat_flat |
| hsa-miR-17* | 8.620 | 8.550 | 7.219 | 9.767 | 2.548 | 6.308 | 0.004 | 0.069 | -1.366 | -3.905 | 0.021 | 0.220 | 1.182 | 3.379 | 0.032 | 0.271 | 0 | 0 | 0 | flat_flat |
| hsa-miR-181c* | 3.843 | 7.531 | -0.772 | 7.881 | 8.653 | 4.374 | 0.015 | 0.161 | -6.459 | -3.770 | 0.023 | 0.235 | 2.194 | 1.281 | 0.276 | 1.000 | 0 | 0 | 0 | flat_flat |
| hsa-miR-181d | 5.201 | 6.464 | 5.549 | 9.962 | 4.413 | 5.688 | 0.006 | 0.090 | -0.284 | -0.422 | 0.697 | 1.000 | 4.129 | 6.146 | 0.005 | 0.069 | 0 | 0 | 0 | flat_flat |
| hsa-miR-182 | 0.683 | -0.246 | -0.963 | -1.298 | -0.335 | -0.530 | 0.627 | 1.000 | -1.182 | -2.158 | 0.104 | 0.620 | -1.517 | -2.769 | 0.056 | 0.395 | 0 | 0 | 0 | flat_flat |
| hsa-miR-182* | -0.729 | -1.342 | -1.360 | 0.033 | 1.393 | 2.705 | 0.060 | 0.427 | -0.325 | -0.728 | 0.511 | 1.000 | 1.068 | 2.395 | 0.081 | 0.518 | 0 | 0 | 0 | flat_flat |
| hsa-miR-1825 | 6.669 | 7.856 | 4.609 | 8.364 | 3.755 | 5.062 | 0.009 | 0.117 | -2.653 | -4.131 | 0.018 | 0.198 | 1.101 | 1.714 | 0.169 | 0.889 | 0 | 0 | 0 | flat_flat |
| hsa-miR-1827 | -2.031 | -2.398 | -0.143 | -1.399 | -1.256 | -2.817 | 0.054 | 0.400 | 2.072 | 5.363 | 0.008 | 0.120 | 0.815 | 2.111 | 0.110 | 0.653 | 0 | 0 | 0 | flat_flat |
| hsa-miR-183* | -1.515 | -1.500 | -2.204 | -2.599 | -0.395 | -0.982 | 0.387 | 1.000 | -0.697 | -1.999 | 0.123 | 0.702 | -1.091 | -3.133 | 0.040 | 0.315 | 0 | 0 | 0 | flat_flat |
| hsa-miR-184 | 2.583 | 1.524 | 1.367 | -1.307 | -2.674 | -3.898 | 0.021 | 0.205 | -0.686 | -1.155 | 0.318 | 1.000 | -3.361 | -5.656 | 0.006 | 0.085 | 0 | 0 | 0 | flat_flat |
| hsa-miR-185* | -0.392 | 0.139 | -0.549 | 0.544 | 1.092 | 2.232 | 0.096 | 0.599 | -0.422 | -0.996 | 0.381 | 1.000 | 0.670 | 1.581 | 0.196 | 0.986 | 0 | 0 | 0 | flat_flat |
| hsa-miR-186* | 0.163 | -0.166 | 1.744 | -0.556 | -2.299 | -5.253 | 0.008 | 0.106 | 1.746 | 4.604 | 0.013 | 0.159 | -0.554 | -1.461 | 0.225 | 1.000 | 0 | 0 | 0 | flat_flat |
| hsa-miR-187 | -1.346 | -0.879 | -1.752 | -2.947 | -1.195 | -2.536 | 0.071 | 0.474 | -0.639 | -1.565 | 0.200 | 0.986 | -1.834 | -4.494 | 0.014 | 0.146 | 0 | 0 | 0 | flat_flat |
| hsa-miR-188-3p | -1.550 | -2.170 | -0.924 | -1.840 | -0.916 | -1.769 | 0.159 | 0.878 | 0.936 | 2.089 | 0.112 | 0.655 | 0.020 | 0.046 | 0.966 | 1.000 | 0 | 0 | 0 | flat_flat |
| hsa-miR-188-5p | 9.012 | 10.560 | 10.267 | 6.119 | -4.148 | -4.573 | 0.013 | 0.148 | 0.481 | 0.612 | 0.577 | 1.000 | -3.667 | -4.669 | 0.012 | 0.135 | 0 | 0 | 0 | flat_flat |
| hsa-miR-18a | 12.284 | 12.067 | 12.872 | 10.889 | -1.983 | -4.743 | 0.011 | 0.137 | 0.696 | 1.924 | 0.134 | 0.742 | -1.286 | -3.553 | 0.028 | 0.246 | 0 | 0 | 0 | flat_flat |
| hsa-miR-18a* | -0.100 | -0.549 | -0.905 | -0.382 | 0.523 | 1.122 | 0.330 | 1.000 | -0.580 | -1.438 | 0.231 | 1.000 | -0.057 | -0.142 | 0.895 | 1.000 | 0 | 0 | 0 | flat_flat |
| hsa-miR-18b* | 1.435 | 1.388 | 0.940 | 2.818 | 1.878 | 4.661 | 0.012 | 0.143 | -0.472 | -1.353 | 0.254 | 1.000 | 1.406 | 4.030 | 0.019 | 0.185 | 0 | 0 | 0 | flat_flat |
| hsa-miR-190 | -1.434 | -1.283 | -0.197 | -0.392 | -0.195 | -0.477 | 0.661 | 1.000 | 1.162 | 3.272 | 0.035 | 0.298 | 0.966 | 2.722 | 0.059 | 0.406 | 0 | 0 | 0 | flat_flat |
| hsa-miR-1908 | -0.570 | -1.134 | -1.016 | -1.443 | -0.427 | -0.855 | 0.445 | 1.000 | -0.164 | -0.379 | 0.726 | 1.000 | -0.591 | -1.366 | 0.251 | 1.000 | 0 | 0 | 0 | flat_flat |
| hsa-miR-1909 | -1.814 | -1.733 | -2.082 | -0.977 | 1.105 | 2.733 | 0.058 | 0.420 | -0.309 | -0.881 | 0.433 | 1.000 | 0.797 | 2.274 | 0.092 | 0.573 | 0 | 0 | 0 | flat_flat |
| hsa-miR-1909* | 1.077 | 1.478 | -0.657 | -0.250 | 0.406 | 0.895 | 0.426 | 1.000 | -1.934 | -4.918 | 0.010 | 0.144 | -1.528 | -3.885 | 0.021 | 0.199 | 0 | 0 | 0 | flat_flat |
| hsa-miR-190b | -1.443 | -1.519 | 0.478 | -0.424 | -0.902 | -2.231 | 0.096 | 0.599 | 1.959 | 5.596 | 0.007 | 0.112 | 1.057 | 3.020 | 0.044 | 0.338 | 0 | 0 | 0 | flat_flat |
| hsa-miR-191 | -0.959 | 0.188 | -2.276 | 1.173 | 3.449 | 4.761 | 0.011 | 0.136 | -1.891 | -3.014 | 0.045 | 0.344 | 1.558 | 2.484 | 0.074 | 0.489 | 0 | 0 | 0 | flat_flat |
| hsa-miR-1910 | 1.248 | 1.161 | -0.630 | -0.306 | 0.324 | 0.799 | 0.473 | 1.000 | -1.834 | -5.232 | 0.008 | 0.125 | -1.511 | -4.309 | 0.016 | 0.159 | 0 | 0 | 0 | flat_flat |
| hsa-miR-1911 | -0.670 | -0.610 | -0.843 | -1.134 | -0.291 | -0.721 | 0.515 | 1.000 | -0.203 | -0.581 | 0.595 | 1.000 | -0.494 | -1.413 | 0.237 | 1.000 | 0 | 0 | 0 | flat_flat |
| hsa-miR-1911* | -0.990 | -0.631 | -0.669 | -1.040 | -0.372 | -0.836 | 0.455 | 1.000 | 0.142 | 0.369 | 0.733 | 1.000 | -0.230 | -0.597 | 0.586 | 1.000 | 0 | 0 | 0 | flat_flat |
| hsa-miR-1912 | 0.067 | -0.521 | 0.050 | -1.146 | -1.196 | -2.359 | 0.084 | 0.544 | 0.277 | 0.631 | 0.565 | 1.000 | -0.919 | -2.093 | 0.112 | 0.657 | 0 | 0 | 0 | flat_flat |
| hsa-miR-1914 | 0.322 | -0.114 | -1.907 | 0.044 | 1.951 | 4.218 | 0.017 | 0.172 | -2.011 | -5.019 | 0.010 | 0.138 | -0.060 | -0.149 | 0.889 | 1.000 | 0 | 0 | 0 | flat_flat |
| hsa-miR-1914* | 11.212 | 12.807 | 8.112 | 13.006 | 4.893 | 5.266 | 0.008 | 0.106 | -3.897 | -4.842 | 0.011 | 0.147 | 0.996 | 1.238 | 0.290 | 1.000 | 0 | 0 | 0 | flat_flat |
| hsa-miR-1915 | 14.131 | 14.013 | 14.392 | 13.354 | -1.038 | -2.551 | 0.070 | 0.471 | 0.320 | 0.909 | 0.420 | 1.000 | -0.718 | -2.037 | 0.119 | 0.680 | 0 | 0 | 0 | flat_flat |
| hsa-miR-1915* | 0.553 | 0.908 | -1.565 | -1.766 | -0.201 | -0.453 | 0.676 | 1.000 | -2.295 | -5.979 | 0.005 | 0.100 | -2.496 | -6.503 | 0.004 | 0.060 | 0 | 0 | 0 | flat_flat |
| hsa-miR-192* | -0.648 | -0.073 | -0.704 | -0.163 | 0.541 | 1.076 | 0.348 | 1.000 | -0.344 | -0.789 | 0.479 | 1.000 | 0.198 | 0.454 | 0.676 | 1.000 | 0 | 0 | 0 | flat_flat |
| hsa-miR-193b* | -1.003 | -0.399 | -0.916 | -0.951 | -0.035 | -0.069 | 0.949 | 1.000 | -0.215 | -0.485 | 0.656 | 1.000 | -0.251 | -0.565 | 0.605 | 1.000 | 0 | 0 | 0 | flat_flat |
| hsa-miR-194* | -1.069 | -0.260 | -1.218 | -0.526 | 0.692 | 1.184 | 0.308 | 1.000 | -0.554 | -1.094 | 0.341 | 1.000 | 0.138 | 0.273 | 0.800 | 1.000 | 0 | 0 | 0 | flat_flat |
| hsa-miR-195* | 0.443 | 0.134 | -1.346 | 1.191 | 2.537 | 5.851 | 0.006 | 0.083 | -1.635 | -4.354 | 0.015 | 0.179 | 0.902 | 2.403 | 0.081 | 0.516 | 0 | 0 | 0 | flat_flat |
| hsa-miR-196a | 0.275 | 0.303 | 2.172 | 0.642 | -1.530 | -3.801 | 0.023 | 0.216 | 1.883 | 5.401 | 0.007 | 0.118 | 0.353 | 1.012 | 0.374 | 1.000 | 0 | 0 | 0 | flat_flat |
| hsa-miR-196a* | -2.784 | -2.276 | -1.500 | -1.481 | 0.019 | 0.040 | 0.970 | 1.000 | 1.030 | 2.466 | 0.076 | 0.501 | 1.050 | 2.512 | 0.072 | 0.480 | 0 | 0 | 0 | flat_flat |
| hsa-miR-197 | 12.579 | 12.579 | 12.579 | 12.284 | -0.295 | -0.734 | 0.508 | 1.000 | 0.000 | 0.000 | 1.000 | 1.000 | -0.295 | -0.847 | 0.449 | 1.000 | 0 | 0 | 0 | flat_flat |
| hsa-miR-199b-5p | 8.929 | 8.342 | 6.669 | 9.457 | 2.788 | 5.501 | 0.007 | 0.096 | -1.966 | -4.479 | 0.014 | 0.170 | 0.822 | 1.872 | 0.142 | 0.772 | 0 | 0 | 0 | flat_flat |
| hsa-miR-19a | 13.522 | 13.482 | 13.681 | 13.432 | -0.248 | -0.617 | 0.574 | 1.000 | 0.179 | 0.512 | 0.638 | 1.000 | -0.070 | -0.200 | 0.852 | 1.000 | 0 | 0 | 0 | flat_flat |
| hsa-miR-19a* | -1.197 | -1.134 | -2.599 | -0.345 | 2.254 | 5.584 | 0.007 | 0.092 | -1.433 | -4.100 | 0.018 | 0.200 | 0.821 | 2.348 | 0.085 | 0.537 | 0 | 0 | 0 | flat_flat |
| hsa-miR-19b | 12.269 | 12.216 | 12.490 | 11.212 | -1.278 | -3.170 | 0.039 | 0.315 | 0.247 | 0.708 | 0.522 | 1.000 | -1.031 | -2.952 | 0.047 | 0.352 | 0 | 0 | 0 | flat_flat |
| hsa-miR-19b-1* | 4.860 | 3.733 | 6.119 | 4.919 | -1.200 | -1.677 | 0.176 | 0.943 | 1.822 | 2.940 | 0.048 | 0.360 | 0.622 | 1.004 | 0.378 | 1.000 | 0 | 0 | 0 | flat_flat |
| hsa-miR-19b-2* | -0.151 | -0.250 | 0.845 | 0.762 | -0.083 | -0.204 | 0.850 | 1.000 | 1.045 | 2.975 | 0.046 | 0.354 | 0.962 | 2.740 | 0.058 | 0.402 | 0 | 0 | 0 | flat_flat |
| hsa-miR-200a | 1.582 | 1.313 | 0.762 | 0.845 | 0.083 | 0.194 | 0.857 | 1.000 | -0.686 | -1.857 | 0.144 | 0.778 | -0.603 | -1.634 | 0.185 | 0.954 | 0 | 0 | 0 | flat_flat |
| hsa-miR-200a* | -0.916 | -0.699 | -0.412 | -1.654 | -1.242 | -2.972 | 0.046 | 0.360 | 0.395 | 1.092 | 0.342 | 1.000 | -0.847 | -2.340 | 0.086 | 0.539 | 0 | 0 | 0 | flat_flat |
| hsa-miR-200b* | -1.897 | -1.814 | -1.963 | -1.714 | 0.249 | 0.614 | 0.575 | 1.000 | -0.107 | -0.306 | 0.776 | 1.000 | 0.141 | 0.403 | 0.709 | 1.000 | 0 | 0 | 0 | flat_flat |
| hsa-miR-200c* | -0.371 | -0.570 | -0.556 | 0.103 | 0.658 | 1.584 | 0.196 | 1.000 | -0.085 | -0.237 | 0.825 | 1.000 | 0.573 | 1.592 | 0.194 | 0.982 | 0 | 0 | 0 | flat_flat |
| hsa-miR-202* | -0.356 | -0.127 | -0.710 | 0.163 | 0.873 | 2.080 | 0.113 | 0.677 | -0.469 | -1.289 | 0.273 | 1.000 | 0.404 | 1.113 | 0.334 | 1.000 | 0 | 0 | 0 | flat_flat |
| hsa-miR-203 | -1.183 | -2.150 | -0.221 | -1.005 | -0.785 | -1.212 | 0.299 | 1.000 | 1.446 | 2.578 | 0.068 | 0.462 | 0.661 | 1.179 | 0.310 | 1.000 | 0 | 0 | 0 | flat_flat |
| hsa-miR-206 | 1.086 | 1.635 | 0.886 | -0.238 | -1.125 | -2.273 | 0.092 | 0.579 | -0.474 | -1.107 | 0.336 | 1.000 | -1.599 | -3.731 | 0.024 | 0.222 | 0 | 0 | 0 | flat_flat |
| hsa-miR-208a | -1.612 | -1.599 | -1.073 | -1.550 | -0.477 | -1.186 | 0.307 | 1.000 | 0.532 | 1.528 | 0.208 | 1.000 | 0.055 | 0.158 | 0.883 | 1.000 | 0 | 0 | 0 | flat_flat |
| hsa-miR-208b | -1.447 | -1.975 | -0.997 | -1.360 | -0.363 | -0.744 | 0.502 | 1.000 | 0.714 | 1.688 | 0.174 | 0.889 | 0.351 | 0.829 | 0.458 | 1.000 | 0 | 0 | 0 | flat_flat |
| hsa-miR-20a | 13.306 | 13.316 | 13.749 | 13.653 | -0.096 | -0.239 | 0.824 | 1.000 | 0.438 | 1.257 | 0.283 | 1.000 | 0.342 | 0.982 | 0.387 | 1.000 | 0 | 0 | 0 | flat_flat |
| hsa-miR-20a* | 4.513 | 3.649 | 5.894 | 6.669 | 0.775 | 1.278 | 0.277 | 1.000 | 1.813 | 3.452 | 0.030 | 0.276 | 2.588 | 4.928 | 0.010 | 0.118 | 0 | 0 | 0 | flat_flat |
| hsa-miR-20b | 13.749 | 13.681 | 13.794 | 12.739 | -1.055 | -2.613 | 0.065 | 0.450 | 0.079 | 0.226 | 0.833 | 1.000 | -0.976 | -2.791 | 0.055 | 0.390 | 0 | 0 | 0 | flat_flat |
| hsa-miR-20b* | -1.609 | -1.654 | -1.599 | -1.733 | -0.134 | -0.332 | 0.758 | 1.000 | 0.032 | 0.093 | 0.931 | 1.000 | -0.101 | -0.290 | 0.788 | 1.000 | 0 | 0 | 0 | flat_flat |
| hsa-miR-21 | 12.727 | 12.612 | 13.025 | 11.951 | -1.074 | -2.640 | 0.064 | 0.441 | 0.355 | 1.009 | 0.376 | 1.000 | -0.718 | -2.039 | 0.118 | 0.680 | 0 | 0 | 0 | flat_flat |
| hsa-miR-211 | 2.196 | 1.857 | 0.721 | 2.196 | 1.475 | 3.354 | 0.033 | 0.281 | -1.306 | -3.428 | 0.031 | 0.279 | 0.170 | 0.445 | 0.682 | 1.000 | 0 | 0 | 0 | flat_flat |
| hsa-miR-214* | 0.350 | -0.036 | 0.536 | -1.197 | -1.734 | -3.850 | 0.022 | 0.210 | 0.379 | 0.973 | 0.391 | 1.000 | -1.354 | -3.473 | 0.030 | 0.256 | 0 | 0 | 0 | flat_flat |
| hsa-miR-216a | -1.948 | -0.958 | -1.775 | -1.877 | -0.102 | -0.155 | 0.885 | 1.000 | -0.322 | -0.566 | 0.605 | 1.000 | -0.424 | -0.745 | 0.502 | 1.000 | 0 | 0 | 0 | flat_flat |
| hsa-miR-216b | -2.659 | -3.098 | -1.392 | -2.276 | -0.885 | -1.909 | 0.136 | 0.783 | 1.487 | 3.704 | 0.025 | 0.241 | 0.602 | 1.500 | 0.215 | 1.000 | 0 | 0 | 0 | flat_flat |
| hsa-miR-217 | -2.603 | -2.574 | -1.714 | -2.574 | -0.860 | -2.137 | 0.106 | 0.647 | 0.875 | 2.509 | 0.073 | 0.485 | 0.014 | 0.041 | 0.969 | 1.000 | 0 | 0 | 0 | flat_flat |
| hsa-miR-218-1* | -2.868 | -2.064 | -2.820 | -2.698 | 0.122 | 0.209 | 0.846 | 1.000 | -0.354 | -0.701 | 0.526 | 1.000 | -0.232 | -0.460 | 0.672 | 1.000 | 0 | 0 | 0 | flat_flat |
| hsa-miR-218-2* | -1.676 | -1.481 | -1.411 | -2.483 | -1.073 | -2.584 | 0.067 | 0.462 | 0.168 | 0.467 | 0.667 | 1.000 | -0.905 | -2.517 | 0.072 | 0.480 | 0 | 0 | 0 | flat_flat |
| hsa-miR-219-1-3p | -1.623 | -2.017 | -2.784 | -0.798 | 1.986 | 4.390 | 0.015 | 0.161 | -0.964 | -2.460 | 0.076 | 0.503 | 1.022 | 2.609 | 0.066 | 0.445 | 0 | 0 | 0 | flat_flat |
| hsa-miR-219-2-3p | 0.577 | 0.598 | -0.382 | -1.086 | -0.704 | -1.749 | 0.163 | 0.891 | -0.969 | -2.781 | 0.056 | 0.401 | -1.673 | -4.800 | 0.011 | 0.127 | 0 | 0 | 0 | flat_flat |
| hsa-miR-22 | 11.891 | 11.982 | 12.388 | 10.560 | -1.828 | -4.514 | 0.013 | 0.151 | 0.452 | 1.288 | 0.274 | 1.000 | -1.377 | -3.924 | 0.021 | 0.195 | 0 | 0 | 0 | flat_flat |
| hsa-miR-22* | -0.153 | -0.790 | 0.904 | -0.843 | -1.747 | -3.340 | 0.033 | 0.284 | 1.376 | 3.037 | 0.044 | 0.341 | -0.371 | -0.819 | 0.463 | 1.000 | 0 | 0 | 0 | flat_flat |
| hsa-miR-220a | -0.946 | -0.556 | -0.255 | -0.436 | -0.181 | -0.400 | 0.711 | 1.000 | 0.496 | 1.267 | 0.280 | 1.000 | 0.315 | 0.805 | 0.470 | 1.000 | 0 | 0 | 0 | flat_flat |
| hsa-miR-220b | -0.750 | -1.307 | -3.628 | -1.185 | 2.443 | 4.911 | 0.010 | 0.128 | -2.599 | -6.035 | 0.005 | 0.100 | -0.157 | -0.363 | 0.736 | 1.000 | 0 | 0 | 0 | flat_flat |
| hsa-miR-220c | -0.843 | -1.747 | -0.329 | -0.916 | -0.587 | -0.943 | 0.404 | 1.000 | 0.966 | 1.793 | 0.155 | 0.816 | 0.379 | 0.704 | 0.524 | 1.000 | 0 | 0 | 0 | flat_flat |
| hsa-miR-221 | 10.267 | 10.426 | 9.085 | 11.891 | 2.806 | 6.830 | 0.003 | 0.058 | -1.261 | -3.544 | 0.028 | 0.262 | 1.545 | 4.343 | 0.015 | 0.159 | 0 | 0 | 0 | flat_flat |
| hsa-miR-222* | -0.297 | 0.196 | -1.790 | -1.487 | 0.302 | 0.632 | 0.565 | 1.000 | -1.739 | -4.198 | 0.017 | 0.191 | -1.437 | -3.467 | 0.030 | 0.256 | 0 | 0 | 0 | flat_flat |
| hsa-miR-23a | 11.824 | 11.370 | 12.702 | 12.507 | -0.195 | -0.416 | 0.701 | 1.000 | 1.105 | 2.729 | 0.058 | 0.415 | 0.910 | 2.248 | 0.095 | 0.584 | 0 | 0 | 0 | flat_flat |
| hsa-miR-23b* | -0.188 | -0.543 | 0.791 | 0.504 | -0.287 | -0.648 | 0.555 | 1.000 | 1.157 | 3.015 | 0.045 | 0.344 | 0.870 | 2.266 | 0.093 | 0.576 | 0 | 0 | 0 | flat_flat |
| hsa-miR-24 | 13.869 | 13.749 | 14.013 | 14.013 | 0.000 | 0.000 | 1.000 | 1.000 | 0.203 | 0.577 | 0.598 | 1.000 | 0.203 | 0.577 | 0.598 | 1.000 | 0 | 0 | 0 | flat_flat |
| hsa-miR-24-1* | 0.105 | -0.131 | -1.210 | -0.494 | 0.716 | 1.701 | 0.171 | 0.923 | -1.198 | -3.286 | 0.035 | 0.295 | -0.482 | -1.321 | 0.264 | 1.000 | 0 | 0 | 0 | flat_flat |
| hsa-miR-24-2* | -2.264 | -2.586 | -2.505 | -2.548 | -0.043 | -0.099 | 0.927 | 1.000 | -0.080 | -0.211 | 0.844 | 1.000 | -0.123 | -0.325 | 0.763 | 1.000 | 0 | 0 | 0 | flat_flat |
| hsa-miR-25 | 11.069 | 11.396 | 12.142 | 11.370 | -0.772 | -1.766 | 0.160 | 0.879 | 0.909 | 2.402 | 0.081 | 0.517 | 0.137 | 0.363 | 0.737 | 1.000 | 0 | 0 | 0 | flat_flat |
| hsa-miR-25* | 0.148 | -0.812 | 0.025 | -0.777 | -0.801 | -1.242 | 0.288 | 1.000 | 0.357 | 0.639 | 0.561 | 1.000 | -0.444 | -0.796 | 0.475 | 1.000 | 0 | 0 | 0 | flat_flat |
| hsa-miR-26a | 11.272 | 11.272 | 12.395 | 12.872 | 0.477 | 1.186 | 0.307 | 1.000 | 1.123 | 3.223 | 0.037 | 0.300 | 1.600 | 4.593 | 0.013 | 0.141 | 0 | 0 | 0 | flat_flat |
| hsa-miR-26a-1* | -0.197 | -0.285 | 0.423 | 1.526 | 1.103 | 2.723 | 0.059 | 0.422 | 0.664 | 1.893 | 0.139 | 0.754 | 1.766 | 5.038 | 0.009 | 0.111 | 0 | 0 | 0 | flat_flat |
| hsa-miR-26a-2* | 0.228 | 0.557 | 0.291 | 1.709 | 1.418 | 3.240 | 0.036 | 0.303 | -0.102 | -0.268 | 0.803 | 1.000 | 1.316 | 3.473 | 0.030 | 0.256 | 0 | 0 | 0 | flat_flat |
| hsa-miR-26b | 11.580 | 10.340 | 13.272 | 13.618 | 0.346 | 0.452 | 0.677 | 1.000 | 2.312 | 3.486 | 0.030 | 0.271 | 2.658 | 4.009 | 0.019 | 0.187 | 0 | 0 | 0 | flat_flat |
| hsa-miR-26b* | 0.251 | 0.105 | -0.096 | 1.466 | 1.561 | 3.813 | 0.023 | 0.215 | -0.274 | -0.771 | 0.488 | 1.000 | 1.288 | 3.632 | 0.026 | 0.234 | 0 | 0 | 0 | flat_flat |
| hsa-miR-27a | 12.523 | 12.439 | 13.143 | 12.439 | -0.704 | -1.741 | 0.164 | 0.897 | 0.662 | 1.889 | 0.139 | 0.756 | -0.042 | -0.121 | 0.910 | 1.000 | 0 | 0 | 0 | flat_flat |
| hsa-miR-27a* | -1.879 | -1.676 | -2.669 | -1.529 | 1.140 | 2.739 | 0.058 | 0.420 | -0.891 | -2.474 | 0.075 | 0.500 | 0.248 | 0.690 | 0.532 | 1.000 | 0 | 0 | 0 | flat_flat |
| hsa-miR-27b | 12.407 | 12.243 | 12.284 | 11.685 | -0.599 | -1.455 | 0.226 | 1.000 | -0.042 | -0.117 | 0.913 | 1.000 | -0.640 | -1.797 | 0.154 | 0.819 | 0 | 0 | 0 | flat_flat |
| hsa-miR-27b* | -1.939 | -0.120 | -1.183 | -1.790 | -0.606 | -0.585 | 0.593 | 1.000 | -0.154 | -0.171 | 0.873 | 1.000 | -0.760 | -0.847 | 0.449 | 1.000 | 0 | 0 | 0 | flat_flat |
| hsa-miR-28-3p | -1.360 | -0.725 | -0.924 | -1.434 | -0.510 | -0.977 | 0.389 | 1.000 | 0.119 | 0.263 | 0.806 | 1.000 | -0.391 | -0.865 | 0.441 | 1.000 | 0 | 0 | 0 | flat_flat |
| hsa-miR-28-5p | 7.856 | 6.794 | 11.475 | 8.437 | -3.038 | -4.418 | 0.014 | 0.160 | 4.150 | 6.969 | 0.003 | 0.073 | 1.112 | 1.868 | 0.143 | 0.773 | 0 | 0 | 0 | flat_flat |
| hsa-miR-296-3p | -0.460 | -1.877 | -1.700 | -2.659 | -0.959 | -1.134 | 0.326 | 1.000 | -0.532 | -0.726 | 0.512 | 1.000 | -1.491 | -2.035 | 0.119 | 0.680 | 0 | 0 | 0 | flat_flat |
| hsa-miR-296-5p | 4.358 | 4.609 | 7.182 | 5.778 | -1.404 | -3.317 | 0.034 | 0.287 | 2.698 | 7.360 | 0.003 | 0.066 | 1.294 | 3.530 | 0.028 | 0.247 | 0 | 0 | 0 | flat_flat |
| hsa-miR-297 | -1.832 | -0.363 | -0.087 | -1.073 | -0.986 | -1.134 | 0.326 | 1.000 | 1.011 | 1.342 | 0.258 | 1.000 | 0.024 | 0.032 | 0.976 | 1.000 | 0 | 0 | 0 | flat_flat |
| hsa-miR-298 | -1.643 | -1.108 | -1.185 | -1.234 | -0.049 | -0.100 | 0.926 | 1.000 | 0.190 | 0.447 | 0.680 | 1.000 | 0.141 | 0.332 | 0.758 | 1.000 | 0 | 0 | 0 | flat_flat |
| hsa-miR-299-3p | -1.500 | -1.069 | -0.451 | -0.750 | -0.299 | -0.649 | 0.555 | 1.000 | 0.833 | 2.084 | 0.113 | 0.657 | 0.534 | 1.335 | 0.260 | 1.000 | 0 | 0 | 0 | flat_flat |
| hsa-miR-299-5p | 0.729 | 0.892 | 0.902 | 1.990 | 1.088 | 2.646 | 0.063 | 0.440 | 0.092 | 0.258 | 0.810 | 1.000 | 1.180 | 3.313 | 0.034 | 0.281 | 0 | 0 | 0 | flat_flat |
| hsa-miR-29a* | 0.269 | -0.159 | -0.114 | 0.904 | 1.018 | 2.210 | 0.099 | 0.608 | -0.169 | -0.423 | 0.696 | 1.000 | 0.849 | 2.128 | 0.107 | 0.646 | 0 | 0 | 0 | flat_flat |
| hsa-miR-29b-1* | 0.858 | 0.645 | 1.626 | 2.884 | 1.257 | 3.012 | 0.045 | 0.352 | 0.875 | 2.421 | 0.079 | 0.515 | 2.132 | 5.899 | 0.006 | 0.076 | 0 | 0 | 0 | flat_flat |
| hsa-miR-29b-2* | -0.812 | -1.357 | -0.863 | -1.559 | -0.696 | -1.409 | 0.239 | 1.000 | 0.222 | 0.519 | 0.634 | 1.000 | -0.474 | -1.108 | 0.336 | 1.000 | 0 | 0 | 0 | flat_flat |
| hsa-miR-29c* | 0.898 | 0.163 | 0.325 | 3.649 | 3.324 | 5.961 | 0.005 | 0.080 | -0.206 | -0.426 | 0.694 | 1.000 | 3.118 | 6.456 | 0.004 | 0.061 | 0 | 0 | 0 | flat_flat |
| hsa-miR-300 | 1.990 | 2.965 | 0.375 | -0.652 | -1.026 | -1.576 | 0.197 | 1.000 | -2.103 | -3.729 | 0.024 | 0.240 | -3.129 | -5.549 | 0.007 | 0.088 | 0 | 0 | 0 | flat_flat |
| hsa-miR-302d* | -1.403 | -2.056 | -2.603 | -2.126 | 0.477 | 0.903 | 0.422 | 1.000 | -0.873 | -1.908 | 0.136 | 0.747 | -0.396 | -0.865 | 0.440 | 1.000 | 0 | 0 | 0 | flat_flat |
| hsa-miR-302e | -1.121 | -1.016 | -1.832 | -0.521 | 1.311 | 3.228 | 0.037 | 0.304 | -0.763 | -2.171 | 0.103 | 0.614 | 0.547 | 1.556 | 0.202 | 1.000 | 0 | 0 | 0 | flat_flat |
| hsa-miR-302f | -1.364 | -1.851 | -1.572 | 0.153 | 1.725 | 3.618 | 0.026 | 0.240 | 0.035 | 0.086 | 0.936 | 1.000 | 1.760 | 4.264 | 0.016 | 0.162 | 0 | 0 | 0 | flat_flat |
| hsa-miR-30a | 8.135 | 7.003 | 10.426 | 7.182 | -3.244 | -4.520 | 0.013 | 0.151 | 2.857 | 4.597 | 0.013 | 0.159 | -0.387 | -0.622 | 0.571 | 1.000 | 0 | 0 | 0 | flat_flat |
| hsa-miR-30b | 10.166 | 7.881 | 12.216 | 13.946 | 1.730 | 1.367 | 0.250 | 1.000 | 3.193 | 2.914 | 0.049 | 0.367 | 4.923 | 4.492 | 0.014 | 0.146 | 0 | 0 | 0 | flat_flat |
| hsa-miR-30b* | 0.413 | 1.173 | 0.627 | 2.754 | 2.127 | 3.753 | 0.024 | 0.220 | -0.166 | -0.338 | 0.754 | 1.000 | 1.961 | 3.996 | 0.020 | 0.188 | 0 | 0 | 0 | flat_flat |
| hsa-miR-30c-1* | 0.182 | -0.253 | 0.461 | 0.303 | -0.158 | -0.341 | 0.752 | 1.000 | 0.497 | 1.240 | 0.289 | 1.000 | 0.339 | 0.846 | 0.450 | 1.000 | 0 | 0 | 0 | flat_flat |
| hsa-miR-30c-2* | -0.416 | -0.087 | 1.526 | -1.031 | -2.557 | -5.839 | 0.006 | 0.083 | 1.777 | 4.687 | 0.012 | 0.156 | -0.779 | -2.055 | 0.116 | 0.675 | 0 | 0 | 0 | flat_flat |
| hsa-miR-30d* | -0.644 | -1.635 | 0.313 | 0.196 | -0.117 | -0.179 | 0.868 | 1.000 | 1.453 | 2.549 | 0.070 | 0.473 | 1.335 | 2.343 | 0.086 | 0.538 | 0 | 0 | 0 | flat_flat |
| hsa-miR-30e | 9.199 | 8.060 | 9.199 | 11.719 | 2.520 | 3.497 | 0.029 | 0.261 | 0.569 | 0.912 | 0.418 | 1.000 | 3.089 | 4.950 | 0.010 | 0.117 | 0 | 0 | 0 | flat_flat |
| hsa-miR-30e* | 3.674 | 2.315 | 6.911 | 7.270 | 0.359 | 0.438 | 0.686 | 1.000 | 3.917 | 5.520 | 0.007 | 0.113 | 4.276 | 6.026 | 0.005 | 0.073 | 0 | 0 | 0 | flat_flat |
| hsa-miR-32 | 3.733 | 2.583 | -0.561 | 1.185 | 1.746 | 2.406 | 0.081 | 0.527 | -3.718 | -5.916 | 0.006 | 0.102 | -1.972 | -3.138 | 0.040 | 0.315 | 0 | 0 | 0 | flat_flat |
| hsa-miR-32* | 11.170 | 13.544 | 3.113 | 8.620 | 5.506 | 4.204 | 0.017 | 0.172 | -9.244 | -8.150 | 0.002 | 0.053 | -3.738 | -3.295 | 0.035 | 0.282 | 0 | 0 | 0 | flat_flat |
| hsa-miR-320a | 11.773 | 11.951 | 11.842 | 11.824 | -0.018 | -0.043 | 0.968 | 1.000 | -0.020 | -0.057 | 0.958 | 1.000 | -0.038 | -0.106 | 0.921 | 1.000 | 0 | 0 | 0 | flat_flat |
| hsa-miR-320b | 12.612 | 12.739 | 12.523 | 11.862 | -0.661 | -1.621 | 0.188 | 0.989 | -0.152 | -0.431 | 0.691 | 1.000 | -0.813 | -2.303 | 0.090 | 0.559 | 0 | 0 | 0 | flat_flat |
| hsa-miR-320c | 10.046 | 10.676 | 11.544 | 10.426 | -1.119 | -2.147 | 0.105 | 0.642 | 1.183 | 2.622 | 0.065 | 0.447 | 0.065 | 0.143 | 0.894 | 1.000 | 0 | 0 | 0 | flat_flat |
| hsa-miR-320d | 12.395 | 12.388 | 12.739 | 11.657 | -1.082 | -2.690 | 0.061 | 0.429 | 0.347 | 0.997 | 0.380 | 1.000 | -0.735 | -2.109 | 0.110 | 0.653 | 0 | 0 | 0 | flat_flat |
| hsa-miR-323-3p | -0.630 | -0.371 | -0.320 | -0.863 | -0.543 | -1.280 | 0.276 | 1.000 | 0.181 | 0.491 | 0.652 | 1.000 | -0.363 | -0.987 | 0.385 | 1.000 | 0 | 0 | 0 | flat_flat |
| hsa-miR-323-5p | -2.398 | -1.775 | -3.014 | -2.014 | 1.000 | 1.929 | 0.133 | 0.770 | -0.927 | -2.065 | 0.115 | 0.666 | 0.073 | 0.162 | 0.880 | 1.000 | 0 | 0 | 0 | flat_flat |
| hsa-miR-325 | -1.907 | -2.868 | -0.696 | -1.500 | -0.804 | -1.246 | 0.287 | 1.000 | 1.692 | 3.027 | 0.044 | 0.342 | 0.888 | 1.588 | 0.195 | 0.984 | 0 | 0 | 0 | flat_flat |
| hsa-miR-326 | 1.110 | 0.585 | 3.205 | 2.285 | -0.920 | -1.888 | 0.139 | 0.796 | 2.357 | 5.582 | 0.007 | 0.112 | 1.437 | 3.402 | 0.032 | 0.266 | 0 | 0 | 0 | flat_flat |
| hsa-miR-328 | 5.778 | 6.119 | 3.733 | 4.695 | 0.962 | 2.186 | 0.101 | 0.622 | -2.215 | -5.811 | 0.006 | 0.106 | -1.253 | -3.287 | 0.035 | 0.282 | 0 | 0 | 0 | flat_flat |
| hsa-miR-329 | -0.131 | -0.012 | -0.275 | 1.582 | 1.857 | 4.562 | 0.013 | 0.149 | -0.203 | -0.577 | 0.598 | 1.000 | 1.654 | 4.691 | 0.012 | 0.134 | 0 | 0 | 0 | flat_flat |
| hsa-miR-330-3p | -0.163 | 2.111 | 2.303 | 1.926 | -0.377 | -0.299 | 0.781 | 1.000 | 1.329 | 1.218 | 0.296 | 1.000 | 0.952 | 0.873 | 0.437 | 1.000 | 0 | 0 | 0 | flat_flat |
| hsa-miR-330-5p | -0.829 | -0.577 | -0.166 | -1.105 | -0.939 | -2.217 | 0.098 | 0.605 | 0.537 | 1.463 | 0.224 | 1.000 | -0.402 | -1.097 | 0.340 | 1.000 | 0 | 0 | 0 | flat_flat |
| hsa-miR-331-3p | 12.807 | 12.702 | 13.316 | 12.825 | -0.491 | -1.209 | 0.300 | 1.000 | 0.561 | 1.597 | 0.193 | 0.959 | 0.071 | 0.201 | 0.851 | 1.000 | 0 | 0 | 0 | flat_flat |
| hsa-miR-331-5p | -1.529 | 0.004 | -1.307 | -1.202 | 0.105 | 0.117 | 0.913 | 1.000 | -0.545 | -0.699 | 0.527 | 1.000 | -0.439 | -0.564 | 0.606 | 1.000 | 0 | 0 | 0 | flat_flat |
| hsa-miR-335 | 5.613 | 5.613 | 3.229 | 4.246 | 1.016 | 2.527 | 0.071 | 0.477 | -2.383 | -6.841 | 0.003 | 0.077 | -1.367 | -3.924 | 0.021 | 0.195 | 0 | 0 | 0 | flat_flat |
| hsa-miR-337-3p | 0.622 | 0.999 | -0.053 | 1.496 | 1.549 | 3.455 | 0.030 | 0.262 | -0.864 | -2.225 | 0.097 | 0.589 | 0.685 | 1.765 | 0.160 | 0.845 | 0 | 0 | 0 | flat_flat |
| hsa-miR-337-5p | -0.807 | -1.399 | -0.780 | -0.221 | 0.560 | 1.101 | 0.339 | 1.000 | 0.323 | 0.734 | 0.508 | 1.000 | 0.883 | 2.005 | 0.123 | 0.693 | 0 | 0 | 0 | flat_flat |
| hsa-miR-339-3p | 0.325 | 0.904 | 1.575 | -0.845 | -2.421 | -4.799 | 0.011 | 0.134 | 0.961 | 2.199 | 0.100 | 0.600 | -1.460 | -3.342 | 0.033 | 0.278 | 0 | 0 | 0 | flat_flat |
| hsa-miR-339-5p | -1.733 | -2.082 | -1.862 | -2.113 | -0.251 | -0.568 | 0.603 | 1.000 | 0.046 | 0.119 | 0.912 | 1.000 | -0.205 | -0.537 | 0.623 | 1.000 | 0 | 0 | 0 | flat_flat |
| hsa-miR-33a | 1.857 | 1.248 | 0.960 | 0.627 | -0.333 | -0.648 | 0.556 | 1.000 | -0.592 | -1.331 | 0.261 | 1.000 | -0.925 | -2.079 | 0.113 | 0.662 | 0 | 0 | 0 | flat_flat |
| hsa-miR-33a* | -0.913 | -0.225 | -2.081 | -0.428 | 1.653 | 3.056 | 0.043 | 0.344 | -1.512 | -3.229 | 0.037 | 0.300 | 0.141 | 0.301 | 0.780 | 1.000 | 0 | 0 | 0 | flat_flat |
| hsa-miR-33b | -1.117 | -1.591 | -0.151 | -1.591 | -1.441 | -3.045 | 0.043 | 0.345 | 1.203 | 2.937 | 0.048 | 0.361 | -0.237 | -0.579 | 0.597 | 1.000 | 0 | 0 | 0 | flat_flat |
| hsa-miR-340* | 3.649 | 2.243 | 5.837 | 5.142 | -0.695 | -0.827 | 0.459 | 1.000 | 2.891 | 3.970 | 0.020 | 0.214 | 2.196 | 3.016 | 0.045 | 0.338 | 0 | 0 | 0 | flat_flat |
| hsa-miR-342-3p | 9.998 | 9.892 | 12.111 | 12.341 | 0.231 | 0.569 | 0.603 | 1.000 | 2.166 | 6.159 | 0.005 | 0.098 | 2.396 | 6.815 | 0.003 | 0.053 | 0 | 0 | 0 | flat_flat |
| hsa-miR-345 | 1.478 | 1.341 | 2.243 | -0.079 | -2.321 | -5.681 | 0.006 | 0.090 | 0.834 | 2.355 | 0.085 | 0.534 | -1.488 | -4.204 | 0.017 | 0.168 | 0 | 0 | 0 | flat_flat |
| hsa-miR-346 | 1.237 | 1.445 | -0.855 | 0.359 | 1.214 | 2.913 | 0.049 | 0.372 | -2.196 | -6.085 | 0.005 | 0.099 | -0.982 | -2.721 | 0.059 | 0.406 | 0 | 0 | 0 | flat_flat |
| hsa-miR-34a* | 0.461 | 0.234 | -0.100 | -0.394 | -0.293 | -0.699 | 0.527 | 1.000 | -0.448 | -1.234 | 0.291 | 1.000 | -0.741 | -2.041 | 0.118 | 0.680 | 0 | 0 | 0 | flat_flat |
| hsa-miR-34b | 1.672 | 1.646 | 1.519 | 0.985 | -0.534 | -1.327 | 0.262 | 1.000 | -0.141 | -0.404 | 0.709 | 1.000 | -0.675 | -1.935 | 0.132 | 0.734 | 0 | 0 | 0 | flat_flat |
| hsa-miR-34b* | 4.919 | 3.771 | 4.919 | 0.228 | -4.690 | -6.475 | 0.004 | 0.065 | 0.574 | 0.914 | 0.417 | 1.000 | -4.117 | -6.562 | 0.004 | 0.059 | 0 | 0 | 0 | flat_flat |
| hsa-miR-34c-3p | -1.114 | -0.780 | -0.990 | -2.747 | -1.757 | -4.003 | 0.019 | 0.194 | -0.043 | -0.113 | 0.916 | 1.000 | -1.800 | -4.736 | 0.011 | 0.131 | 0 | 0 | 0 | flat_flat |
| hsa-miR-34c-5p | 1.161 | 0.729 | 1.445 | 0.097 | -1.348 | -2.919 | 0.049 | 0.372 | 0.500 | 1.251 | 0.286 | 1.000 | -0.848 | -2.120 | 0.108 | 0.648 | 0 | 0 | 0 | flat_flat |
| hsa-miR-361-5p | 11.370 | 11.299 | 12.243 | 11.773 | -0.471 | -1.165 | 0.315 | 1.000 | 0.909 | 2.599 | 0.066 | 0.454 | 0.439 | 1.254 | 0.285 | 1.000 | 0 | 0 | 0 | flat_flat |
| hsa-miR-362-3p | 1.830 | 1.526 | 3.576 | 1.285 | -2.291 | -5.294 | 0.008 | 0.105 | 1.898 | 5.064 | 0.009 | 0.137 | -0.393 | -1.049 | 0.359 | 1.000 | 0 | 0 | 0 | flat_flat |
| hsa-miR-363 | 13.894 | 13.840 | 13.894 | 13.869 | -0.024 | -0.061 | 0.955 | 1.000 | 0.027 | 0.077 | 0.943 | 1.000 | 0.002 | 0.007 | 0.995 | 1.000 | 0 | 0 | 0 | flat_flat |
| hsa-miR-363* | -0.218 | -0.729 | 0.354 | -1.747 | -2.102 | -4.347 | 0.015 | 0.163 | 0.828 | 1.978 | 0.126 | 0.714 | -1.273 | -3.041 | 0.044 | 0.334 | 0 | 0 | 0 | flat_flat |
| hsa-miR-367* | -1.185 | -1.612 | -1.007 | -0.953 | 0.054 | 0.117 | 0.913 | 1.000 | 0.391 | 0.982 | 0.387 | 1.000 | 0.445 | 1.116 | 0.333 | 1.000 | 0 | 0 | 0 | flat_flat |
| hsa-miR-369-3p | -1.040 | -0.843 | -0.297 | -0.561 | -0.263 | -0.634 | 0.564 | 1.000 | 0.644 | 1.791 | 0.155 | 0.816 | 0.381 | 1.059 | 0.355 | 1.000 | 0 | 0 | 0 | flat_flat |
| hsa-miR-369-5p | 0.336 | -0.188 | -0.265 | 0.115 | 0.380 | 0.779 | 0.484 | 1.000 | -0.339 | -0.802 | 0.472 | 1.000 | 0.041 | 0.098 | 0.927 | 1.000 | 0 | 0 | 0 | flat_flat |
| hsa-miR-370 | -0.096 | -0.153 | 0.099 | -0.041 | -0.140 | -0.346 | 0.748 | 1.000 | 0.223 | 0.640 | 0.561 | 1.000 | 0.084 | 0.240 | 0.824 | 1.000 | 0 | 0 | 0 | flat_flat |
| hsa-miR-371-5p | 12.030 | 10.889 | 10.942 | 8.748 | -2.194 | -3.040 | 0.044 | 0.345 | -0.517 | -0.828 | 0.459 | 1.000 | -2.712 | -4.338 | 0.015 | 0.159 | 0 | 0 | 0 | flat_flat |
| hsa-miR-373* | 1.035 | 0.886 | 0.775 | -0.421 | -1.196 | -2.920 | 0.049 | 0.372 | -0.185 | -0.523 | 0.632 | 1.000 | -1.382 | -3.894 | 0.021 | 0.198 | 0 | 0 | 0 | flat_flat |
| hsa-miR-374a | 13.840 | 13.522 | 12.439 | 11.272 | -1.167 | -2.679 | 0.061 | 0.430 | -1.242 | -3.293 | 0.035 | 0.295 | -2.409 | -6.387 | 0.004 | 0.061 | 0 | 0 | 0 | flat_flat |
| hsa-miR-374a* | -2.136 | -2.204 | -0.073 | -0.225 | -0.151 | -0.375 | 0.729 | 1.000 | 2.097 | 5.996 | 0.005 | 0.100 | 1.945 | 5.563 | 0.007 | 0.088 | 0 | 0 | 0 | flat_flat |
| hsa-miR-374b | 13.653 | 13.006 | 12.902 | 12.579 | -0.323 | -0.614 | 0.576 | 1.000 | -0.428 | -0.937 | 0.407 | 1.000 | -0.751 | -1.646 | 0.182 | 0.951 | 0 | 0 | 0 | flat_flat |
| hsa-miR-374b* | 0.188 | -0.392 | 0.504 | -0.246 | -0.750 | -1.486 | 0.219 | 1.000 | 0.606 | 1.386 | 0.245 | 1.000 | -0.144 | -0.330 | 0.760 | 1.000 | 0 | 0 | 0 | flat_flat |
| hsa-miR-376a | -0.159 | -0.273 | -1.254 | 0.557 | 1.810 | 4.451 | 0.014 | 0.157 | -1.038 | -2.947 | 0.048 | 0.359 | 0.773 | 2.193 | 0.100 | 0.610 | 0 | 0 | 0 | flat_flat |
| hsa-miR-376a* | -0.345 | 0.099 | -0.510 | 0.972 | 1.482 | 3.188 | 0.038 | 0.312 | -0.387 | -0.962 | 0.396 | 1.000 | 1.095 | 2.719 | 0.059 | 0.406 | 0 | 0 | 0 | flat_flat |
| hsa-miR-376b | -1.368 | -1.242 | -1.609 | -1.447 | 0.162 | 0.399 | 0.713 | 1.000 | -0.305 | -0.863 | 0.442 | 1.000 | -0.142 | -0.402 | 0.710 | 1.000 | 0 | 0 | 0 | flat_flat |
| hsa-miR-377 | -0.267 | -0.588 | -0.958 | 0.622 | 1.580 | 3.624 | 0.026 | 0.240 | -0.530 | -1.405 | 0.240 | 1.000 | 1.049 | 2.780 | 0.056 | 0.393 | 0 | 0 | 0 | flat_flat |
| hsa-miR-377* | -0.543 | 0.121 | -0.644 | -0.131 | 0.513 | 0.964 | 0.395 | 1.000 | -0.432 | -0.938 | 0.406 | 1.000 | 0.080 | 0.175 | 0.871 | 1.000 | 0 | 0 | 0 | flat_flat |
| hsa-miR-379 | -1.188 | -0.938 | -1.439 | -0.761 | 0.679 | 1.604 | 0.191 | 1.000 | -0.376 | -1.026 | 0.368 | 1.000 | 0.303 | 0.826 | 0.460 | 1.000 | 0 | 0 | 0 | flat_flat |
| hsa-miR-379* | -0.526 | -1.314 | -2.072 | -1.188 | 0.884 | 1.531 | 0.208 | 1.000 | -1.152 | -2.304 | 0.089 | 0.550 | -0.268 | -0.536 | 0.623 | 1.000 | 0 | 0 | 0 | flat_flat |
| hsa-miR-380 | -0.273 | -1.293 | -0.904 | 0.025 | 0.928 | 1.386 | 0.245 | 1.000 | -0.121 | -0.208 | 0.847 | 1.000 | 0.808 | 1.392 | 0.243 | 1.000 | 0 | 0 | 0 | flat_flat |
| hsa-miR-380* | -2.204 | -1.352 | -2.548 | -2.603 | -0.055 | -0.092 | 0.932 | 1.000 | -0.770 | -1.477 | 0.221 | 1.000 | -0.825 | -1.583 | 0.196 | 0.986 | 0 | 0 | 0 | flat_flat |
| hsa-miR-381 | 1.524 | 1.876 | 1.054 | 2.111 | 1.057 | 2.387 | 0.082 | 0.533 | -0.646 | -1.685 | 0.175 | 0.889 | 0.411 | 1.071 | 0.350 | 1.000 | 0 | 0 | 0 | flat_flat |
| hsa-miR-382 | 0.217 | 0.692 | 0.838 | -0.143 | -0.980 | -2.071 | 0.114 | 0.682 | 0.383 | 0.935 | 0.408 | 1.000 | -0.597 | -1.456 | 0.226 | 1.000 | 0 | 0 | 0 | flat_flat |
| hsa-miR-383 | -0.251 | 0.228 | 0.613 | -0.804 | -1.417 | -2.986 | 0.046 | 0.359 | 0.625 | 1.521 | 0.210 | 1.000 | -0.792 | -1.927 | 0.133 | 0.739 | 0 | 0 | 0 | flat_flat |
| hsa-miR-384 | -0.924 | -0.696 | -0.428 | -0.475 | -0.047 | -0.113 | 0.916 | 1.000 | 0.382 | 1.050 | 0.358 | 1.000 | 0.334 | 0.920 | 0.415 | 1.000 | 0 | 0 | 0 | flat_flat |
| hsa-miR-409-3p | -0.221 | -0.777 | -0.531 | 2.488 | 3.019 | 6.073 | 0.005 | 0.076 | -0.032 | -0.075 | 0.944 | 1.000 | 2.986 | 6.937 | 0.003 | 0.051 | 0 | 0 | 0 | flat_flat |
| hsa-miR-409-5p | -1.307 | -1.832 | -0.938 | -0.120 | 0.818 | 1.679 | 0.176 | 0.943 | 0.632 | 1.496 | 0.216 | 1.000 | 1.450 | 3.435 | 0.031 | 0.260 | 0 | 0 | 0 | flat_flat |
| hsa-miR-410 | -0.657 | -0.382 | -0.757 | -0.335 | 0.422 | 0.987 | 0.385 | 1.000 | -0.238 | -0.642 | 0.559 | 1.000 | 0.184 | 0.497 | 0.647 | 1.000 | 0 | 0 | 0 | flat_flat |
| hsa-miR-411 | -0.632 | -1.035 | -0.744 | -1.218 | -0.474 | -1.043 | 0.361 | 1.000 | 0.089 | 0.227 | 0.832 | 1.000 | -0.385 | -0.978 | 0.389 | 1.000 | 0 | 0 | 0 | flat_flat |
| hsa-miR-411* | 0.908 | 0.490 | -0.306 | -0.073 | 0.233 | 0.508 | 0.641 | 1.000 | -1.005 | -2.532 | 0.071 | 0.476 | -0.772 | -1.945 | 0.131 | 0.730 | 0 | 0 | 0 | flat_flat |
| hsa-miR-412 | -1.479 | -2.140 | -1.375 | -1.991 | -0.616 | -1.161 | 0.316 | 1.000 | 0.435 | 0.945 | 0.403 | 1.000 | -0.182 | -0.395 | 0.715 | 1.000 | 0 | 0 | 0 | flat_flat |
| hsa-miR-421 | 6.382 | 5.975 | 4.860 | 5.004 | 0.144 | 0.316 | 0.769 | 1.000 | -1.318 | -3.341 | 0.033 | 0.289 | -1.174 | -2.976 | 0.046 | 0.347 | 0 | 0 | 0 | flat_flat |
| hsa-miR-422a | -1.575 | -0.926 | -1.540 | -2.638 | -1.097 | -2.082 | 0.113 | 0.677 | -0.290 | -0.635 | 0.563 | 1.000 | -1.387 | -3.039 | 0.044 | 0.334 | 0 | 0 | 0 | flat_flat |
| hsa-miR-424* | -1.142 | -1.572 | 0.071 | -0.499 | -0.570 | -1.236 | 0.290 | 1.000 | 1.428 | 3.574 | 0.027 | 0.258 | 0.858 | 2.147 | 0.105 | 0.638 | 0 | 0 | 0 | flat_flat |
| hsa-miR-425 | 9.457 | 8.011 | 11.212 | 13.200 | 1.989 | 2.314 | 0.088 | 0.559 | 2.478 | 3.329 | 0.034 | 0.289 | 4.466 | 6.002 | 0.005 | 0.074 | 0 | 0 | 0 | flat_flat |
| hsa-miR-429 | 1.626 | 1.210 | -0.777 | -0.669 | 0.108 | 0.236 | 0.826 | 1.000 | -2.195 | -5.535 | 0.007 | 0.113 | -2.087 | -5.263 | 0.008 | 0.100 | 0 | 0 | 0 | flat_flat |
| hsa-miR-431 | -1.263 | -0.874 | -0.583 | -0.600 | -0.018 | -0.039 | 0.971 | 1.000 | 0.486 | 1.244 | 0.288 | 1.000 | 0.468 | 1.199 | 0.303 | 1.000 | 0 | 0 | 0 | flat_flat |
| hsa-miR-432* | 0.025 | -0.531 | -1.381 | -0.536 | 0.845 | 1.701 | 0.171 | 0.923 | -1.128 | -2.621 | 0.065 | 0.447 | -0.282 | -0.656 | 0.551 | 1.000 | 0 | 0 | 0 | flat_flat |
| hsa-miR-433 | -1.439 | -2.005 | -0.959 | -1.540 | -0.581 | -1.163 | 0.316 | 1.000 | 0.763 | 1.762 | 0.160 | 0.830 | 0.182 | 0.420 | 0.698 | 1.000 | 0 | 0 | 0 | flat_flat |
| hsa-miR-448 | -0.265 | 0.036 | -0.740 | -1.268 | -0.528 | -1.222 | 0.295 | 1.000 | -0.626 | -1.673 | 0.177 | 0.898 | -1.154 | -3.084 | 0.042 | 0.324 | 0 | 0 | 0 | flat_flat |
| hsa-miR-449a | 0.569 | -0.343 | 0.134 | -0.352 | -0.486 | -0.777 | 0.485 | 1.000 | 0.021 | 0.039 | 0.971 | 1.000 | -0.465 | -0.858 | 0.444 | 1.000 | 0 | 0 | 0 | flat_flat |
| hsa-miR-449b | 0.253 | -0.561 | 0.413 | -0.431 | -0.844 | -1.438 | 0.231 | 1.000 | 0.567 | 1.116 | 0.333 | 1.000 | -0.277 | -0.545 | 0.618 | 1.000 | 0 | 0 | 0 | flat_flat |
| hsa-miR-450a | 0.627 | 0.664 | 2.285 | 1.600 | -0.685 | -1.701 | 0.171 | 0.923 | 1.639 | 4.700 | 0.012 | 0.156 | 0.954 | 2.736 | 0.058 | 0.403 | 0 | 0 | 0 | flat_flat |
| hsa-miR-450b-3p | -0.478 | -0.352 | 0.097 | -0.637 | -0.734 | -1.801 | 0.153 | 0.855 | 0.512 | 1.451 | 0.227 | 1.000 | -0.222 | -0.629 | 0.567 | 1.000 | 0 | 0 | 0 | flat_flat |
| hsa-miR-450b-5p | -1.234 | -1.411 | -0.600 | -0.159 | 0.442 | 1.070 | 0.351 | 1.000 | 0.722 | 2.021 | 0.121 | 0.692 | 1.164 | 3.256 | 0.036 | 0.288 | 0 | 0 | 0 | flat_flat |
| hsa-miR-452* | -3.014 | -2.548 | -1.795 | -2.017 | -0.222 | -0.472 | 0.664 | 1.000 | 0.986 | 2.418 | 0.080 | 0.515 | 0.764 | 1.872 | 0.142 | 0.772 | 0 | 0 | 0 | flat_flat |
| hsa-miR-453 | -1.886 | -1.139 | -1.039 | -1.795 | -0.756 | -1.345 | 0.256 | 1.000 | 0.473 | 0.972 | 0.391 | 1.000 | -0.283 | -0.581 | 0.595 | 1.000 | 0 | 0 | 0 | flat_flat |
| hsa-miR-454* | 1.419 | 1.967 | 1.237 | 2.390 | 1.152 | 2.329 | 0.087 | 0.555 | -0.456 | -1.063 | 0.353 | 1.000 | 0.697 | 1.626 | 0.187 | 0.960 | 0 | 0 | 0 | flat_flat |
| hsa-miR-455-5p | 1.534 | 1.070 | 2.079 | -0.455 | -2.535 | -5.388 | 0.008 | 0.101 | 0.777 | 1.908 | 0.136 | 0.747 | -1.757 | -4.314 | 0.015 | 0.159 | 0 | 0 | 0 | flat_flat |
| hsa-miR-483-5p | 6.731 | 8.620 | 11.069 | 5.330 | -5.739 | -5.363 | 0.008 | 0.102 | 3.394 | 3.662 | 0.026 | 0.246 | -2.346 | -2.531 | 0.071 | 0.474 | 0 | 0 | 0 | flat_flat |
| hsa-miR-485-3p | 4.801 | 6.080 | 1.271 | 2.315 | 1.043 | 1.333 | 0.260 | 1.000 | -4.169 | -6.149 | 0.005 | 0.098 | -3.126 | -4.611 | 0.013 | 0.140 | 0 | 0 | 0 | flat_flat |
| hsa-miR-485-5p | -0.772 | -0.749 | -1.077 | -1.114 | -0.037 | -0.093 | 0.931 | 1.000 | -0.317 | -0.908 | 0.420 | 1.000 | -0.354 | -1.015 | 0.373 | 1.000 | 0 | 0 | 0 | flat_flat |
| hsa-miR-486-3p | -1.714 | -1.000 | -1.100 | 0.093 | 1.193 | 2.170 | 0.103 | 0.631 | 0.257 | 0.539 | 0.621 | 1.000 | 1.450 | 3.044 | 0.043 | 0.334 | 0 | 0 | 0 | flat_flat |
| hsa-miR-487a | -0.963 | -1.323 | -1.479 | 0.569 | 2.048 | 4.608 | 0.013 | 0.146 | -0.336 | -0.872 | 0.437 | 1.000 | 1.712 | 4.449 | 0.014 | 0.150 | 0 | 0 | 0 | flat_flat |
| hsa-miR-488 | 0.071 | -0.151 | 0.209 | -0.218 | -0.427 | -1.021 | 0.371 | 1.000 | 0.249 | 0.686 | 0.534 | 1.000 | -0.179 | -0.492 | 0.651 | 1.000 | 0 | 0 | 0 | flat_flat |
| hsa-miR-488* | -0.563 | -0.734 | -0.499 | -0.780 | -0.281 | -0.681 | 0.537 | 1.000 | 0.150 | 0.419 | 0.699 | 1.000 | -0.131 | -0.368 | 0.733 | 1.000 | 0 | 0 | 0 | flat_flat |
| hsa-miR-490-3p | -0.941 | -1.258 | -0.409 | -1.180 | -0.771 | -1.771 | 0.159 | 0.878 | 0.691 | 1.832 | 0.148 | 0.795 | -0.080 | -0.212 | 0.843 | 1.000 | 0 | 0 | 0 | flat_flat |
| hsa-miR-490-5p | -0.323 | -0.536 | -0.749 | -0.563 | 0.185 | 0.444 | 0.682 | 1.000 | -0.319 | -0.884 | 0.432 | 1.000 | -0.134 | -0.371 | 0.732 | 1.000 | 0 | 0 | 0 | flat_flat |
| hsa-miR-491-3p | 0.904 | 0.544 | 0.716 | 1.077 | 0.360 | 0.810 | 0.468 | 1.000 | -0.008 | -0.020 | 0.985 | 1.000 | 0.353 | 0.916 | 0.416 | 1.000 | 0 | 0 | 0 | flat_flat |
| hsa-miR-491-5p | -0.485 | -0.872 | -0.563 | -0.946 | -0.383 | -0.850 | 0.448 | 1.000 | 0.116 | 0.297 | 0.783 | 1.000 | -0.267 | -0.685 | 0.535 | 1.000 | 0 | 0 | 0 | flat_flat |
| hsa-miR-492 | -0.114 | -0.632 | 0.980 | 0.011 | -0.968 | -1.994 | 0.124 | 0.731 | 1.352 | 3.215 | 0.037 | 0.300 | 0.384 | 0.913 | 0.418 | 1.000 | 0 | 0 | 0 | flat_flat |
| hsa-miR-493 | -1.073 | -0.228 | -1.763 | -2.081 | -0.317 | -0.529 | 0.627 | 1.000 | -1.113 | -2.145 | 0.106 | 0.627 | -1.430 | -2.757 | 0.057 | 0.397 | 0 | 0 | 0 | flat_flat |
| hsa-miR-493* | -0.610 | -0.821 | -0.443 | 0.099 | 0.542 | 1.298 | 0.271 | 1.000 | 0.273 | 0.755 | 0.496 | 1.000 | 0.815 | 2.254 | 0.094 | 0.582 | 0 | 0 | 0 | flat_flat |
| hsa-miR-494 | 12.638 | 12.866 | 12.055 | 12.612 | 0.557 | 1.326 | 0.262 | 1.000 | -0.697 | -1.918 | 0.135 | 0.744 | -0.140 | -0.386 | 0.721 | 1.000 | 0 | 0 | 0 | flat_flat |
| hsa-miR-495 | 0.050 | -0.041 | -0.526 | 0.678 | 1.204 | 2.973 | 0.046 | 0.360 | -0.531 | -1.513 | 0.212 | 1.000 | 0.674 | 1.920 | 0.135 | 0.742 | 0 | 0 | 0 | flat_flat |
| hsa-miR-496 | -0.428 | 0.449 | -1.591 | -1.016 | 0.576 | 0.942 | 0.405 | 1.000 | -1.602 | -3.025 | 0.044 | 0.342 | -1.026 | -1.938 | 0.132 | 0.734 | 0 | 0 | 0 | flat_flat |
| hsa-miR-497* | -2.375 | -1.825 | -1.527 | -0.610 | 0.917 | 1.852 | 0.145 | 0.823 | 0.573 | 1.337 | 0.259 | 1.000 | 1.490 | 3.475 | 0.030 | 0.256 | 0 | 0 | 0 | flat_flat |
| hsa-miR-499-3p | -1.975 | -2.238 | -2.512 | -2.041 | 0.471 | 1.107 | 0.336 | 1.000 | -0.405 | -1.101 | 0.339 | 1.000 | 0.065 | 0.177 | 0.869 | 1.000 | 0 | 0 | 0 | flat_flat |
| hsa-miR-499-5p | -0.892 | -0.807 | -0.625 | 0.017 | 0.642 | 1.587 | 0.195 | 1.000 | 0.225 | 0.642 | 0.559 | 1.000 | 0.867 | 2.474 | 0.075 | 0.492 | 0 | 0 | 0 | flat_flat |
| hsa-miR-500 | 3.465 | 7.270 | 1.926 | 10.862 | 8.936 | 4.384 | 0.015 | 0.161 | -3.442 | -1.950 | 0.130 | 0.732 | 5.494 | 3.113 | 0.041 | 0.317 | 0 | 0 | 0 | flat_flat |
| hsa-miR-501-3p | -0.477 | -0.740 | 1.967 | -0.036 | -2.003 | -4.708 | 0.012 | 0.140 | 2.576 | 6.992 | 0.003 | 0.073 | 0.573 | 1.555 | 0.202 | 1.000 | 0 | 0 | 0 | flat_flat |
| hsa-miR-501-5p | 3.381 | 10.730 | 4.358 | 9.178 | 4.821 | 1.242 | 0.288 | 1.000 | -2.698 | -0.803 | 0.471 | 1.000 | 2.123 | 0.632 | 0.565 | 1.000 | 0 | 0 | 0 | flat_flat |
| hsa-miR-504 | -0.684 | -2.463 | 0.004 | 0.275 | 0.270 | 0.266 | 0.805 | 1.000 | 1.578 | 1.791 | 0.155 | 0.816 | 1.848 | 2.099 | 0.111 | 0.656 | 0 | 0 | 0 | flat_flat |
| hsa-miR-505 | 6.437 | 5.233 | 7.925 | 6.437 | -1.488 | -1.986 | 0.125 | 0.735 | 2.090 | 3.221 | 0.037 | 0.300 | 0.602 | 0.928 | 0.411 | 1.000 | 0 | 0 | 0 | flat_flat |
| hsa-miR-505* | 4.566 | 2.706 | 5.438 | 1.588 | -3.850 | -3.644 | 0.026 | 0.238 | 1.803 | 1.970 | 0.127 | 0.718 | -2.047 | -2.238 | 0.096 | 0.588 | 0 | 0 | 0 | flat_flat |
| hsa-miR-506 | -0.302 | 0.359 | 0.275 | 1.857 | 1.583 | 2.978 | 0.046 | 0.360 | 0.246 | 0.534 | 0.624 | 1.000 | 1.828 | 3.974 | 0.020 | 0.189 | 0 | 0 | 0 | flat_flat |
| hsa-miR-507 | -0.986 | -0.446 | 0.449 | -0.744 | -1.193 | -2.422 | 0.079 | 0.521 | 1.164 | 2.731 | 0.058 | 0.415 | -0.028 | -0.066 | 0.951 | 1.000 | 0 | 0 | 0 | flat_flat |
| hsa-miR-508-3p | -0.931 | -1.124 | -0.836 | 1.367 | 2.203 | 5.311 | 0.008 | 0.105 | 0.191 | 0.532 | 0.625 | 1.000 | 2.394 | 6.665 | 0.004 | 0.057 | 0 | 0 | 0 | flat_flat |
| hsa-miR-508-5p | -1.298 | -1.403 | -0.729 | -1.215 | -0.485 | -1.195 | 0.304 | 1.000 | 0.621 | 1.767 | 0.159 | 0.828 | 0.136 | 0.387 | 0.720 | 1.000 | 0 | 0 | 0 | flat_flat |
| hsa-miR-509-3-5p | -1.348 | -1.100 | -1.245 | -1.331 | -0.086 | -0.203 | 0.850 | 1.000 | -0.021 | -0.056 | 0.958 | 1.000 | -0.107 | -0.291 | 0.787 | 1.000 | 0 | 0 | 0 | flat_flat |
| hsa-miR-509-3p | -1.278 | -1.891 | 0.121 | -0.958 | -1.079 | -2.093 | 0.112 | 0.670 | 1.705 | 3.821 | 0.022 | 0.229 | 0.627 | 1.404 | 0.240 | 1.000 | 0 | 0 | 0 | flat_flat |
| hsa-miR-509-5p | -0.950 | -1.039 | -0.572 | -0.664 | -0.093 | -0.229 | 0.831 | 1.000 | 0.423 | 1.206 | 0.301 | 1.000 | 0.330 | 0.942 | 0.405 | 1.000 | 0 | 0 | 0 | flat_flat |
| hsa-miR-510 | -1.000 | -0.368 | 0.033 | -1.423 | -1.456 | -2.792 | 0.055 | 0.407 | 0.717 | 1.588 | 0.195 | 0.967 | -0.739 | -1.636 | 0.184 | 0.954 | 0 | 0 | 0 | flat_flat |
| hsa-miR-511 | -0.664 | -1.346 | -1.146 | -0.356 | 0.790 | 1.468 | 0.223 | 1.000 | -0.141 | -0.302 | 0.779 | 1.000 | 0.650 | 1.392 | 0.243 | 1.000 | 0 | 0 | 0 | flat_flat |
| hsa-miR-512-5p | 4.348 | 3.153 | -0.424 | 0.050 | 0.474 | 0.635 | 0.563 | 1.000 | -4.174 | -6.466 | 0.004 | 0.088 | -3.701 | -5.732 | 0.006 | 0.082 | 0 | 0 | 0 | flat_flat |
| hsa-miR-513a-3p | -1.415 | -1.651 | -0.764 | 0.054 | 0.818 | 1.944 | 0.131 | 0.761 | 0.769 | 2.110 | 0.110 | 0.645 | 1.587 | 4.355 | 0.015 | 0.158 | 0 | 0 | 0 | flat_flat |
| hsa-miR-513b | 0.645 | -0.079 | 0.388 | 2.243 | 1.855 | 3.353 | 0.033 | 0.281 | 0.105 | 0.218 | 0.839 | 1.000 | 1.960 | 4.090 | 0.018 | 0.180 | 0 | 0 | 0 | flat_flat |
| hsa-miR-513c | 0.176 | -0.218 | -0.120 | 0.585 | 0.705 | 1.558 | 0.201 | 1.000 | -0.099 | -0.252 | 0.815 | 1.000 | 0.606 | 1.548 | 0.204 | 1.000 | 0 | 0 | 0 | flat_flat |
| hsa-miR-514 | -1.654 | -0.424 | -0.478 | -0.670 | -0.191 | -0.251 | 0.815 | 1.000 | 0.561 | 0.850 | 0.448 | 1.000 | 0.369 | 0.560 | 0.608 | 1.000 | 0 | 0 | 0 | flat_flat |
| hsa-miR-516a-3p | -0.071 | -0.657 | -0.790 | -0.182 | 0.609 | 1.203 | 0.302 | 1.000 | -0.426 | -0.973 | 0.391 | 1.000 | 0.182 | 0.416 | 0.701 | 1.000 | 0 | 0 | 0 | flat_flat |
| hsa-miR-517* | -0.320 | -0.477 | -0.577 | -0.680 | -0.104 | -0.252 | 0.814 | 1.000 | -0.178 | -0.502 | 0.645 | 1.000 | -0.282 | -0.793 | 0.476 | 1.000 | 0 | 0 | 0 | flat_flat |
| hsa-miR-517c | 2.303 | 2.079 | 0.086 | 0.501 | 0.414 | 0.989 | 0.384 | 1.000 | -2.105 | -5.800 | 0.006 | 0.106 | -1.691 | -4.658 | 0.012 | 0.136 | 0 | 0 | 0 | flat_flat |
| hsa-miR-518a-3p | 1.701 | 0.980 | -0.389 | 0.188 | 0.578 | 1.046 | 0.360 | 1.000 | -1.730 | -3.614 | 0.027 | 0.251 | -1.152 | -2.407 | 0.080 | 0.516 | 0 | 0 | 0 | flat_flat |
| hsa-miR-518a-5p | -0.368 | -1.092 | 0.011 | -0.905 | -0.916 | -1.655 | 0.181 | 0.960 | 0.742 | 1.547 | 0.204 | 1.000 | -0.175 | -0.364 | 0.736 | 1.000 | 0 | 0 | 0 | flat_flat |
| hsa-miR-518c | 1.545 | 0.950 | 0.367 | -0.015 | -0.382 | -0.750 | 0.499 | 1.000 | -0.880 | -1.997 | 0.124 | 0.702 | -1.262 | -2.862 | 0.051 | 0.371 | 0 | 0 | 0 | flat_flat |
| hsa-miR-518c* | 2.285 | 1.672 | 0.359 | -0.383 | -0.742 | -1.442 | 0.230 | 1.000 | -1.619 | -3.631 | 0.026 | 0.250 | -2.362 | -5.296 | 0.008 | 0.099 | 0 | 0 | 0 | flat_flat |
| hsa-miR-518d-3p | 0.585 | 1.043 | -0.153 | 1.210 | 1.363 | 2.909 | 0.049 | 0.372 | -0.967 | -2.384 | 0.082 | 0.523 | 0.396 | 0.976 | 0.390 | 1.000 | 0 | 0 | 0 | flat_flat |
| hsa-miR-518e | 1.769 | 1.201 | -0.280 | -0.012 | 0.268 | 0.535 | 0.624 | 1.000 | -1.765 | -4.071 | 0.019 | 0.200 | -1.497 | -3.453 | 0.030 | 0.257 | 0 | 0 | 0 | flat_flat |
| hsa-miR-518f | -0.147 | -0.232 | -1.005 | -0.509 | 0.496 | 1.226 | 0.294 | 1.000 | -0.816 | -2.327 | 0.087 | 0.542 | -0.319 | -0.911 | 0.419 | 1.000 | 0 | 0 | 0 | flat_flat |
| hsa-miR-518f* | 0.099 | 0.109 | -0.394 | -0.734 | -0.341 | -0.847 | 0.449 | 1.000 | -0.498 | -1.429 | 0.233 | 1.000 | -0.839 | -2.407 | 0.080 | 0.516 | 0 | 0 | 0 | flat_flat |
| hsa-miR-519c-3p | 6.501 | 4.801 | 1.025 | 0.169 | -0.856 | -0.874 | 0.436 | 1.000 | -4.627 | -5.455 | 0.007 | 0.117 | -5.482 | -6.464 | 0.004 | 0.061 | 0 | 0 | 0 | flat_flat |
| hsa-miR-519e | 0.789 | 0.972 | 0.234 | 0.791 | 0.557 | 1.347 | 0.256 | 1.000 | -0.646 | -1.803 | 0.153 | 0.814 | -0.089 | -0.248 | 0.818 | 1.000 | 0 | 0 | 0 | flat_flat |
| hsa-miR-519e* | 1.445 | 1.012 | 0.109 | -0.188 | -0.298 | -0.644 | 0.558 | 1.000 | -1.119 | -2.796 | 0.055 | 0.399 | -1.417 | -3.540 | 0.028 | 0.247 | 0 | 0 | 0 | flat_flat |
| hsa-miR-520a-3p | 0.875 | 0.634 | 0.598 | 0.688 | 0.090 | 0.214 | 0.842 | 1.000 | -0.157 | -0.430 | 0.691 | 1.000 | -0.067 | -0.183 | 0.865 | 1.000 | 0 | 0 | 0 | flat_flat |
| hsa-miR-520b | 3.771 | 3.487 | 1.672 | 1.125 | -0.547 | -1.276 | 0.277 | 1.000 | -1.957 | -5.268 | 0.008 | 0.124 | -2.504 | -6.742 | 0.004 | 0.055 | 0 | 0 | 0 | flat_flat |
| hsa-miR-520d-3p | 2.172 | 2.453 | 1.419 | 1.524 | 0.105 | 0.246 | 0.819 | 1.000 | -0.894 | -2.408 | 0.080 | 0.517 | -0.788 | -2.124 | 0.108 | 0.647 | 0 | 0 | 0 | flat_flat |
| hsa-miR-520e | 1.070 | 1.153 | 1.556 | 2.123 | 0.567 | 1.402 | 0.240 | 1.000 | 0.444 | 1.267 | 0.280 | 1.000 | 1.012 | 2.887 | 0.050 | 0.369 | 0 | 0 | 0 | flat_flat |
| hsa-miR-520g | 5.004 | 4.105 | 0.569 | 1.099 | 0.530 | 0.855 | 0.446 | 1.000 | -3.985 | -7.417 | 0.003 | 0.066 | -3.455 | -6.431 | 0.004 | 0.061 | 0 | 0 | 0 | flat_flat |
| hsa-miR-520h | 3.544 | 1.830 | -0.015 | 0.280 | 0.294 | 0.299 | 0.782 | 1.000 | -2.702 | -3.164 | 0.039 | 0.307 | -2.408 | -2.819 | 0.054 | 0.381 | 0 | 0 | 0 | flat_flat |
| hsa-miR-521 | 3.016 | 2.123 | 1.229 | 1.445 | 0.217 | 0.351 | 0.745 | 1.000 | -1.341 | -2.506 | 0.073 | 0.485 | -1.124 | -2.101 | 0.111 | 0.655 | 0 | 0 | 0 | flat_flat |
| hsa-miR-522 | 1.635 | 1.419 | 0.303 | -0.267 | -0.570 | -1.365 | 0.251 | 1.000 | -1.224 | -3.381 | 0.032 | 0.284 | -1.794 | -4.957 | 0.010 | 0.117 | 0 | 0 | 0 | flat_flat |
| hsa-miR-523 | -0.591 | -1.031 | -0.882 | -0.691 | 0.191 | 0.412 | 0.704 | 1.000 | -0.071 | -0.177 | 0.869 | 1.000 | 0.120 | 0.299 | 0.782 | 1.000 | 0 | 0 | 0 | flat_flat |
| hsa-miR-524-3p | 0.557 | 0.286 | 0.350 | -0.096 | -0.445 | -1.044 | 0.361 | 1.000 | -0.072 | -0.195 | 0.856 | 1.000 | -0.517 | -1.399 | 0.241 | 1.000 | 0 | 0 | 0 | flat_flat |
| hsa-miR-525-3p | 0.006 | -0.309 | -1.000 | 0.006 | 1.006 | 2.313 | 0.089 | 0.559 | -0.849 | -2.252 | 0.094 | 0.575 | 0.158 | 0.419 | 0.699 | 1.000 | 0 | 0 | 0 | flat_flat |
| hsa-miR-526b* | 0.375 | 0.553 | 0.188 | 0.176 | -0.012 | -0.030 | 0.978 | 1.000 | -0.275 | -0.770 | 0.488 | 1.000 | -0.288 | -0.805 | 0.470 | 1.000 | 0 | 0 | 0 | flat_flat |
| hsa-miR-539 | 1.185 | 1.588 | 0.076 | 0.243 | 0.167 | 0.367 | 0.734 | 1.000 | -1.311 | -3.330 | 0.034 | 0.289 | -1.144 | -2.906 | 0.049 | 0.366 | 0 | 0 | 0 | flat_flat |
| hsa-miR-541 | -1.804 | -1.508 | -2.414 | -1.628 | 0.786 | 1.822 | 0.150 | 0.837 | -0.758 | -2.029 | 0.120 | 0.688 | 0.028 | 0.075 | 0.944 | 1.000 | 0 | 0 | 0 | flat_flat |
| hsa-miR-541* | -1.134 | -1.040 | -0.163 | -0.253 | -0.090 | -0.223 | 0.836 | 1.000 | 0.924 | 2.633 | 0.064 | 0.444 | 0.834 | 2.375 | 0.083 | 0.526 | 0 | 0 | 0 | flat_flat |
| hsa-miR-542-3p | 1.683 | 0.478 | 3.296 | -1.426 | -4.723 | -6.298 | 0.004 | 0.069 | 2.216 | 3.412 | 0.031 | 0.282 | -2.507 | -3.861 | 0.022 | 0.202 | 0 | 0 | 0 | flat_flat |
| hsa-miR-542-5p | 0.807 | 2.172 | 1.990 | 2.303 | 0.313 | 0.381 | 0.724 | 1.000 | 0.501 | 0.704 | 0.524 | 1.000 | 0.814 | 1.144 | 0.323 | 1.000 | 0 | 0 | 0 | flat_flat |
| hsa-miR-543 | 0.037 | -0.504 | -0.228 | 0.775 | 1.003 | 2.037 | 0.119 | 0.704 | 0.006 | 0.013 | 0.990 | 1.000 | 1.009 | 2.365 | 0.084 | 0.530 | 0 | 0 | 0 | flat_flat |
| hsa-miR-544 | -0.615 | -0.580 | -0.494 | 0.908 | 1.402 | 3.482 | 0.030 | 0.262 | 0.103 | 0.295 | 0.784 | 1.000 | 1.505 | 4.315 | 0.015 | 0.159 | 0 | 0 | 0 | flat_flat |
| hsa-miR-545* | -2.064 | -1.286 | -0.734 | -1.283 | -0.549 | -0.957 | 0.398 | 1.000 | 0.940 | 1.894 | 0.138 | 0.754 | 0.392 | 0.789 | 0.479 | 1.000 | 0 | 0 | 0 | flat_flat |
| hsa-miR-548a-3p | -0.597 | -0.959 | -1.722 | 1.399 | 3.121 | 7.016 | 0.003 | 0.056 | -0.944 | -2.450 | 0.077 | 0.505 | 2.177 | 5.651 | 0.006 | 0.085 | 0 | 0 | 0 | flat_flat |
| hsa-miR-548a-5p | -0.704 | -0.356 | -0.610 | 0.917 | 1.527 | 3.455 | 0.030 | 0.262 | -0.080 | -0.208 | 0.846 | 1.000 | 1.447 | 3.782 | 0.023 | 0.214 | 0 | 0 | 0 | flat_flat |
| hsa-miR-548b-3p | -0.225 | -0.335 | 0.070 | 0.729 | 0.660 | 1.623 | 0.187 | 0.989 | 0.350 | 0.993 | 0.382 | 1.000 | 1.009 | 2.868 | 0.051 | 0.370 | 0 | 0 | 0 | flat_flat |
| hsa-miR-548b-5p | -0.280 | -0.302 | 1.327 | 0.423 | -0.904 | -2.247 | 0.095 | 0.592 | 1.618 | 4.643 | 0.012 | 0.159 | 0.714 | 2.049 | 0.117 | 0.678 | 0 | 0 | 0 | flat_flat |
| hsa-miR-548c-3p | 0.721 | 1.086 | -0.680 | 1.335 | 2.015 | 4.523 | 0.013 | 0.151 | -1.584 | -4.104 | 0.018 | 0.200 | 0.431 | 1.118 | 0.332 | 1.000 | 0 | 0 | 0 | flat_flat |
| hsa-miR-548c-5p | 1.173 | 0.501 | 1.800 | 0.418 | -1.382 | -2.582 | 0.067 | 0.462 | 0.963 | 2.078 | 0.113 | 0.659 | -0.419 | -0.903 | 0.423 | 1.000 | 0 | 0 | 0 | flat_flat |
| hsa-miR-548d-3p | -0.838 | -0.329 | -0.273 | 0.213 | 0.486 | 1.006 | 0.377 | 1.000 | 0.311 | 0.742 | 0.503 | 1.000 | 0.797 | 1.904 | 0.137 | 0.753 | 0 | 0 | 0 | flat_flat |
| hsa-miR-548d-5p | 1.012 | 1.099 | 1.388 | 0.367 | -1.021 | -2.523 | 0.072 | 0.477 | 0.333 | 0.949 | 0.401 | 1.000 | -0.689 | -1.964 | 0.128 | 0.718 | 0 | 0 | 0 | flat_flat |
| hsa-miR-548e | -0.740 | -0.623 | -0.477 | 0.838 | 1.314 | 3.230 | 0.037 | 0.304 | 0.205 | 0.582 | 0.595 | 1.000 | 1.519 | 4.311 | 0.015 | 0.159 | 0 | 0 | 0 | flat_flat |
| hsa-miR-548f | -0.363 | 0.365 | -1.314 | -0.485 | 0.829 | 1.493 | 0.217 | 1.000 | -1.315 | -2.735 | 0.058 | 0.414 | -0.486 | -1.011 | 0.374 | 1.000 | 0 | 0 | 0 | flat_flat |
| hsa-miR-548g | -0.676 | 0.076 | -1.445 | 0.148 | 1.593 | 2.825 | 0.053 | 0.399 | -1.145 | -2.345 | 0.086 | 0.535 | 0.448 | 0.917 | 0.416 | 1.000 | 0 | 0 | 0 | flat_flat |
| hsa-miR-548h | -1.559 | -0.924 | -0.293 | -1.108 | -0.815 | -1.560 | 0.201 | 1.000 | 0.948 | 2.096 | 0.111 | 0.651 | 0.133 | 0.295 | 0.784 | 1.000 | 0 | 0 | 0 | flat_flat |
| hsa-miR-548i | -0.821 | -0.539 | -0.798 | -0.009 | 0.789 | 1.840 | 0.147 | 0.830 | -0.118 | -0.317 | 0.768 | 1.000 | 0.671 | 1.807 | 0.152 | 0.814 | 0 | 0 | 0 | flat_flat |
| hsa-miR-548j | -1.449 | -1.156 | 0.153 | 0.076 | -0.077 | -0.178 | 0.868 | 1.000 | 1.456 | 3.903 | 0.021 | 0.220 | 1.379 | 3.697 | 0.025 | 0.225 | 0 | 0 | 0 | flat_flat |
| hsa-miR-548k | -0.625 | -0.669 | -0.404 | 0.109 | 0.513 | 1.274 | 0.278 | 1.000 | 0.243 | 0.696 | 0.529 | 1.000 | 0.756 | 2.167 | 0.103 | 0.626 | 0 | 0 | 0 | flat_flat |
| hsa-miR-548l | -1.215 | -1.302 | -0.343 | 0.139 | 0.482 | 1.190 | 0.306 | 1.000 | 0.916 | 2.612 | 0.065 | 0.450 | 1.397 | 3.985 | 0.020 | 0.189 | 0 | 0 | 0 | flat_flat |
| hsa-miR-548m | -0.448 | -0.066 | 1.435 | 0.222 | -1.213 | -2.698 | 0.060 | 0.427 | 1.692 | 4.347 | 0.015 | 0.179 | 0.479 | 1.231 | 0.292 | 1.000 | 0 | 0 | 0 | flat_flat |
| hsa-miR-548n | -1.635 | -0.919 | -0.874 | -0.696 | 0.179 | 0.324 | 0.764 | 1.000 | 0.403 | 0.844 | 0.451 | 1.000 | 0.581 | 1.219 | 0.296 | 1.000 | 0 | 0 | 0 | flat_flat |
| hsa-miR-548o | -0.965 | -1.057 | -0.545 | -0.002 | 0.543 | 1.341 | 0.258 | 1.000 | 0.466 | 1.327 | 0.262 | 1.000 | 1.009 | 2.876 | 0.051 | 0.370 | 0 | 0 | 0 | flat_flat |
| hsa-miR-548p | -1.124 | -1.443 | -1.447 | -1.003 | 0.444 | 1.019 | 0.371 | 1.000 | -0.164 | -0.434 | 0.689 | 1.000 | 0.280 | 0.743 | 0.503 | 1.000 | 0 | 0 | 0 | flat_flat |
| hsa-miR-549 | -1.508 | -1.160 | -2.574 | -1.392 | 1.183 | 2.678 | 0.061 | 0.430 | -1.241 | -3.243 | 0.036 | 0.300 | -0.058 | -0.151 | 0.888 | 1.000 | 0 | 0 | 0 | flat_flat |
| hsa-miR-551a | -0.549 | -0.051 | -1.897 | 0.313 | 2.210 | 4.606 | 0.013 | 0.146 | -1.597 | -3.843 | 0.022 | 0.227 | 0.613 | 1.476 | 0.221 | 1.000 | 0 | 0 | 0 | flat_flat |
| hsa-miR-551b* | -1.752 | 0.093 | -1.504 | -2.729 | -1.224 | -1.168 | 0.314 | 1.000 | -0.675 | -0.743 | 0.503 | 1.000 | -1.899 | -2.091 | 0.112 | 0.657 | 0 | 0 | 0 | flat_flat |
| hsa-miR-552 | -1.800 | -1.003 | -0.965 | -1.357 | -0.392 | -0.676 | 0.540 | 1.000 | 0.437 | 0.869 | 0.439 | 1.000 | 0.044 | 0.088 | 0.935 | 1.000 | 0 | 0 | 0 | flat_flat |
| hsa-miR-553 | -0.600 | -0.913 | -0.371 | -0.153 | 0.217 | 0.501 | 0.645 | 1.000 | 0.386 | 1.025 | 0.369 | 1.000 | 0.603 | 1.603 | 0.191 | 0.974 | 0 | 0 | 0 | flat_flat |
| hsa-miR-554 | -1.426 | -1.218 | -2.463 | -1.879 | 0.584 | 1.401 | 0.241 | 1.000 | -1.140 | -3.159 | 0.039 | 0.307 | -0.556 | -1.542 | 0.205 | 1.000 | 0 | 0 | 0 | flat_flat |
| hsa-miR-555 | -1.139 | -1.180 | -1.587 | -1.057 | 0.530 | 1.316 | 0.265 | 1.000 | -0.427 | -1.225 | 0.294 | 1.000 | 0.103 | 0.294 | 0.785 | 1.000 | 0 | 0 | 0 | flat_flat |
| hsa-miR-556-3p | -0.049 | -0.071 | -0.416 | 1.110 | 1.527 | 3.794 | 0.023 | 0.216 | -0.356 | -1.023 | 0.370 | 1.000 | 1.170 | 3.358 | 0.033 | 0.275 | 0 | 0 | 0 | flat_flat |
| hsa-miR-556-5p | -2.150 | -1.447 | -1.667 | -1.479 | 0.188 | 0.344 | 0.750 | 1.000 | 0.132 | 0.279 | 0.796 | 1.000 | 0.319 | 0.675 | 0.540 | 1.000 | 0 | 0 | 0 | flat_flat |
| hsa-miR-557 | 1.210 | 1.701 | 3.907 | 1.489 | -2.419 | -5.062 | 0.009 | 0.117 | 2.452 | 5.925 | 0.006 | 0.102 | 0.033 | 0.080 | 0.941 | 1.000 | 0 | 0 | 0 | flat_flat |
| hsa-miR-558 | -2.309 | -0.953 | -1.462 | -1.651 | -0.189 | -0.232 | 0.829 | 1.000 | 0.169 | 0.239 | 0.824 | 1.000 | -0.020 | -0.028 | 0.979 | 1.000 | 0 | 0 | 0 | flat_flat |
| hsa-miR-559 | -2.238 | -2.216 | -2.375 | -1.587 | 0.788 | 1.959 | 0.129 | 0.753 | -0.148 | -0.424 | 0.695 | 1.000 | 0.641 | 1.838 | 0.147 | 0.790 | 0 | 0 | 0 | flat_flat |
| hsa-miR-561 | -2.099 | -1.492 | -0.670 | -0.539 | 0.131 | 0.255 | 0.813 | 1.000 | 1.126 | 2.533 | 0.071 | 0.476 | 1.257 | 2.828 | 0.053 | 0.379 | 0 | 0 | 0 | flat_flat |
| hsa-miR-562 | -0.725 | -1.234 | -0.812 | -0.204 | 0.608 | 1.259 | 0.283 | 1.000 | 0.168 | 0.401 | 0.711 | 1.000 | 0.776 | 1.855 | 0.145 | 0.782 | 0 | 0 | 0 | flat_flat |
| hsa-miR-563 | 0.985 | 0.251 | 1.588 | 3.771 | 2.183 | 3.919 | 0.021 | 0.203 | 0.971 | 2.012 | 0.122 | 0.694 | 3.153 | 6.537 | 0.004 | 0.059 | 0 | 0 | 0 | flat_flat |
| hsa-miR-566 | 0.912 | 0.858 | -1.124 | -0.412 | 0.712 | 1.764 | 0.160 | 0.879 | -2.009 | -5.752 | 0.006 | 0.108 | -1.297 | -3.714 | 0.024 | 0.223 | 0 | 0 | 0 | flat_flat |
| hsa-miR-567 | -0.577 | -0.275 | -1.635 | -1.286 | 0.349 | 0.808 | 0.469 | 1.000 | -1.209 | -3.230 | 0.037 | 0.300 | -0.860 | -2.297 | 0.090 | 0.561 | 0 | 0 | 0 | flat_flat |
| hsa-miR-568 | -0.867 | -0.941 | -0.580 | -0.817 | -0.237 | -0.588 | 0.591 | 1.000 | 0.324 | 0.925 | 0.412 | 1.000 | 0.086 | 0.247 | 0.818 | 1.000 | 0 | 0 | 0 | flat_flat |
| hsa-miR-569 | -1.423 | -1.939 | -1.331 | -1.939 | -0.608 | -1.254 | 0.285 | 1.000 | 0.350 | 0.833 | 0.456 | 1.000 | -0.258 | -0.614 | 0.575 | 1.000 | 0 | 0 | 0 | flat_flat |
| hsa-miR-570 | -0.436 | -0.288 | -0.285 | -0.051 | 0.234 | 0.571 | 0.601 | 1.000 | 0.077 | 0.217 | 0.840 | 1.000 | 0.311 | 0.876 | 0.435 | 1.000 | 0 | 0 | 0 | flat_flat |
| hsa-miR-571 | -1.064 | -1.364 | -0.460 | -0.757 | -0.297 | -0.688 | 0.533 | 1.000 | 0.754 | 2.015 | 0.121 | 0.694 | 0.457 | 1.221 | 0.295 | 1.000 | 0 | 0 | 0 | flat_flat |
| hsa-miR-572 | 8.550 | 9.962 | 8.516 | 6.300 | -2.216 | -2.627 | 0.065 | 0.445 | -0.740 | -1.013 | 0.374 | 1.000 | -2.956 | -4.046 | 0.019 | 0.184 | 0 | 0 | 0 | flat_flat |
| hsa-miR-573 | -2.360 | -2.512 | -1.234 | -1.891 | -0.657 | -1.601 | 0.192 | 1.000 | 1.202 | 3.384 | 0.032 | 0.284 | 0.545 | 1.535 | 0.207 | 1.000 | 0 | 0 | 0 | flat_flat |
| hsa-miR-574-3p | 13.272 | 13.354 | 12.067 | 13.749 | 1.683 | 4.159 | 0.017 | 0.177 | -1.246 | -3.557 | 0.028 | 0.260 | 0.437 | 1.246 | 0.287 | 1.000 | 0 | 0 | 0 | flat_flat |
| hsa-miR-574-5p | 13.354 | 13.260 | 11.170 | 13.544 | 2.374 | 5.857 | 0.006 | 0.083 | -2.137 | -6.089 | 0.005 | 0.099 | 0.237 | 0.675 | 0.540 | 1.000 | 0 | 0 | 0 | flat_flat |
| hsa-miR-576-3p | -1.168 | 0.006 | -0.877 | -1.862 | -0.985 | -1.338 | 0.259 | 1.000 | -0.296 | -0.464 | 0.669 | 1.000 | -1.281 | -2.010 | 0.122 | 0.691 | 0 | 0 | 0 | flat_flat |
| hsa-miR-576-5p | 0.775 | 0.354 | 0.999 | 2.172 | 1.172 | 2.555 | 0.069 | 0.471 | 0.434 | 1.093 | 0.342 | 1.000 | 1.607 | 4.043 | 0.019 | 0.184 | 0 | 0 | 0 | flat_flat |
| hsa-miR-577 | -1.314 | -1.064 | -0.461 | -0.867 | -0.405 | -0.957 | 0.398 | 1.000 | 0.728 | 1.985 | 0.125 | 0.710 | 0.322 | 0.880 | 0.434 | 1.000 | 0 | 0 | 0 | flat_flat |
| hsa-miR-578 | -1.492 | -1.628 | -0.879 | -0.648 | 0.230 | 0.564 | 0.606 | 1.000 | 0.681 | 1.926 | 0.134 | 0.742 | 0.912 | 2.577 | 0.068 | 0.457 | 0 | 0 | 0 | flat_flat |
| hsa-miR-579 | -1.963 | -2.264 | -1.434 | -1.546 | -0.112 | -0.260 | 0.809 | 1.000 | 0.679 | 1.814 | 0.151 | 0.806 | 0.567 | 1.514 | 0.212 | 1.000 | 0 | 0 | 0 | flat_flat |
| hsa-miR-580 | -2.574 | -2.505 | -1.387 | -1.907 | -0.520 | -1.288 | 0.274 | 1.000 | 1.153 | 3.296 | 0.035 | 0.295 | 0.633 | 1.808 | 0.152 | 0.814 | 0 | 0 | 0 | flat_flat |
| hsa-miR-581 | -1.125 | -0.804 | -1.814 | -0.615 | 1.200 | 2.750 | 0.057 | 0.420 | -0.850 | -2.249 | 0.095 | 0.575 | 0.350 | 0.926 | 0.412 | 1.000 | 0 | 0 | 0 | flat_flat |
| hsa-miR-582-3p | -1.370 | -1.007 | -1.891 | -0.986 | 0.905 | 2.032 | 0.119 | 0.706 | -0.702 | -1.822 | 0.150 | 0.801 | 0.202 | 0.525 | 0.630 | 1.000 | 0 | 0 | 0 | flat_flat |
| hsa-miR-583 | 0.213 | 0.775 | -1.514 | -0.710 | 0.804 | 1.612 | 0.190 | 0.998 | -2.009 | -4.648 | 0.012 | 0.159 | -1.204 | -2.787 | 0.055 | 0.391 | 0 | 0 | 0 | flat_flat |
| hsa-miR-584 | -0.126 | 0.280 | 2.315 | 0.721 | -1.594 | -3.502 | 0.029 | 0.261 | 2.238 | 5.678 | 0.006 | 0.110 | 0.644 | 1.634 | 0.185 | 0.954 | 0 | 0 | 0 | flat_flat |
| hsa-miR-585 | -1.625 | -1.183 | -0.761 | -0.924 | -0.163 | -0.351 | 0.745 | 1.000 | 0.643 | 1.600 | 0.192 | 0.959 | 0.481 | 1.195 | 0.304 | 1.000 | 0 | 0 | 0 | flat_flat |
| hsa-miR-586 | -0.536 | -1.434 | -0.208 | 0.036 | 0.244 | 0.393 | 0.716 | 1.000 | 0.777 | 1.448 | 0.228 | 1.000 | 1.021 | 1.902 | 0.137 | 0.754 | 0 | 0 | 0 | flat_flat |
| hsa-miR-587 | -1.862 | -1.623 | -2.444 | -2.414 | 0.030 | 0.072 | 0.946 | 1.000 | -0.702 | -1.923 | 0.134 | 0.742 | -0.671 | -1.839 | 0.147 | 0.790 | 0 | 0 | 0 | flat_flat |
| hsa-miR-588 | -0.958 | -0.306 | -1.939 | -2.216 | -0.277 | -0.524 | 0.630 | 1.000 | -1.307 | -2.858 | 0.052 | 0.384 | -1.584 | -3.463 | 0.030 | 0.256 | 0 | 0 | 0 | flat_flat |
| hsa-miR-589 | -0.780 | -0.630 | -2.398 | -1.274 | 1.124 | 2.743 | 0.058 | 0.420 | -1.693 | -4.770 | 0.011 | 0.151 | -0.569 | -1.603 | 0.191 | 0.974 | 0 | 0 | 0 | flat_flat |
| hsa-miR-589* | -1.005 | -2.599 | -1.108 | -1.643 | -0.535 | -0.576 | 0.598 | 1.000 | 0.694 | 0.863 | 0.441 | 1.000 | 0.159 | 0.198 | 0.854 | 1.000 | 0 | 0 | 0 | flat_flat |
| hsa-miR-590-3p | -1.399 | -1.886 | -1.040 | -0.288 | 0.753 | 1.579 | 0.197 | 1.000 | 0.602 | 1.459 | 0.225 | 1.000 | 1.355 | 3.283 | 0.035 | 0.282 | 0 | 0 | 0 | flat_flat |
| hsa-miR-590-5p | 10.942 | 9.556 | 6.080 | 5.975 | -0.105 | -0.127 | 0.906 | 1.000 | -4.169 | -5.790 | 0.006 | 0.106 | -4.275 | -5.936 | 0.005 | 0.075 | 0 | 0 | 0 | flat_flat |
| hsa-miR-591 | -0.764 | -1.862 | -0.588 | -0.740 | -0.153 | -0.217 | 0.840 | 1.000 | 0.726 | 1.192 | 0.305 | 1.000 | 0.573 | 0.941 | 0.405 | 1.000 | 0 | 0 | 0 | flat_flat |
| hsa-miR-592 | 0.678 | 0.577 | 0.553 | -0.772 | -1.325 | -3.266 | 0.036 | 0.299 | -0.075 | -0.212 | 0.843 | 1.000 | -1.400 | -3.983 | 0.020 | 0.189 | 0 | 0 | 0 | flat_flat |
| hsa-miR-593 | -0.531 | -0.997 | -0.539 | -0.146 | 0.393 | 0.836 | 0.455 | 1.000 | 0.225 | 0.551 | 0.614 | 1.000 | 0.618 | 1.516 | 0.211 | 1.000 | 0 | 0 | 0 | flat_flat |
| hsa-miR-593* | -2.056 | -2.113 | -1.733 | -1.348 | 0.385 | 0.954 | 0.399 | 1.000 | 0.351 | 1.006 | 0.377 | 1.000 | 0.736 | 2.107 | 0.110 | 0.653 | 0 | 0 | 0 | flat_flat |
| hsa-miR-595 | 6.911 | 9.797 | 1.600 | 4.609 | 3.010 | 1.920 | 0.135 | 0.776 | -6.755 | -4.974 | 0.010 | 0.140 | -3.745 | -2.758 | 0.057 | 0.397 | 0 | 0 | 0 | flat_flat |
| hsa-miR-596 | -1.049 | -0.460 | -0.356 | -2.309 | -1.953 | -3.848 | 0.022 | 0.210 | 0.398 | 0.907 | 0.421 | 1.000 | -1.554 | -3.536 | 0.028 | 0.247 | 0 | 0 | 0 | flat_flat |
| hsa-miR-597 | -1.722 | -1.550 | -1.575 | -1.160 | 0.415 | 1.006 | 0.377 | 1.000 | 0.061 | 0.172 | 0.873 | 1.000 | 0.476 | 1.334 | 0.260 | 1.000 | 0 | 0 | 0 | flat_flat |
| hsa-miR-599 | -1.275 | -1.375 | -0.512 | -1.275 | -0.763 | -1.881 | 0.140 | 0.800 | 0.813 | 2.314 | 0.088 | 0.547 | 0.050 | 0.141 | 0.895 | 1.000 | 0 | 0 | 0 | flat_flat |
| hsa-miR-600 | -0.901 | -0.814 | -2.041 | -1.897 | 0.145 | 0.357 | 0.741 | 1.000 | -1.184 | -3.377 | 0.032 | 0.284 | -1.039 | -2.964 | 0.047 | 0.350 | 0 | 0 | 0 | flat_flat |
| hsa-miR-602 | 3.576 | 3.907 | 2.818 | 5.396 | 2.578 | 5.882 | 0.006 | 0.083 | -0.924 | -2.433 | 0.078 | 0.510 | 1.654 | 4.359 | 0.015 | 0.158 | 0 | 0 | 0 | flat_flat |
| hsa-miR-603 | -1.411 | -0.787 | -0.536 | -0.020 | 0.516 | 0.994 | 0.382 | 1.000 | 0.563 | 1.253 | 0.285 | 1.000 | 1.079 | 2.401 | 0.081 | 0.516 | 0 | 0 | 0 | flat_flat |
| hsa-miR-604 | -1.825 | -1.479 | -0.591 | -1.258 | -0.667 | -1.511 | 0.213 | 1.000 | 1.061 | 2.775 | 0.056 | 0.402 | 0.394 | 1.031 | 0.366 | 1.000 | 0 | 0 | 0 | flat_flat |
| hsa-miR-605 | 1.271 | 1.327 | 0.322 | 0.291 | -0.031 | -0.076 | 0.943 | 1.000 | -0.978 | -2.799 | 0.055 | 0.399 | -1.008 | -2.887 | 0.050 | 0.369 | 0 | 0 | 0 | flat_flat |
| hsa-miR-606 | -1.991 | -1.487 | -1.612 | -1.226 | 0.386 | 0.802 | 0.472 | 1.000 | 0.128 | 0.306 | 0.776 | 1.000 | 0.514 | 1.232 | 0.292 | 1.000 | 0 | 0 | 0 | flat_flat |
| hsa-miR-607 | -1.475 | -0.412 | -1.825 | -0.913 | 0.912 | 1.326 | 0.262 | 1.000 | -0.881 | -1.479 | 0.220 | 1.000 | 0.031 | 0.052 | 0.961 | 1.000 | 0 | 0 | 0 | flat_flat |
| hsa-miR-608 | -1.392 | -1.587 | -0.543 | -1.439 | -0.896 | -2.159 | 0.104 | 0.636 | 0.946 | 2.632 | 0.064 | 0.444 | 0.050 | 0.139 | 0.897 | 1.000 | 0 | 0 | 0 | flat_flat |
| hsa-miR-609 | 2.670 | 3.296 | 1.399 | 1.435 | 0.036 | 0.070 | 0.948 | 1.000 | -1.584 | -3.520 | 0.029 | 0.265 | -1.548 | -3.440 | 0.031 | 0.260 | 0 | 0 | 0 | flat_flat |
| hsa-miR-610 | 0.169 | 0.912 | 0.036 | -0.060 | -0.096 | -0.171 | 0.873 | 1.000 | -0.505 | -1.039 | 0.363 | 1.000 | -0.600 | -1.237 | 0.290 | 1.000 | 0 | 0 | 0 | flat_flat |
| hsa-miR-611 | -1.191 | -0.963 | -1.168 | -0.919 | 0.249 | 0.594 | 0.588 | 1.000 | -0.091 | -0.250 | 0.816 | 1.000 | 0.158 | 0.436 | 0.688 | 1.000 | 0 | 0 | 0 | flat_flat |
| hsa-miR-612 | -0.394 | -0.950 | -0.455 | -0.053 | 0.402 | 0.809 | 0.468 | 1.000 | 0.217 | 0.503 | 0.644 | 1.000 | 0.619 | 1.437 | 0.231 | 1.000 | 0 | 0 | 0 | flat_flat |
| hsa-miR-613 | 1.744 | 1.264 | -0.323 | 0.886 | 1.209 | 2.547 | 0.070 | 0.472 | -1.827 | -4.444 | 0.014 | 0.172 | -0.618 | -1.503 | 0.214 | 1.000 | 0 | 0 | 0 | flat_flat |
| hsa-miR-614 | -0.561 | -0.794 | -0.446 | -0.950 | -0.504 | -1.200 | 0.303 | 1.000 | 0.232 | 0.636 | 0.562 | 1.000 | -0.273 | -0.749 | 0.500 | 1.000 | 0 | 0 | 0 | flat_flat |
| hsa-miR-615-3p | 0.980 | 0.737 | 1.125 | 0.847 | -0.278 | -0.660 | 0.549 | 1.000 | 0.266 | 0.729 | 0.510 | 1.000 | -0.012 | -0.033 | 0.975 | 1.000 | 0 | 0 | 0 | flat_flat |
| hsa-miR-615-5p | -0.924 | -1.840 | -2.659 | -1.814 | 0.845 | 1.348 | 0.256 | 1.000 | -1.277 | -2.352 | 0.085 | 0.534 | -0.432 | -0.796 | 0.475 | 1.000 | 0 | 0 | 0 | flat_flat |
| hsa-miR-616 | -1.683 | -1.932 | -0.623 | -2.190 | -1.567 | -3.706 | 0.025 | 0.228 | 1.184 | 3.234 | 0.037 | 0.300 | -0.383 | -1.045 | 0.361 | 1.000 | 0 | 0 | 0 | flat_flat |
| hsa-miR-616* | -0.504 | -1.370 | -1.202 | -0.477 | 0.725 | 1.195 | 0.304 | 1.000 | -0.265 | -0.503 | 0.644 | 1.000 | 0.461 | 0.876 | 0.435 | 1.000 | 0 | 0 | 0 | flat_flat |
| hsa-miR-617 | -0.275 | 0.169 | -0.690 | -0.343 | 0.347 | 0.747 | 0.500 | 1.000 | -0.637 | -1.582 | 0.196 | 0.971 | -0.290 | -0.719 | 0.515 | 1.000 | 0 | 0 | 0 | flat_flat |
| hsa-miR-618 | 0.196 | -0.173 | -1.628 | 0.269 | 1.897 | 4.249 | 0.016 | 0.170 | -1.639 | -4.240 | 0.016 | 0.188 | 0.257 | 0.666 | 0.545 | 1.000 | 0 | 0 | 0 | flat_flat |
| hsa-miR-619 | -2.014 | -2.309 | -0.725 | -0.572 | 0.154 | 0.357 | 0.741 | 1.000 | 1.436 | 3.847 | 0.022 | 0.227 | 1.590 | 4.259 | 0.016 | 0.162 | 0 | 0 | 0 | flat_flat |
| hsa-miR-620 | -1.245 | -0.882 | -1.031 | -1.139 | -0.108 | -0.242 | 0.822 | 1.000 | 0.033 | 0.084 | 0.937 | 1.000 | -0.075 | -0.195 | 0.856 | 1.000 | 0 | 0 | 0 | flat_flat |
| hsa-miR-621 | -0.769 | -1.609 | -1.148 | -1.508 | -0.359 | -0.601 | 0.583 | 1.000 | 0.041 | 0.079 | 0.941 | 1.000 | -0.318 | -0.615 | 0.575 | 1.000 | 0 | 0 | 0 | flat_flat |
| hsa-miR-622 | 1.564 | 2.670 | 2.012 | 0.209 | -1.803 | -2.553 | 0.069 | 0.471 | -0.105 | -0.171 | 0.873 | 1.000 | -1.908 | -3.120 | 0.041 | 0.317 | 0 | 0 | 0 | flat_flat |
| hsa-miR-623 | 2.693 | 4.422 | 3.273 | 0.940 | -2.334 | -2.350 | 0.085 | 0.547 | -0.284 | -0.330 | 0.759 | 1.000 | -2.618 | -3.043 | 0.044 | 0.334 | 0 | 0 | 0 | flat_flat |
| hsa-miR-624 | -1.504 | -0.764 | -0.345 | -0.173 | 0.172 | 0.308 | 0.775 | 1.000 | 0.789 | 1.629 | 0.186 | 0.936 | 0.961 | 1.985 | 0.125 | 0.705 | 0 | 0 | 0 | flat_flat |
| hsa-miR-624* | 0.447 | 0.418 | -0.127 | 0.960 | 1.088 | 2.702 | 0.060 | 0.427 | -0.560 | -1.607 | 0.191 | 0.956 | 0.527 | 1.513 | 0.212 | 1.000 | 0 | 0 | 0 | flat_flat |
| hsa-miR-625* | 1.646 | 1.125 | 1.857 | 4.074 | 2.217 | 4.556 | 0.013 | 0.149 | 0.472 | 1.120 | 0.332 | 1.000 | 2.688 | 6.380 | 0.004 | 0.061 | 0 | 0 | 0 | flat_flat |
| hsa-miR-626 | -2.017 | -1.963 | -1.162 | -0.623 | 0.538 | 1.335 | 0.259 | 1.000 | 0.828 | 2.372 | 0.083 | 0.528 | 1.367 | 3.914 | 0.021 | 0.196 | 0 | 0 | 0 | flat_flat |
| hsa-miR-627 | -1.311 | -0.451 | -1.191 | -0.126 | 1.065 | 1.761 | 0.160 | 0.880 | -0.310 | -0.592 | 0.589 | 1.000 | 0.755 | 1.442 | 0.230 | 1.000 | 0 | 0 | 0 | flat_flat |
| hsa-miR-628-3p | -2.638 | -1.125 | -0.218 | -1.752 | -1.533 | -1.722 | 0.167 | 0.908 | 1.663 | 2.157 | 0.104 | 0.620 | 0.130 | 0.168 | 0.875 | 1.000 | 0 | 0 | 0 | flat_flat |
| hsa-miR-628-5p | -0.143 | -0.798 | 0.544 | 0.071 | -0.473 | -0.893 | 0.427 | 1.000 | 1.014 | 2.212 | 0.098 | 0.594 | 0.541 | 1.181 | 0.309 | 1.000 | 0 | 0 | 0 | flat_flat |
| hsa-miR-629 | -0.836 | -0.448 | 1.161 | -0.180 | -1.341 | -2.974 | 0.046 | 0.360 | 1.803 | 4.617 | 0.012 | 0.159 | 0.462 | 1.183 | 0.308 | 1.000 | 0 | 0 | 0 | flat_flat |
| hsa-miR-631 | 0.692 | 1.367 | -0.684 | -0.302 | 0.382 | 0.713 | 0.519 | 1.000 | -1.714 | -3.691 | 0.025 | 0.242 | -1.331 | -2.867 | 0.051 | 0.370 | 0 | 0 | 0 | flat_flat |
| hsa-miR-632 | -0.580 | -0.428 | 0.443 | 0.067 | -0.376 | -0.916 | 0.416 | 1.000 | 0.946 | 2.665 | 0.062 | 0.435 | 0.571 | 1.607 | 0.191 | 0.974 | 0 | 0 | 0 | flat_flat |
| hsa-miR-633 | -0.719 | -1.223 | -2.868 | -0.409 | 2.459 | 5.106 | 0.009 | 0.115 | -1.897 | -4.548 | 0.013 | 0.164 | 0.562 | 1.349 | 0.255 | 1.000 | 0 | 0 | 0 | flat_flat |
| hsa-miR-635 | -1.462 | -0.695 | -1.357 | -1.191 | 0.166 | 0.292 | 0.787 | 1.000 | -0.279 | -0.566 | 0.605 | 1.000 | -0.113 | -0.229 | 0.831 | 1.000 | 0 | 0 | 0 | flat_flat |
| hsa-miR-636 | 3.296 | 3.113 | 2.208 | 3.340 | 1.132 | 2.736 | 0.058 | 0.420 | -0.997 | -2.782 | 0.056 | 0.401 | 0.135 | 0.377 | 0.727 | 1.000 | 0 | 0 | 0 | flat_flat |
| hsa-miR-637 | -0.443 | -1.114 | -0.475 | -0.251 | 0.224 | 0.418 | 0.699 | 1.000 | 0.303 | 0.654 | 0.552 | 1.000 | 0.527 | 1.137 | 0.325 | 1.000 | 0 | 0 | 0 | flat_flat |
| hsa-miR-638 | 13.143 | 13.618 | 13.432 | 12.902 | -0.530 | -1.120 | 0.331 | 1.000 | 0.052 | 0.126 | 0.906 | 1.000 | -0.479 | -1.168 | 0.314 | 1.000 | 0 | 0 | 0 | flat_flat |
| hsa-miR-639 | -2.170 | -2.031 | -1.197 | -1.921 | -0.724 | -1.771 | 0.159 | 0.878 | 0.903 | 2.551 | 0.070 | 0.473 | 0.179 | 0.506 | 0.642 | 1.000 | 0 | 0 | 0 | flat_flat |
| hsa-miR-640 | 0.642 | 1.110 | -0.896 | 0.716 | 1.613 | 3.420 | 0.031 | 0.268 | -1.772 | -4.340 | 0.015 | 0.179 | -0.160 | -0.391 | 0.718 | 1.000 | 0 | 0 | 0 | flat_flat |
| hsa-miR-641 | 0.354 | -0.060 | 0.705 | -1.168 | -1.873 | -4.096 | 0.018 | 0.183 | 0.558 | 1.409 | 0.239 | 1.000 | -1.315 | -3.321 | 0.034 | 0.280 | 0 | 0 | 0 | flat_flat |
| hsa-miR-642 | -0.691 | -0.829 | -1.364 | -0.896 | 0.467 | 1.144 | 0.323 | 1.000 | -0.603 | -1.705 | 0.171 | 0.881 | -0.136 | -0.384 | 0.722 | 1.000 | 0 | 0 | 0 | flat_flat |
| hsa-miR-643 | -1.921 | -2.322 | -1.921 | -1.245 | 0.676 | 1.490 | 0.218 | 1.000 | 0.200 | 0.509 | 0.640 | 1.000 | 0.876 | 2.230 | 0.097 | 0.590 | 0 | 0 | 0 | flat_flat |
| hsa-miR-644 | -0.905 | -1.426 | -0.570 | -0.729 | -0.160 | -0.328 | 0.761 | 1.000 | 0.596 | 1.415 | 0.237 | 1.000 | 0.436 | 1.036 | 0.364 | 1.000 | 0 | 0 | 0 | flat_flat |
| hsa-miR-645 | -0.953 | -0.221 | 0.148 | -1.368 | -1.516 | -2.722 | 0.059 | 0.422 | 0.735 | 1.524 | 0.209 | 1.000 | -0.781 | -1.619 | 0.188 | 0.966 | 0 | 0 | 0 | flat_flat |
| hsa-miR-646 | -0.306 | -0.863 | -1.298 | -1.635 | -0.337 | -0.677 | 0.539 | 1.000 | -0.714 | -1.657 | 0.180 | 0.911 | -1.051 | -2.439 | 0.078 | 0.506 | 0 | 0 | 0 | flat_flat |
| hsa-miR-647 | 1.341 | 1.139 | -0.977 | 0.322 | 1.299 | 3.122 | 0.041 | 0.326 | -2.217 | -6.155 | 0.005 | 0.098 | -0.918 | -2.550 | 0.070 | 0.468 | 0 | 0 | 0 | flat_flat |
| hsa-miR-648 | -1.912 | -0.965 | -0.765 | -1.700 | -0.935 | -1.460 | 0.225 | 1.000 | 0.673 | 1.215 | 0.298 | 1.000 | -0.261 | -0.471 | 0.664 | 1.000 | 0 | 0 | 0 | flat_flat |
| hsa-miR-649 | -1.932 | -1.117 | -1.013 | -0.997 | 0.016 | 0.028 | 0.979 | 1.000 | 0.511 | 1.005 | 0.377 | 1.000 | 0.527 | 1.037 | 0.364 | 1.000 | 0 | 0 | 0 | flat_flat |
| hsa-miR-651 | -1.763 | -1.162 | -0.383 | -0.854 | -0.470 | -0.919 | 0.415 | 1.000 | 1.079 | 2.436 | 0.078 | 0.510 | 0.609 | 1.375 | 0.248 | 1.000 | 0 | 0 | 0 | flat_flat |
| hsa-miR-653 | -1.795 | -0.710 | -2.322 | -1.254 | 1.068 | 1.531 | 0.208 | 1.000 | -1.069 | -1.770 | 0.159 | 0.827 | -0.001 | -0.002 | 0.999 | 1.000 | 0 | 0 | 0 | flat_flat |
| hsa-miR-654-3p | 0.838 | 0.917 | 0.286 | 2.706 | 2.419 | 5.982 | 0.005 | 0.079 | -0.591 | -1.688 | 0.174 | 0.889 | 1.828 | 5.220 | 0.008 | 0.102 | 0 | 0 | 0 | flat_flat |
| hsa-miR-654-5p | -0.337 | -0.297 | -0.126 | -1.572 | -1.446 | -3.590 | 0.027 | 0.244 | 0.191 | 0.549 | 0.615 | 1.000 | -1.255 | -3.597 | 0.027 | 0.238 | 0 | 0 | 0 | flat_flat |
| hsa-miR-655 | -2.005 | -1.907 | -0.147 | -0.443 | -0.296 | -0.729 | 0.510 | 1.000 | 1.809 | 5.151 | 0.009 | 0.131 | 1.513 | 4.310 | 0.016 | 0.159 | 0 | 0 | 0 | flat_flat |
| hsa-miR-656 | -0.250 | 0.042 | -0.597 | 0.121 | 0.718 | 1.667 | 0.178 | 0.949 | -0.493 | -1.322 | 0.263 | 1.000 | 0.225 | 0.603 | 0.582 | 1.000 | 0 | 0 | 0 | flat_flat |
| hsa-miR-657 | -0.208 | 0.275 | -2.264 | 0.030 | 2.294 | 4.826 | 0.011 | 0.133 | -2.297 | -5.581 | 0.007 | 0.112 | -0.003 | -0.008 | 0.994 | 1.000 | 0 | 0 | 0 | flat_flat |
| hsa-miR-658 | -1.514 | -1.254 | 0.258 | -1.484 | -1.743 | -4.101 | 0.018 | 0.183 | 1.642 | 4.463 | 0.014 | 0.171 | -0.100 | -0.272 | 0.800 | 1.000 | 0 | 0 | 0 | flat_flat |
| hsa-miR-659 | -0.512 | -0.510 | 0.645 | -1.049 | -1.694 | -4.211 | 0.017 | 0.172 | 1.156 | 3.320 | 0.034 | 0.291 | -0.538 | -1.543 | 0.205 | 1.000 | 0 | 0 | 0 | flat_flat |
| hsa-miR-661 | -0.623 | -0.931 | -0.823 | -0.972 | -0.148 | -0.342 | 0.751 | 1.000 | -0.046 | -0.123 | 0.909 | 1.000 | -0.195 | -0.518 | 0.634 | 1.000 | 0 | 0 | 0 | flat_flat |
| hsa-miR-662 | 3.907 | 6.437 | 1.769 | -1.311 | -3.080 | -2.219 | 0.098 | 0.605 | -3.403 | -2.832 | 0.053 | 0.391 | -6.483 | -5.395 | 0.008 | 0.094 | 0 | 0 | 0 | flat_flat |
| hsa-miR-663b | -0.637 | -0.337 | -1.991 | -1.851 | 0.140 | 0.325 | 0.763 | 1.000 | -1.504 | -4.021 | 0.019 | 0.206 | -1.364 | -3.646 | 0.026 | 0.233 | 0 | 0 | 0 | flat_flat |
| hsa-miR-664* | 0.823 | -0.394 | 1.489 | 2.583 | 1.094 | 1.449 | 0.228 | 1.000 | 1.274 | 1.949 | 0.130 | 0.732 | 2.368 | 3.623 | 0.026 | 0.235 | 0 | 0 | 0 | flat_flat |
| hsa-miR-668 | 0.097 | -0.147 | -0.854 | -0.577 | 0.277 | 0.656 | 0.551 | 1.000 | -0.829 | -2.267 | 0.093 | 0.568 | -0.552 | -1.509 | 0.213 | 1.000 | 0 | 0 | 0 | flat_flat |
| hsa-miR-671-3p | 0.258 | 0.071 | -1.323 | 0.443 | 1.766 | 4.265 | 0.016 | 0.170 | -1.488 | -4.150 | 0.017 | 0.196 | 0.278 | 0.775 | 0.486 | 1.000 | 0 | 0 | 0 | flat_flat |
| hsa-miR-675 | -2.698 | -1.529 | -2.309 | -2.056 | 0.253 | 0.344 | 0.750 | 1.000 | -0.195 | -0.307 | 0.776 | 1.000 | 0.057 | 0.090 | 0.933 | 1.000 | 0 | 0 | 0 | flat_flat |
| hsa-miR-675* | 0.544 | 0.470 | -0.804 | 0.428 | 1.232 | 3.048 | 0.043 | 0.345 | -1.311 | -3.746 | 0.024 | 0.238 | -0.079 | -0.226 | 0.833 | 1.000 | 0 | 0 | 0 | flat_flat |
| hsa-miR-7-2* | 0.084 | -0.512 | 0.807 | -0.371 | -1.178 | -2.311 | 0.089 | 0.559 | 1.021 | 2.314 | 0.088 | 0.547 | -0.156 | -0.354 | 0.743 | 1.000 | 0 | 0 | 0 | flat_flat |
| hsa-miR-708 | -0.680 | -0.977 | -0.012 | -1.886 | -1.874 | -4.343 | 0.015 | 0.163 | 0.816 | 2.185 | 0.101 | 0.607 | -1.057 | -2.830 | 0.053 | 0.379 | 0 | 0 | 0 | flat_flat |
| hsa-miR-708* | -1.293 | -2.444 | -1.064 | -1.504 | -0.441 | -0.607 | 0.580 | 1.000 | 0.805 | 1.281 | 0.276 | 1.000 | 0.365 | 0.580 | 0.596 | 1.000 | 0 | 0 | 0 | flat_flat |
| hsa-miR-720 | 11.862 | 12.152 | 12.612 | 12.030 | -0.582 | -1.353 | 0.254 | 1.000 | 0.605 | 1.624 | 0.187 | 0.940 | 0.023 | 0.062 | 0.954 | 1.000 | 0 | 0 | 0 | flat_flat |
| hsa-miR-744 | 5.102 | 4.695 | 6.180 | 3.961 | -2.219 | -4.871 | 0.010 | 0.130 | 1.281 | 3.247 | 0.036 | 0.300 | -0.938 | -2.378 | 0.083 | 0.526 | 0 | 0 | 0 | flat_flat |
| hsa-miR-744* | 5.073 | 3.989 | 0.485 | 1.201 | 0.716 | 1.027 | 0.368 | 1.000 | -4.046 | -6.703 | 0.004 | 0.081 | -3.330 | -5.517 | 0.007 | 0.089 | 0 | 0 | 0 | flat_flat |
| hsa-miR-758 | 1.399 | 1.054 | -1.139 | 0.980 | 2.118 | 4.802 | 0.011 | 0.134 | -2.365 | -6.191 | 0.005 | 0.098 | -0.247 | -0.646 | 0.557 | 1.000 | 0 | 0 | 0 | flat_flat |
| hsa-miR-760 | 0.999 | 4.281 | 1.545 | 3.674 | 2.129 | 1.203 | 0.302 | 1.000 | -1.095 | -0.715 | 0.518 | 1.000 | 1.034 | 0.675 | 0.541 | 1.000 | 0 | 0 | 0 | flat_flat |
| hsa-miR-765 | 1.043 | 2.285 | 2.533 | -0.127 | -2.661 | -3.473 | 0.030 | 0.262 | 0.869 | 1.310 | 0.267 | 1.000 | -1.791 | -2.700 | 0.060 | 0.411 | 0 | 0 | 0 | flat_flat |
| hsa-miR-767-5p | -1.840 | -0.901 | -0.615 | -1.336 | -0.722 | -1.134 | 0.326 | 1.000 | 0.756 | 1.371 | 0.249 | 1.000 | 0.034 | 0.061 | 0.954 | 1.000 | 0 | 0 | 0 | flat_flat |
| hsa-miR-769-3p | 0.033 | -0.320 | 0.501 | 0.354 | -0.146 | -0.330 | 0.760 | 1.000 | 0.644 | 1.679 | 0.176 | 0.894 | 0.498 | 1.298 | 0.271 | 1.000 | 0 | 0 | 0 | flat_flat |
| hsa-miR-769-5p | 4.422 | 3.890 | 6.464 | 6.600 | 0.136 | 0.277 | 0.797 | 1.000 | 2.308 | 5.440 | 0.007 | 0.117 | 2.444 | 5.759 | 0.006 | 0.081 | 0 | 0 | 0 | flat_flat |
| hsa-miR-802 | -1.747 | -1.484 | -0.750 | -1.000 | -0.250 | -0.587 | 0.592 | 1.000 | 0.865 | 2.349 | 0.085 | 0.534 | 0.616 | 1.671 | 0.177 | 0.928 | 0 | 0 | 0 | flat_flat |
| hsa-miR-873 | 1.335 | 0.222 | 0.789 | -0.580 | -1.368 | -1.929 | 0.133 | 0.770 | 0.010 | 0.016 | 0.988 | 1.000 | -1.358 | -2.211 | 0.098 | 0.600 | 0 | 0 | 0 | flat_flat |
| hsa-miR-874 | 10.889 | 10.942 | 11.719 | 9.128 | -2.591 | -6.426 | 0.004 | 0.066 | 0.804 | 2.301 | 0.090 | 0.550 | -1.788 | -5.119 | 0.009 | 0.107 | 0 | 0 | 0 | flat_flat |
| hsa-miR-875-3p | -2.483 | -2.330 | -1.975 | -1.612 | 0.363 | 0.886 | 0.431 | 1.000 | 0.432 | 1.215 | 0.297 | 1.000 | 0.795 | 2.238 | 0.096 | 0.588 | 0 | 0 | 0 | flat_flat |
| hsa-miR-875-5p | 0.004 | -0.563 | -0.250 | -0.260 | -0.010 | -0.019 | 0.986 | 1.000 | 0.029 | 0.067 | 0.950 | 1.000 | 0.020 | 0.045 | 0.966 | 1.000 | 0 | 0 | 0 | flat_flat |
| hsa-miR-876-3p | -0.556 | -1.360 | 1.254 | -0.858 | -2.112 | -3.621 | 0.026 | 0.240 | 2.212 | 4.378 | 0.015 | 0.177 | 0.100 | 0.197 | 0.854 | 1.000 | 0 | 0 | 0 | flat_flat |
| hsa-miR-876-5p | -0.412 | -0.389 | 1.335 | -0.066 | -1.401 | -3.481 | 0.030 | 0.262 | 1.736 | 4.981 | 0.010 | 0.140 | 0.335 | 0.961 | 0.396 | 1.000 | 0 | 0 | 0 | flat_flat |
| hsa-miR-877* | 5.837 | 7.399 | 3.153 | 7.554 | 4.401 | 4.818 | 0.011 | 0.133 | -3.465 | -4.380 | 0.015 | 0.177 | 0.936 | 1.184 | 0.308 | 1.000 | 0 | 0 | 0 | flat_flat |
| hsa-miR-885-3p | 2.488 | 4.473 | -0.691 | -1.445 | -0.754 | -0.675 | 0.540 | 1.000 | -4.172 | -4.310 | 0.015 | 0.181 | -4.926 | -5.089 | 0.009 | 0.108 | 0 | 0 | 0 | flat_flat |
| hsa-miR-885-5p | 3.890 | 3.843 | 1.534 | 3.016 | 1.482 | 3.678 | 0.025 | 0.232 | -2.332 | -6.682 | 0.004 | 0.082 | -0.850 | -2.435 | 0.078 | 0.506 | 0 | 0 | 0 | flat_flat |
| hsa-miR-886-3p | 10.798 | 9.457 | 13.200 | 13.410 | 0.210 | 0.259 | 0.810 | 1.000 | 3.073 | 4.375 | 0.015 | 0.177 | 3.282 | 4.674 | 0.012 | 0.135 | 0 | 0 | 0 | flat_flat |
| hsa-miR-886-5p | -1.375 | -2.820 | -0.041 | -1.527 | -1.486 | -1.730 | 0.166 | 0.901 | 2.057 | 2.765 | 0.056 | 0.405 | 0.570 | 0.767 | 0.490 | 1.000 | 0 | 0 | 0 | flat_flat |
| hsa-miR-887 | 1.466 | 0.148 | 3.381 | 0.004 | -3.377 | -4.219 | 0.017 | 0.172 | 2.574 | 3.713 | 0.024 | 0.241 | -0.803 | -1.158 | 0.317 | 1.000 | 0 | 0 | 0 | flat_flat |
| hsa-miR-888 | -1.007 | -0.772 | -1.086 | -1.210 | -0.125 | -0.297 | 0.783 | 1.000 | -0.196 | -0.538 | 0.622 | 1.000 | -0.321 | -0.880 | 0.433 | 1.000 | 0 | 0 | 0 | flat_flat |
| hsa-miR-888* | -1.265 | -0.317 | -1.134 | -0.376 | 0.758 | 1.185 | 0.308 | 1.000 | -0.344 | -0.620 | 0.572 | 1.000 | 0.415 | 0.748 | 0.500 | 1.000 | 0 | 0 | 0 | flat_flat |
| hsa-miR-889 | -0.855 | -1.146 | -1.283 | -0.071 | 1.212 | 2.816 | 0.054 | 0.400 | -0.283 | -0.758 | 0.495 | 1.000 | 0.929 | 2.493 | 0.074 | 0.487 | 0 | 0 | 0 | flat_flat |
| hsa-miR-890 | -2.512 | -2.014 | -2.064 | -2.444 | -0.381 | -0.793 | 0.476 | 1.000 | 0.199 | 0.480 | 0.659 | 1.000 | -0.181 | -0.436 | 0.687 | 1.000 | 0 | 0 | 0 | flat_flat |
| hsa-miR-891a | -1.016 | -1.210 | -1.348 | -0.690 | 0.658 | 1.587 | 0.195 | 1.000 | -0.235 | -0.655 | 0.552 | 1.000 | 0.423 | 1.177 | 0.311 | 1.000 | 0 | 0 | 0 | flat_flat |
| hsa-miR-891b | -0.329 | -0.409 | -1.223 | -0.255 | 0.968 | 2.394 | 0.082 | 0.531 | -0.855 | -2.440 | 0.078 | 0.509 | 0.113 | 0.324 | 0.764 | 1.000 | 0 | 0 | 0 | flat_flat |
| hsa-miR-892a | 0.280 | -0.625 | 0.447 | 0.912 | 0.465 | 0.747 | 0.500 | 1.000 | 0.620 | 1.150 | 0.320 | 1.000 | 1.085 | 2.013 | 0.122 | 0.690 | 0 | 0 | 0 | flat_flat |
| hsa-miR-892b | 9.679 | 14.220 | 4.630 | 15.929 | 11.299 | 4.672 | 0.012 | 0.143 | -7.319 | -3.494 | 0.029 | 0.270 | 3.980 | 1.900 | 0.138 | 0.754 | 0 | 0 | 0 | flat_flat |
| hsa-miR-920 | -3.172 | -3.014 | -1.800 | -2.669 | -0.869 | -2.116 | 0.109 | 0.659 | 1.293 | 3.635 | 0.026 | 0.250 | 0.424 | 1.192 | 0.305 | 1.000 | 0 | 0 | 0 | flat_flat |
| hsa-miR-921 | -2.599 | -2.126 | -3.098 | -1.963 | 1.135 | 2.401 | 0.081 | 0.528 | -0.735 | -1.796 | 0.154 | 0.816 | 0.400 | 0.976 | 0.390 | 1.000 | 0 | 0 | 0 | flat_flat |
| hsa-miR-922 | -2.072 | -2.765 | -2.947 | -2.868 | 0.079 | 0.146 | 0.892 | 1.000 | -0.529 | -1.125 | 0.329 | 1.000 | -0.449 | -0.957 | 0.398 | 1.000 | 0 | 0 | 0 | flat_flat |
| hsa-miR-923 | 12.388 | 12.341 | 12.562 | 12.388 | -0.174 | -0.431 | 0.691 | 1.000 | 0.197 | 0.565 | 0.605 | 1.000 | 0.023 | 0.067 | 0.950 | 1.000 | 0 | 0 | 0 | flat_flat |
| hsa-miR-924 | -0.574 | 0.403 | 0.557 | 0.478 | -0.078 | -0.120 | 0.911 | 1.000 | 0.642 | 1.137 | 0.325 | 1.000 | 0.564 | 0.998 | 0.380 | 1.000 | 0 | 0 | 0 | flat_flat |
| hsa-miR-92a | 11.951 | 11.842 | 12.866 | 12.773 | -0.093 | -0.228 | 0.832 | 1.000 | 0.969 | 2.755 | 0.057 | 0.408 | 0.877 | 2.492 | 0.074 | 0.487 | 0 | 0 | 0 | flat_flat |
| hsa-miR-92b* | -2.463 | -2.947 | -2.698 | -2.765 | -0.067 | -0.141 | 0.896 | 1.000 | 0.007 | 0.017 | 0.987 | 1.000 | -0.060 | -0.145 | 0.892 | 1.000 | 0 | 0 | 0 | flat_flat |
| hsa-miR-93 | 9.962 | 10.431 | 11.580 | 11.069 | -0.511 | -1.084 | 0.345 | 1.000 | 1.384 | 3.388 | 0.032 | 0.284 | 0.873 | 2.136 | 0.107 | 0.642 | 0 | 0 | 0 | flat_flat |
| hsa-miR-93* | -0.309 | -1.265 | -0.695 | 1.727 | 2.422 | 3.767 | 0.023 | 0.219 | 0.092 | 0.166 | 0.877 | 1.000 | 2.514 | 4.515 | 0.013 | 0.145 | 0 | 0 | 0 | flat_flat |
| hsa-miR-934 | -2.947 | -2.698 | -1.659 | -2.505 | -0.846 | -1.999 | 0.123 | 0.728 | 1.163 | 3.176 | 0.039 | 0.307 | 0.318 | 0.868 | 0.439 | 1.000 | 0 | 0 | 0 | flat_flat |
| hsa-miR-935 | -0.120 | 0.153 | -1.405 | -1.293 | 0.112 | 0.262 | 0.807 | 1.000 | -1.422 | -3.846 | 0.022 | 0.227 | -1.310 | -3.543 | 0.028 | 0.247 | 0 | 0 | 0 | flat_flat |
| hsa-miR-936 | -1.108 | -0.251 | 0.251 | -1.599 | -1.850 | -3.067 | 0.043 | 0.342 | 0.931 | 1.781 | 0.157 | 0.821 | -0.920 | -1.760 | 0.161 | 0.849 | 0 | 0 | 0 | flat_flat |
| hsa-miR-937 | 2.706 | 2.894 | 0.042 | 2.965 | 2.924 | 7.058 | 0.003 | 0.055 | -2.758 | -7.688 | 0.002 | 0.061 | 0.166 | 0.462 | 0.670 | 1.000 | 0 | 0 | 0 | flat_flat |
| hsa-miR-938 | -2.505 | -1.659 | -2.586 | -1.948 | 0.638 | 1.065 | 0.353 | 1.000 | -0.504 | -0.972 | 0.391 | 1.000 | 0.134 | 0.258 | 0.810 | 1.000 | 0 | 0 | 0 | flat_flat |
| hsa-miR-939 | 12.739 | 13.025 | 10.889 | 13.316 | 2.427 | 5.653 | 0.006 | 0.091 | -1.993 | -5.360 | 0.008 | 0.120 | 0.434 | 1.168 | 0.314 | 1.000 | 0 | 0 | 0 | flat_flat |
| hsa-miR-940 | 11.842 | 11.862 | 12.090 | 12.562 | 0.472 | 1.173 | 0.312 | 1.000 | 0.238 | 0.683 | 0.536 | 1.000 | 0.710 | 2.037 | 0.119 | 0.680 | 0 | 0 | 0 | flat_flat |
| hsa-miR-941 | -2.586 | -2.659 | -1.423 | -3.628 | -2.205 | -5.457 | 0.007 | 0.097 | 1.200 | 3.428 | 0.031 | 0.279 | -1.006 | -2.874 | 0.051 | 0.370 | 0 | 0 | 0 | flat_flat |
| hsa-miR-942 | -0.015 | -0.431 | 0.729 | -0.166 | -0.896 | -1.957 | 0.129 | 0.753 | 0.952 | 2.402 | 0.081 | 0.517 | 0.056 | 0.142 | 0.894 | 1.000 | 0 | 0 | 0 | flat_flat |
| hsa-miR-943 | -0.455 | -1.191 | -1.215 | -1.352 | -0.138 | -0.247 | 0.818 | 1.000 | -0.391 | -0.810 | 0.468 | 1.000 | -0.529 | -1.096 | 0.341 | 1.000 | 0 | 0 | 0 | flat_flat |
| hsa-miR-944 | -1.587 | -1.643 | -0.232 | -1.342 | -1.109 | -2.751 | 0.057 | 0.420 | 1.382 | 3.958 | 0.020 | 0.215 | 0.273 | 0.782 | 0.482 | 1.000 | 0 | 0 | 0 | flat_flat |
| hsa-miR-95 | 0.286 | 0.350 | 0.634 | 0.416 | -0.219 | -0.542 | 0.620 | 1.000 | 0.317 | 0.906 | 0.421 | 1.000 | 0.098 | 0.280 | 0.795 | 1.000 | 0 | 0 | 0 | flat_flat |
| hsa-miR-96* | -2.113 | -1.722 | -2.126 | -1.676 | 0.449 | 0.995 | 0.381 | 1.000 | -0.208 | -0.532 | 0.626 | 1.000 | 0.241 | 0.617 | 0.574 | 1.000 | 0 | 0 | 0 | flat_flat |
| hsa-miR-99a* | -1.210 | -1.142 | -0.986 | -1.092 | -0.106 | -0.263 | 0.807 | 1.000 | 0.190 | 0.542 | 0.619 | 1.000 | 0.084 | 0.239 | 0.824 | 1.000 | 0 | 0 | 0 | flat_flat |
| hsa-miR-99b | 9.797 | 8.406 | 11.891 | 8.342 | -3.549 | -4.257 | 0.016 | 0.170 | 2.789 | 3.863 | 0.022 | 0.227 | -0.760 | -1.052 | 0.358 | 1.000 | 0 | 0 | 0 | flat_flat |
| hsa-miR-99b* | -0.317 | -0.182 | -1.121 | -0.297 | 0.824 | 2.017 | 0.121 | 0.716 | -0.872 | -2.465 | 0.076 | 0.501 | -0.048 | -0.136 | 0.899 | 1.000 | 0 | 0 | 0 | flat_flat |
| hsa-miR-153 | 0.705 | 0.960 | -1.368 | -2.082 | -0.714 | -1.685 | 0.175 | 0.938 | -2.200 | -5.993 | 0.005 | 0.100 | -2.915 | -7.940 | 0.002 | 0.038 | 0 | 0 | -1 | flat_flat |
| hsa-miR-193a-3p | 5.734 | 4.747 | 3.401 | 0.470 | -2.930 | -4.465 | 0.014 | 0.156 | -1.840 | -3.237 | 0.037 | 0.300 | -4.770 | -8.392 | 0.002 | 0.033 | 0 | 0 | -1 | flat_flat |
| hsa-miR-199a-3p | 13.472 | 13.306 | 13.354 | 10.620 | -2.734 | -6.645 | 0.004 | 0.062 | -0.035 | -0.098 | 0.927 | 1.000 | -2.769 | -7.771 | 0.002 | 0.039 | 0 | 0 | -1 | flat_flat |
| hsa-miR-199a-5p | 8.406 | 9.128 | 8.135 | 4.422 | -3.712 | -6.717 | 0.004 | 0.061 | -0.633 | -1.322 | 0.263 | 1.000 | -4.345 | -9.078 | 0.001 | 0.026 | 0 | 0 | -1 | flat_flat |
| hsa-miR-301b | 8.660 | 6.600 | 6.437 | 0.113 | -6.324 | -5.478 | 0.007 | 0.097 | -1.194 | -1.194 | 0.305 | 1.000 | -7.517 | -7.520 | 0.002 | 0.042 | 0 | 0 | -1 | flat_flat |
| hsa-miR-31 | 3.082 | 2.390 | 1.248 | -0.924 | -2.172 | -4.007 | 0.019 | 0.194 | -1.488 | -3.169 | 0.039 | 0.307 | -3.660 | -7.796 | 0.002 | 0.039 | 0 | 0 | -1 | flat_flat |
| hsa-miR-340 | 10.307 | 8.748 | 5.073 | 3.153 | -1.920 | -2.105 | 0.110 | 0.664 | -4.454 | -5.639 | 0.007 | 0.111 | -6.374 | -8.069 | 0.002 | 0.036 | 0 | 0 | -1 | flat_flat |
| hsa-miR-498 | 5.975 | 5.201 | 1.709 | 1.264 | -0.445 | -0.778 | 0.484 | 1.000 | -3.879 | -7.835 | 0.002 | 0.058 | -4.324 | -8.734 | 0.001 | 0.029 | 0 | 0 | -1 | flat_flat |
| hsa-miR-516a-5p | 0.940 | 1.077 | -0.699 | -1.659 | -0.960 | -2.350 | 0.085 | 0.547 | -1.707 | -4.824 | 0.011 | 0.147 | -2.667 | -7.537 | 0.002 | 0.042 | 0 | 0 | -1 | flat_flat |
| hsa-miR-518e* | 1.926 | 1.435 | 0.217 | -1.683 | -1.900 | -3.977 | 0.020 | 0.198 | -1.464 | -3.538 | 0.028 | 0.262 | -3.364 | -8.131 | 0.002 | 0.036 | 0 | 0 | -1 | flat_flat |
| hsa-miR-520f | 4.695 | 4.074 | 1.139 | 0.999 | -0.140 | -0.270 | 0.802 | 1.000 | -3.245 | -7.235 | 0.003 | 0.069 | -3.385 | -7.547 | 0.002 | 0.042 | 0 | 0 | -1 | flat_flat |
| hsa-miR-601 | 6.261 | 8.112 | 5.613 | 0.598 | -5.015 | -4.766 | 0.011 | 0.136 | -1.574 | -1.727 | 0.167 | 0.860 | -6.589 | -7.230 | 0.003 | 0.047 | 0 | 0 | -1 | flat_flat |
| hsa-miR-9* | 5.233 | 3.935 | 5.004 | -0.512 | -5.516 | -6.970 | 0.003 | 0.057 | 0.420 | 0.613 | 0.576 | 1.000 | -5.096 | -7.435 | 0.003 | 0.043 | 0 | 0 | -1 | flat_flat |
| hsa-miR-92a-2* | 2.157 | 2.139 | 2.111 | -0.676 | -2.787 | -6.928 | 0.003 | 0.057 | -0.037 | -0.106 | 0.921 | 1.000 | -2.824 | -8.106 | 0.002 | 0.036 | 0 | 0 | -1 | flat_flat |
| hsa-miR-518b | 4.281 | 3.702 | 0.196 | 0.950 | 0.754 | 1.496 | 0.216 | 1.000 | -3.796 | -8.693 | 0.001 | 0.045 | -3.042 | -6.966 | 0.003 | 0.051 | 0 | -1 | 0 | down_flat |
| hsa-miR-575 | 12.976 | 13.143 | 10.046 | 11.603 | 1.557 | 3.781 | 0.023 | 0.217 | -3.014 | -8.451 | 0.002 | 0.047 | -1.457 | -4.085 | 0.018 | 0.180 | 0 | -1 | 0 | down_flat |
| hsa-miR-106a* | 7.399 | 6.911 | 2.670 | 1.012 | -1.658 | -3.478 | 0.030 | 0.262 | -4.485 | -10.861 | 0.001 | 0.026 | -6.143 | -14.877 | 0.000 | 0.008 | 0 | -1 | -1 | down_flat |
| hsa-miR-124 | 11.419 | 11.069 | 1.110 | -2.072 | -3.182 | -7.196 | 0.003 | 0.052 | -10.134 | -26.459 | 0.000 | 0.007 | -13.316 | -34.768 | 0.000 | 0.002 | 0 | -1 | -1 | down_flat |
| hsa-miR-1323 | 9.502 | 8.516 | 0.972 | 2.068 | 1.096 | 1.673 | 0.177 | 0.945 | -8.037 | -14.161 | 0.000 | 0.019 | -6.941 | -12.229 | 0.000 | 0.012 | 0 | -1 | -1 | down_flat |
| hsa-miR-204 | 9.468 | 8.437 | 2.368 | 2.079 | -0.288 | -0.428 | 0.693 | 1.000 | -6.585 | -11.276 | 0.001 | 0.024 | -6.873 | -11.770 | 0.001 | 0.014 | 0 | -1 | -1 | down_flat |
| hsa-miR-219-5p | 7.925 | 7.328 | -1.069 | 1.271 | 2.340 | 4.589 | 0.013 | 0.148 | -8.695 | -19.690 | 0.000 | 0.010 | -6.355 | -14.392 | 0.000 | 0.008 | 0 | -1 | -1 | down_flat |
| hsa-miR-302b* | 12.825 | 12.395 | 2.641 | -0.489 | -3.130 | -6.783 | 0.003 | 0.059 | -9.969 | -24.945 | 0.000 | 0.007 | -13.099 | -32.777 | 0.000 | 0.002 | 0 | -1 | -1 | down_flat |
| hsa-miR-31* | 5.396 | 4.860 | -0.951 | -0.926 | 0.025 | 0.051 | 0.962 | 1.000 | -6.079 | -14.298 | 0.000 | 0.019 | -6.054 | -14.239 | 0.000 | 0.008 | 0 | -1 | -1 | down_flat |
| hsa-miR-512-3p | 12.562 | 12.090 | 0.585 | 1.229 | 0.643 | 1.362 | 0.252 | 1.000 | -11.741 | -28.696 | 0.000 | 0.007 | -11.097 | -27.124 | 0.000 | 0.003 | 0 | -1 | -1 | down_flat |
| hsa-miR-513a-5p | 8.437 | 8.252 | 3.989 | 5.734 | 1.745 | 4.217 | 0.017 | 0.172 | -4.355 | -12.153 | 0.000 | 0.024 | -2.610 | -7.283 | 0.003 | 0.046 | 0 | -1 | -1 | down_flat |
| hsa-miR-515-3p | 6.464 | 5.837 | 1.086 | 1.054 | -0.032 | -0.062 | 0.954 | 1.000 | -5.065 | -11.248 | 0.001 | 0.024 | -5.097 | -11.319 | 0.001 | 0.015 | 0 | -1 | -1 | down_flat |
| hsa-miR-515-5p | 9.403 | 7.814 | -0.838 | -0.631 | 0.207 | 0.224 | 0.835 | 1.000 | -9.447 | -11.776 | 0.001 | 0.024 | -9.239 | -11.518 | 0.001 | 0.014 | 0 | -1 | -1 | down_flat |
| hsa-miR-516b | 7.328 | 5.778 | -0.159 | -1.100 | -0.942 | -1.037 | 0.364 | 1.000 | -6.712 | -8.536 | 0.002 | 0.046 | -7.653 | -9.733 | 0.001 | 0.021 | 0 | -1 | -1 | down_flat |
| hsa-miR-517a | 9.556 | 9.403 | 1.043 | 0.086 | -0.957 | -2.332 | 0.087 | 0.554 | -8.437 | -23.748 | 0.000 | 0.007 | -9.394 | -26.441 | 0.000 | 0.003 | 0 | -1 | -1 | down_flat |
| hsa-miR-517b | 10.560 | 9.502 | 0.428 | 0.608 | 0.181 | 0.263 | 0.807 | 1.000 | -9.603 | -16.163 | 0.000 | 0.016 | -9.422 | -15.858 | 0.000 | 0.007 | 0 | -1 | -1 | down_flat |
| hsa-miR-519b-3p | 6.180 | 5.657 | 0.847 | 0.737 | -0.109 | -0.224 | 0.835 | 1.000 | -5.072 | -12.031 | 0.000 | 0.024 | -5.181 | -12.289 | 0.000 | 0.012 | 0 | -1 | -1 | down_flat |
| hsa-miR-519d | 4.630 | 5.051 | 0.912 | 1.073 | 0.160 | 0.349 | 0.746 | 1.000 | -3.928 | -9.882 | 0.001 | 0.033 | -3.768 | -9.479 | 0.001 | 0.023 | 0 | -1 | -1 | down_flat |
| hsa-miR-520a-5p | 4.609 | 4.566 | -0.821 | -1.039 | -0.218 | -0.541 | 0.620 | 1.000 | -5.409 | -15.502 | 0.000 | 0.017 | -5.627 | -16.127 | 0.000 | 0.007 | 0 | -1 | -1 | down_flat |
| hsa-miR-520c-3p | 12.439 | 12.030 | 0.688 | 1.800 | 1.112 | 2.439 | 0.078 | 0.515 | -11.546 | -29.244 | 0.000 | 0.007 | -10.434 | -26.427 | 0.000 | 0.003 | 0 | -1 | -1 | down_flat |
| hsa-miR-525-5p | 10.730 | 8.929 | 0.336 | 0.105 | -0.231 | -0.225 | 0.834 | 1.000 | -9.493 | -10.665 | 0.001 | 0.028 | -9.724 | -10.925 | 0.001 | 0.016 | 0 | -1 | -1 | down_flat |
| hsa-miR-526b | 3.702 | 3.340 | 0.169 | -0.478 | -0.647 | -1.455 | 0.226 | 1.000 | -3.352 | -8.698 | 0.001 | 0.045 | -3.999 | -10.378 | 0.001 | 0.018 | 0 | -1 | -1 | down_flat |
| hsa-miR-545 | 3.248 | 3.016 | -0.027 | -0.320 | -0.292 | -0.695 | 0.529 | 1.000 | -3.160 | -8.682 | 0.001 | 0.045 | -3.452 | -9.485 | 0.001 | 0.023 | 0 | -1 | -1 | down_flat |
| hsa-miR-629* | 17.496 | 16.676 | 2.196 | 1.478 | -0.718 | -1.219 | 0.296 | 1.000 | -14.890 | -29.184 | 0.000 | 0.007 | -15.608 | -30.591 | 0.000 | 0.002 | 0 | -1 | -1 | down_flat |
| hsa-miR-877 | 12.469 | 14.928 | 1.646 | -1.609 | -3.255 | -2.407 | 0.080 | 0.527 | -12.052 | -10.290 | 0.001 | 0.029 | -15.308 | -13.069 | 0.000 | 0.011 | 0 | -1 | -1 | down_flat |
| hsa-miR-33b* | 1.876 | 1.943 | 14.928 | 8.516 | -6.411 | -15.880 | 0.000 | 0.009 | 13.018 | 37.232 | 0.000 | 0.007 | 6.607 | 18.896 | 0.000 | 0.005 | -1 | 1 | 1 | up_down |
| hsa-miR-122 | 0.076 | 0.504 | 13.653 | -0.232 | -13.886 | -30.141 | 0.000 | 0.005 | 13.363 | 33.494 | 0.000 | 0.007 | -0.523 | -1.310 | 0.267 | 1.000 | -1 | 1 | 0 | up_down |
| hsa-miR-122* | -0.499 | -0.597 | 4.801 | 0.403 | -4.398 | -10.845 | 0.001 | 0.020 | 5.349 | 15.232 | 0.000 | 0.017 | 0.951 | 2.709 | 0.060 | 0.409 | -1 | 1 | 0 | up_down |
| hsa-miR-132 | -0.073 | 0.113 | 8.890 | 1.139 | -7.751 | -18.726 | 0.000 | 0.007 | 8.871 | 24.745 | 0.000 | 0.007 | 1.119 | 3.123 | 0.041 | 0.317 | -1 | 1 | 0 | up_down |
| hsa-miR-132* | -0.051 | -0.267 | 3.340 | 0.258 | -3.081 | -7.372 | 0.003 | 0.049 | 3.499 | 9.666 | 0.001 | 0.034 | 0.418 | 1.153 | 0.319 | 1.000 | -1 | 1 | 0 | up_down |
| hsa-miR-139-5p | -0.545 | -1.051 | 5.778 | -0.468 | -6.246 | -12.959 | 0.000 | 0.014 | 6.576 | 15.754 | 0.000 | 0.016 | 0.330 | 0.790 | 0.478 | 1.000 | -1 | 1 | 0 | up_down |
| hsa-miR-145 | 3.401 | 2.785 | 7.399 | 3.205 | -4.194 | -8.129 | 0.002 | 0.038 | 4.306 | 9.638 | 0.001 | 0.034 | 0.112 | 0.251 | 0.816 | 1.000 | -1 | 1 | 0 | up_down |
| hsa-miR-1471 | 0.139 | 0.126 | 5.396 | 1.248 | -4.148 | -10.311 | 0.001 | 0.022 | 5.263 | 15.108 | 0.000 | 0.017 | 1.115 | 3.201 | 0.038 | 0.300 | -1 | 1 | 0 | up_down |
| hsa-miR-192 | 2.785 | 2.488 | 8.198 | 3.576 | -4.622 | -10.709 | 0.001 | 0.020 | 5.561 | 14.880 | 0.000 | 0.017 | 0.939 | 2.514 | 0.072 | 0.480 | -1 | 1 | 0 | up_down |
| hsa-miR-193a-5p | -0.510 | -1.086 | 4.473 | -0.630 | -5.103 | -10.145 | 0.001 | 0.023 | 5.271 | 12.101 | 0.000 | 0.024 | 0.168 | 0.387 | 0.721 | 1.000 | -1 | 1 | 0 | up_down |
| hsa-miR-195 | 4.200 | 3.381 | 11.096 | 4.105 | -6.991 | -11.876 | 0.000 | 0.017 | 7.305 | 14.330 | 0.000 | 0.019 | 0.314 | 0.616 | 0.574 | 1.000 | -1 | 1 | 0 | up_down |
| hsa-miR-198 | -0.461 | -0.404 | 3.771 | -0.725 | -4.497 | -11.148 | 0.001 | 0.019 | 4.204 | 12.035 | 0.000 | 0.024 | -0.293 | -0.838 | 0.454 | 1.000 | -1 | 1 | 0 | up_down |
| hsa-miR-215 | 0.688 | 0.823 | 4.281 | 0.042 | -4.239 | -10.381 | 0.001 | 0.022 | 3.526 | 9.969 | 0.001 | 0.032 | -0.714 | -2.018 | 0.121 | 0.689 | -1 | 1 | 0 | up_down |
| hsa-miR-224 | 0.113 | 0.627 | 9.962 | -0.114 | -10.076 | -20.796 | 0.000 | 0.007 | 9.592 | 22.861 | 0.000 | 0.007 | -0.484 | -1.153 | 0.319 | 1.000 | -1 | 1 | 0 | up_down |
| hsa-miR-30a* | 2.079 | 1.237 | 8.406 | -1.077 | -9.483 | -15.868 | 0.000 | 0.009 | 6.748 | 13.038 | 0.000 | 0.022 | -2.735 | -5.285 | 0.008 | 0.099 | -1 | 1 | 0 | up_down |
| hsa-miR-362-5p | 2.139 | 1.534 | 10.307 | 1.672 | -8.634 | -16.849 | 0.000 | 0.008 | 8.470 | 19.085 | 0.000 | 0.010 | -0.164 | -0.370 | 0.732 | 1.000 | -1 | 1 | 0 | up_down |
| hsa-miR-371-3p | 1.125 | 1.271 | 11.824 | 0.591 | -11.233 | -27.429 | 0.000 | 0.005 | 10.626 | 29.962 | 0.000 | 0.007 | -0.607 | -1.711 | 0.170 | 0.891 | -1 | 1 | 0 | up_down |
| hsa-miR-373 | 2.243 | 1.709 | 10.700 | 0.447 | -10.253 | -20.909 | 0.000 | 0.007 | 8.724 | 20.544 | 0.000 | 0.009 | -1.529 | -3.599 | 0.027 | 0.238 | -1 | 1 | 0 | up_down |
| hsa-miR-375 | -1.105 | -1.700 | 8.660 | -0.963 | -9.624 | -18.901 | 0.000 | 0.007 | 10.063 | 22.821 | 0.000 | 0.007 | 0.439 | 0.996 | 0.381 | 1.000 | -1 | 1 | 0 | up_down |
| hsa-miR-454 | 3.487 | 3.229 | 7.554 | 3.733 | -3.822 | -9.003 | 0.001 | 0.031 | 4.196 | 11.415 | 0.001 | 0.024 | 0.374 | 1.019 | 0.371 | 1.000 | -1 | 1 | 0 | up_down |
| hsa-miR-483-3p | 5.438 | 6.180 | 9.998 | 5.837 | -4.161 | -7.434 | 0.003 | 0.048 | 4.189 | 8.642 | 0.002 | 0.045 | 0.028 | 0.058 | 0.957 | 1.000 | -1 | 1 | 0 | up_down |
| hsa-miR-489 | 0.917 | 0.762 | 10.620 | -0.769 | -11.389 | -27.751 | 0.000 | 0.005 | 9.780 | 27.518 | 0.000 | 0.007 | -1.609 | -4.526 | 0.013 | 0.145 | -1 | 1 | 0 | up_down |
| hsa-miR-497 | 0.134 | -0.204 | 6.501 | -0.280 | -6.782 | -15.421 | 0.000 | 0.009 | 6.536 | 17.164 | 0.000 | 0.014 | -0.245 | -0.644 | 0.558 | 1.000 | -1 | 1 | 0 | up_down |
| hsa-miR-500* | 0.634 | 0.011 | 6.300 | 1.408 | -4.893 | -9.435 | 0.001 | 0.027 | 5.978 | 13.310 | 0.000 | 0.022 | 1.085 | 2.416 | 0.080 | 0.514 | -1 | 1 | 0 | up_down |
| hsa-miR-503 | 1.229 | 0.875 | 9.556 | -0.293 | -9.850 | -22.235 | 0.000 | 0.006 | 8.504 | 22.168 | 0.000 | 0.007 | -1.345 | -3.507 | 0.029 | 0.251 | -1 | 1 | 0 | up_down |
| hsa-miR-532-5p | 2.325 | 1.191 | 8.929 | 3.273 | -5.655 | -7.866 | 0.002 | 0.042 | 7.171 | 11.517 | 0.001 | 0.024 | 1.515 | 2.434 | 0.078 | 0.506 | -1 | 1 | 0 | up_down |
| hsa-miR-660 | 4.074 | 3.401 | 9.128 | 3.487 | -5.640 | -10.533 | 0.001 | 0.021 | 5.391 | 11.624 | 0.001 | 0.024 | -0.250 | -0.538 | 0.622 | 1.000 | -1 | 1 | 0 | up_down |
| hsa-miR-671-5p | 3.229 | 3.674 | 11.299 | 1.237 | -10.061 | -21.632 | 0.000 | 0.006 | 7.847 | 19.482 | 0.000 | 0.010 | -2.214 | -5.497 | 0.007 | 0.090 | -1 | 1 | 0 | up_down |
| hsa-miR-372 | 3.989 | 3.544 | 12.825 | -0.719 | -13.544 | -29.118 | 0.000 | 0.005 | 9.059 | 22.486 | 0.000 | 0.007 | -4.486 | -11.136 | 0.001 | 0.015 | -1 | 1 | -1 | up_down |
| hsa-miR-151-3p | 9.767 | 8.660 | 12.269 | 5.982 | -6.287 | -8.896 | 0.001 | 0.031 | 3.055 | 4.992 | 0.010 | 0.140 | -3.232 | -5.281 | 0.008 | 0.099 | -1 | 0 | 0 | flat_down |
| hsa-miR-432 | 1.496 | 1.564 | 4.200 | 0.182 | -4.018 | -9.949 | 0.001 | 0.024 | 2.669 | 7.633 | 0.002 | 0.062 | -1.348 | -3.856 | 0.022 | 0.202 | -1 | 0 | 0 | flat_down |
| hsa-miR-598 | 2.818 | 2.693 | 3.487 | 0.490 | -2.998 | -7.355 | 0.003 | 0.049 | 0.732 | 2.074 | 0.114 | 0.661 | -2.266 | -6.420 | 0.004 | 0.061 | -1 | 0 | 0 | flat_down |
| hsa-miR-1181 | 13.482 | 13.200 | 10.560 | 2.453 | -8.107 | -18.915 | 0.000 | 0.007 | -2.781 | -7.493 | 0.002 | 0.065 | -10.888 | -29.335 | 0.000 | 0.002 | -1 | 0 | -1 | flat_down |
| hsa-miR-1224-5p | 10.620 | 11.212 | 9.178 | 1.943 | -7.236 | -14.233 | 0.000 | 0.011 | -1.737 | -3.946 | 0.020 | 0.215 | -8.973 | -20.381 | 0.000 | 0.005 | -1 | 0 | -1 | flat_down |
| hsa-miR-134 | 13.618 | 13.410 | 10.959 | 4.348 | -6.611 | -15.862 | 0.000 | 0.009 | -2.555 | -7.077 | 0.003 | 0.072 | -9.166 | -25.393 | 0.000 | 0.003 | -1 | 0 | -1 | flat_down |
| hsa-miR-148a | 13.076 | 12.825 | 13.306 | 7.328 | -5.979 | -14.128 | 0.000 | 0.011 | 0.356 | 0.971 | 0.392 | 1.000 | -5.623 | -15.342 | 0.000 | 0.007 | -1 | 0 | -1 | flat_down |
| hsa-miR-150* | 18.070 | 18.070 | 16.909 | 3.544 | -13.365 | -33.226 | 0.000 | 0.005 | -1.161 | -3.334 | 0.034 | 0.289 | -14.526 | -41.700 | 0.000 | 0.002 | -1 | 0 | -1 | flat_down |
| hsa-miR-187* | 0.613 | 0.313 | 2.884 | -2.177 | -5.061 | -11.716 | 0.001 | 0.017 | 2.420 | 6.469 | 0.004 | 0.088 | -2.641 | -7.059 | 0.003 | 0.049 | -1 | 0 | -1 | flat_down |
| hsa-miR-18b | 14.392 | 14.131 | 13.260 | 9.468 | -3.793 | -8.924 | 0.001 | 0.031 | -1.001 | -2.720 | 0.059 | 0.417 | -4.794 | -13.025 | 0.000 | 0.011 | -1 | 0 | -1 | flat_down |
| hsa-miR-193b | 7.707 | 8.135 | 5.142 | 0.084 | -5.059 | -10.984 | 0.001 | 0.019 | -2.779 | -6.968 | 0.003 | 0.073 | -7.837 | -19.651 | 0.000 | 0.005 | -1 | 0 | -1 | flat_down |
| hsa-miR-200b | 6.794 | 5.982 | 3.935 | -0.517 | -4.452 | -7.593 | 0.002 | 0.046 | -2.453 | -4.830 | 0.011 | 0.147 | -6.905 | -13.598 | 0.000 | 0.009 | -1 | 0 | -1 | flat_down |
| hsa-miR-200c | 8.748 | 9.767 | 10.166 | -0.574 | -10.740 | -16.042 | 0.000 | 0.009 | 0.908 | 1.567 | 0.199 | 0.986 | -9.832 | -16.956 | 0.000 | 0.006 | -1 | 0 | -1 | flat_down |
| hsa-miR-205 | 9.321 | 9.012 | 7.270 | -0.583 | -7.853 | -18.107 | 0.000 | 0.008 | -1.896 | -5.049 | 0.009 | 0.137 | -9.749 | -25.957 | 0.000 | 0.003 | -1 | 0 | -1 | flat_down |
| hsa-miR-210 | 11.603 | 10.700 | 6.731 | 0.645 | -6.086 | -9.789 | 0.001 | 0.025 | -4.420 | -8.209 | 0.002 | 0.052 | -10.507 | -19.513 | 0.000 | 0.005 | -1 | 0 | -1 | flat_down |
| hsa-miR-214 | 12.702 | 12.407 | 11.272 | -1.775 | -13.047 | -30.267 | 0.000 | 0.005 | -1.283 | -3.437 | 0.031 | 0.279 | -14.330 | -38.386 | 0.000 | 0.002 | -1 | 0 | -1 | flat_down |
| hsa-miR-218 | 7.531 | 6.832 | 7.531 | -0.197 | -7.728 | -14.194 | 0.000 | 0.011 | 0.349 | 0.741 | 0.504 | 1.000 | -7.379 | -15.649 | 0.000 | 0.007 | -1 | 0 | -1 | flat_down |
| hsa-miR-301a | 11.635 | 11.475 | 12.030 | 7.219 | -4.811 | -11.709 | 0.001 | 0.017 | 0.475 | 1.335 | 0.260 | 1.000 | -4.336 | -12.186 | 0.000 | 0.012 | -1 | 0 | -1 | flat_down |
| hsa-miR-302a | 10.517 | 10.862 | 11.773 | 0.867 | -10.906 | -24.719 | 0.000 | 0.005 | 1.084 | 2.836 | 0.053 | 0.390 | -9.822 | -25.707 | 0.000 | 0.003 | -1 | 0 | -1 | flat_down |
| hsa-miR-302a* | 14.928 | 14.392 | 16.676 | -1.035 | -17.712 | -36.088 | 0.000 | 0.005 | 2.016 | 4.744 | 0.011 | 0.152 | -15.695 | -36.926 | 0.000 | 0.002 | -1 | 0 | -1 | flat_down |
| hsa-miR-302b | 11.142 | 11.096 | 12.407 | 2.139 | -10.268 | -25.480 | 0.000 | 0.005 | 1.288 | 3.691 | 0.025 | 0.242 | -8.980 | -25.732 | 0.000 | 0.003 | -1 | 0 | -1 | flat_down |
| hsa-miR-302c | 13.316 | 13.076 | 15.929 | 0.449 | -15.481 | -36.720 | 0.000 | 0.005 | 2.733 | 7.486 | 0.002 | 0.065 | -12.747 | -34.914 | 0.000 | 0.002 | -1 | 0 | -1 | flat_down |
| hsa-miR-302c* | 10.700 | 10.798 | 8.252 | -1.667 | -9.919 | -24.460 | 0.000 | 0.005 | -2.497 | -7.110 | 0.003 | 0.072 | -12.416 | -35.354 | 0.000 | 0.002 | -1 | 0 | -1 | flat_down |
| hsa-miR-302d | 11.544 | 11.719 | 12.170 | 0.536 | -11.634 | -28.198 | 0.000 | 0.005 | 0.538 | 1.506 | 0.214 | 1.000 | -11.096 | -31.055 | 0.000 | 0.002 | -1 | 0 | -1 | flat_down |
| hsa-miR-34a | 12.990 | 12.872 | 12.976 | 9.321 | -3.655 | -8.979 | 0.001 | 0.031 | 0.044 | 0.126 | 0.906 | 1.000 | -3.610 | -10.242 | 0.001 | 0.019 | -1 | 0 | -1 | flat_down |
| hsa-miR-365 | 12.055 | 11.170 | 11.862 | 4.281 | -7.581 | -12.335 | 0.000 | 0.016 | 0.249 | 0.469 | 0.666 | 1.000 | -7.332 | -13.775 | 0.000 | 0.009 | -1 | 0 | -1 | flat_down |
| hsa-miR-367 | 14.013 | 13.869 | 13.986 | 0.789 | -13.197 | -32.249 | 0.000 | 0.005 | 0.045 | 0.126 | 0.906 | 1.000 | -13.152 | -37.111 | 0.000 | 0.002 | -1 | 0 | -1 | flat_down |
| hsa-miR-424 | 12.490 | 12.284 | 13.472 | 8.820 | -4.652 | -11.167 | 0.001 | 0.019 | 1.085 | 3.007 | 0.045 | 0.345 | -3.567 | -9.888 | 0.001 | 0.020 | -1 | 0 | -1 | flat_down |
| hsa-miR-455-3p | 11.096 | 10.046 | 12.990 | -0.389 | -13.380 | -19.604 | 0.000 | 0.007 | 2.420 | 4.094 | 0.018 | 0.200 | -10.960 | -18.543 | 0.000 | 0.005 | -1 | 0 | -1 | flat_down |
| hsa-miR-630 | 10.862 | 11.580 | 11.951 | 3.229 | -8.722 | -15.817 | 0.000 | 0.009 | 0.730 | 1.529 | 0.208 | 1.000 | -7.992 | -16.735 | 0.000 | 0.006 | -1 | 0 | -1 | flat_down |
| hsa-miR-7 | 7.093 | 6.501 | 7.814 | 1.744 | -6.070 | -11.948 | 0.000 | 0.017 | 1.017 | 2.311 | 0.089 | 0.547 | -5.053 | -11.484 | 0.001 | 0.014 | -1 | 0 | -1 | flat_down |
| hsa-miR-9 | 2.453 | 2.325 | 2.269 | -1.148 | -3.417 | -8.380 | 0.002 | 0.036 | -0.121 | -0.342 | 0.751 | 1.000 | -3.538 | -10.018 | 0.001 | 0.020 | -1 | 0 | -1 | flat_down |
| hsa-miR-135b | 12.170 | 11.603 | 6.382 | 0.461 | -5.921 | -11.832 | 0.001 | 0.017 | -5.504 | -12.701 | 0.000 | 0.023 | -11.425 | -26.364 | 0.000 | 0.003 | -1 | -1 | -1 | down_down |
| hsa-miR-141 | 11.982 | 12.142 | 8.364 | -1.162 | -9.525 | -23.184 | 0.000 | 0.006 | -3.698 | -10.393 | 0.001 | 0.029 | -13.224 | -37.163 | 0.000 | 0.002 | -1 | -1 | -1 | down_down |
| hsa-miR-183 | 12.243 | 11.419 | 4.045 | -0.904 | -4.949 | -8.375 | 0.002 | 0.036 | -7.786 | -15.215 | 0.000 | 0.017 | -12.735 | -24.885 | 0.000 | 0.003 | -1 | -1 | -1 | down_down |
| hsa-miR-663 | 13.544 | 13.432 | 10.431 | 6.832 | -3.598 | -8.853 | 0.001 | 0.031 | -3.057 | -8.684 | 0.001 | 0.045 | -6.656 | -18.907 | 0.000 | 0.005 | -1 | -1 | -1 | down_down |
| hsa-miR-96 | 13.200 | 12.523 | 5.051 | 0.823 | -4.229 | -7.877 | 0.002 | 0.042 | -7.810 | -16.800 | 0.000 | 0.015 | -12.039 | -25.896 | 0.000 | 0.003 | -1 | -1 | -1 | down_down |
