# Supplementary material for: Pluripotent Stem Cell‐Derived Hematopoietic Progenitors Are Unable to Downregulate Key Epithelial‐Mesenchymal Transition‐Associated miRNAs
Source: Stem Cells. 2017 Oct 27;36(1):55–64. doi: 10.1002/stem.2724 (PMC5765482; doi:10.1002/stem.2724)
Supplement: Supplementary file 3 — Supplementary Table 2 [file STEM-36-55-s003.docx]

**Supplementary table 2.**

miRNAs highlighted in **bold** were chosen for qRT-PCR analysis.

| **FLAT_DOWN** | **Pluripotency associated** | **Epithelial-mesenchymal transition** | **Haematopoiesis and angiogenesis associated** | **Cell cycle and apoptosis** | **Other** |  |
| --- | --- | --- | --- | --- | --- | --- |
| hsa-miR-151-3p |  | Regulates EMT via RhoGDIA [^1^](#_ENREF_1)^,^[^2^](#_ENREF_2) and *TWIST1* [^3^](#_ENREF_3) | Associated with switch from foetal to adult haemoglobin [^4^](#_ENREF_4)^,^[^5^](#_ENREF_5) | Targets cyclin E1[^6^](#_ENREF_6) |  |  |
| hsa-miR-432 |  |  |  | Promotes proliferation via the WNT pathway [^7^](#_ENREF_7) | Biomarker for schizophrenia [^8^](#_ENREF_8)^,^[^9^](#_ENREF_9) |  |
| hsa-miR-598 |  |  |  |  | Involved in DNA damage response pathway[^10^](#_ENREF_10) |  |
| hsa-miR-1181 |  |  |  |  | Possible role as a tumour suppressor [^11^](#_ENREF_11)^,^[^12^](#_ENREF_12) |  |
| hsa-miR-1224-5p |  |  |  |  | Possible role in inflammation [^13^](#_ENREF_13) |  |
| **hsa-miR-134** | **Regulates pluripotency via *C-MYC, NANOG, OCT4, SOX2,* and *LRH1***[**^14^**](#_ENREF_14)**^,^**[**^15^**](#_ENREF_15) | **Supresses EMT via *FOXM1***[**^16^**](#_ENREF_16)**^,^**[**^17^**](#_ENREF_17) | **Enhances erythroid differentiation** [**^18^**](#_ENREF_18) |  | **Promotes ectodermal commitment in mESCs** [**^15^**](#_ENREF_15) |  |
| **hsa-miR-148a** |  |  | **Role in innate immunity via NF-KB pathway** [**^19^**](#_ENREF_19) | **Targets p53, mTOR** [**^20^**](#_ENREF_20)**^,^**[**^21^**](#_ENREF_21) | **miR-148a is a tumour suppressor; promotes adipogenesis and osteoclastogenesis** [**^22^**](#_ENREF_22)**, suppresses mesenchymal lineage via *EPAS1*, and targets *DNMT1***[***^23^***](#_ENREF_23) |  |
| **hsa-miR-18b** |  | **Modulates genes controlling cell migration** [**^24^**](#_ENREF_24) | **Down-regulated during erythroid differentiation, targets** **hematopoetic genes *STAT3, HIF-1α, VEGF*** [**^25^**](#_ENREF_25) | **Targets the MDM2-p53 pathway** [**^26^**](#_ENREF_26) | **Promotes epithelial differentiation** [**^27^**](#_ENREF_27) |  |
| hsa-miR-193b |  | Suppresses metastasis via targeting uPA, and *NF1* [^28^](#_ENREF_28)^,^[^29^](#_ENREF_29) | Targets hematopoietic transcription factors *C-KIT* [^30^](#_ENREF_30) and *c-MYB* [^31^](#_ENREF_31) | Targets Cyclin D1 [^32^](#_ENREF_32)^,^[^33^](#_ENREF_33) |  |  |
| **hsa-miR-200b** |  | **Targets critical EMT regulators *ZEB1, ZEB2, BMI1, RND3, SNAIL* and *ETS1*** [**^34-36^**](#_ENREF_34) | **Regulates angiogenesis via *ZEB1*** [**^36^**](#_ENREF_36)**, and targets TLR4 and NF-KB immune system pathways** [**^37^**](#_ENREF_37) |  |  |  |
| **hsa-miR-200c** | **Enhances reprogramming of cells to pluripotency** [**^38^**](#_ENREF_38) | **Inhibits EMT via *BIN1, ZEB1, RND3, LHFP* and *TGFb*** [**^37^**](#_ENREF_37)**^,^**[**^39^**](#_ENREF_39) | **Targets *VEGF*** [**^40^**](#_ENREF_40)**, *ETS1*** [**^41^**](#_ENREF_41) **and *cMYB*** [**^42^**](#_ENREF_42) |  |  |  |
| **hsa-miR-205** |  | **Targets *ZEB1*and *ZEB2*** [**^34^**](#_ENREF_34) |  | ***Targets E2F1 and PTEN*.** | **Plays a complex role in tumour formation** [**^43^**](#_ENREF_43) |  |
| hsa-miR-210 |  |  |  |  | Key miRNA in the hypoxia response [^2^](#_ENREF_2)^,^[^44^](#_ENREF_44) |  |
| hsa-miR-214 |  |  | Targets *VEGF* [^45^](#_ENREF_45) | Targets p53 [^46^](#_ENREF_46) | Biomarker for cancer [^47^](#_ENREF_47)^,^[^48^](#_ENREF_48) and promotes osteoblastogenesis [^49^](#_ENREF_49)^,^[^50^](#_ENREF_50) |  |
| hsa-miR-218 |  |  | Regulates vascular patterning via *SLIT-ROBO* signalling [^51^](#_ENREF_51) |  | Tumour suppressor [^28^](#_ENREF_28) |  |
| hsa-miR-301a |  | Targets E-cadherin [^52^](#_ENREF_52) | Regulates NF-κB signalling [^53^](#_ENREF_53)^,^[^54^](#_ENREF_54) |  |  |  |
| **hsa-miR-302a, b, c, d, hsa-miR-367** | **Maintians and promotes pluripotency** [**^38^**](#_ENREF_38)**^,^**[**^55^**](#_ENREF_55)**^,^**[**^56^**](#_ENREF_56) |  |  | **Targets cyclin E-CDK2 and cyclin D-CDK4/6 pathways** [**^57^**](#_ENREF_57) |  |  |
| **hsa-miR-34a** |  | **Targets *MYC*** [**^58^**](#_ENREF_58) | **Targets CEBPA, a transcripton factor which promotes myeloid differentiation** [**^59^**](#_ENREF_59)**,**  **also targets NOTCH1 and FOXP1, regulating B cell differentiation** [**^60^**](#_ENREF_60) |  | **Key role as a tumour suppressor** [**^28^**](#_ENREF_28)**^,^**[**^61^**](#_ENREF_61) |  |
| hsa-miR-365 |  |  |  |  | Promotes adipocyte differentiation [^62^](#_ENREF_62) |  |
| **hsa-miR-424** |  |  | **Targets haematopoietic genes *PU.1*** [**^63^**](#_ENREF_63)**, *CEBPA***[***^64^***](#_ENREF_64)***, VEGF, VEGFR2, FGF2, FGF1* and *FGFR2***[***^65^***](#_ENREF_65) | **Targets CyclinE1, CDC25A and MEK**[**^66^**](#_ENREF_66) | **Controls myeloid and monocyte differentiation** [**^63^**](#_ENREF_63) **and inhibits angiogenesis** [**^67^**](#_ENREF_67)**.** |  |
| hsa-miR-455-3p |  | Targets *ZEB1* [^68^](#_ENREF_68) |  |  | Regulates chondrogenesis [^69^](#_ENREF_69) and adipogenesis [^70^](#_ENREF_70) |  |
| hsa-miR-630 |  | Inhibits EMT via SLUG-TGFB pathway [^71-73^](#_ENREF_71) |  |  |  |  |
| hsa-miR-7 |  | Regulates inflammation and innate immunity [^74^](#_ENREF_74) |  |  | Role in organogenesis, particularly the brain [^75^](#_ENREF_75) |  |
| **hsa-miR-9** | **Targets LIN28** [**^76^**](#_ENREF_76) | **Targets *E-Cadherin*** [**^77^**](#_ENREF_77) | **Regulates myeloid differentiation via *FOX01* and *FOX03*** [**^78^**](#_ENREF_78) | **Targets Cyclin D1 and *ETS1*** [**^79^**](#_ENREF_79) | **Promotes pluripotency** [**^76^**](#_ENREF_76) **Also promotes terminal differentiation and apoptosis in haematopoetic cells** [**^78^**](#_ENREF_78) |  |
|  |  |  |  |  |  |  |

1 Ding, J. *et al.* Gain of miR-151 on chromosome 8q24.3 facilitates tumour cell migration and spreading through downregulating RhoGDIA. *Nature cell biology* **12**, 390-399, doi:10.1038/ncb2039 (2010).

2 Luedde, T. MicroRNA-151 and its hosting gene FAK (focal adhesion kinase) regulate tumor cell migration and spreading of hepatocellular carcinoma. *Hepatology (Baltimore, Md.)* **52**, 1164-1166, doi:10.1002/hep.23854 (2010).

3 Nairismagi, M. L., Fuchtbauer, A., Labouriau, R., Bramsen, J. B. & Fuchtbauer, E. M. The proto-oncogene TWIST1 is regulated by microRNAs. *PloS one* **8**, e66070, doi:10.1371/journal.pone.0066070 (2013).

4 Pule, G. D., Mowla, S., Novitzky, N. & Wonkam, A. Hydroxyurea down-regulates BCL11A, KLF-1 and MYB through miRNA-mediated actions to induce gamma-globin expression: implications for new therapeutic approaches of sickle cell disease. *Clinical and translational medicine* **5**, 15, doi:10.1186/s40169-016-0092-7 (2016).

5 Walker, A. L. *et al.* Epigenetic and molecular profiles of erythroid cells after hydroxyurea treatment in sickle cell anemia. *Blood* **118**, 5664-5670, doi:10.1182/blood-2011-07-368746 (2011).

6 Liu, Y. *et al.* A miR-151 binding site polymorphism in the 3'-untranslated region of the cyclin E1 gene associated with nasopharyngeal carcinoma. *Biochemical and biophysical research communications* **432**, 660-665, doi:10.1016/j.bbrc.2013.02.024 (2013).

7 Jiang, N. *et al.* Downregulation of miR-432 activates Wnt/beta-catenin signaling and promotes human hepatocellular carcinoma proliferation. *Oncotarget* **6**, 7866-7879, doi:10.18632/oncotarget.3492 (2015).

8 Lai, C. Y. *et al.* MicroRNA expression aberration as potential peripheral blood biomarkers for schizophrenia. *PloS one* **6**, e21635, doi:10.1371/journal.pone.0021635 (2011).

9 Yu, H. C. *et al.* Alterations of miR-132 are novel diagnostic biomarkers in peripheral blood of schizophrenia patients. *Progress in neuro-psychopharmacology & biological psychiatry* **63**, 23-29, doi:10.1016/j.pnpbp.2015.05.007 (2015).

10 Girardi, C. *et al.* Analysis of miRNA and mRNA expression profiles highlights alterations in ionizing radiation response of human lymphocytes under modeled microgravity. *PloS one* **7**, e31293, doi:10.1371/journal.pone.0031293 (2012).

11 Zhang, H. Y., Li, J. H., Li, G. & Wang, S. R. Activation of ARK5/miR-1181/HOXA10 axis promotes epithelial-mesenchymal transition in ovarian cancer. *Oncology reports* **34**, 1193-1202, doi:10.3892/or.2015.4113 (2015).

12 Jiang, J. *et al.* MiR-1181 inhibits stem cell-like phenotypes and suppresses SOX2 and STAT3 in human pancreatic cancer. *Cancer letters* **356**, 962-970, doi:10.1016/j.canlet.2014.11.007 (2015).

13 Niu, Y. *et al.* Lipopolysaccharide-induced miR-1224 negatively regulates tumour necrosis factor-alpha gene expression by modulating Sp1. *Immunology* **133**, 8-20, doi:10.1111/j.1365-2567.2010.03374.x (2011).

14 Zhang, L. *et al.* MiR-134-Mbd3 axis regulates the induction of pluripotency. *Journal of cellular and molecular medicine* **20**, 1150-1158, doi:10.1111/jcmm.12805 (2016).

15 Tay, Y. M. *et al.* MicroRNA-134 modulates the differentiation of mouse embryonic stem cells, where it causes post-transcriptional attenuation of Nanog and LRH1. *Stem cells (Dayton, Ohio)* **26**, 17-29, doi:10.1634/stemcells.2007-0295 (2008).

16 Liu, Y. *et al.* miR-134 functions as a tumor suppressor in cell proliferation and epithelial-to-mesenchymal Transition by targeting KRAS in renal cell carcinoma cells. *DNA and cell biology* **34**, 429-436, doi:10.1089/dna.2014.2629 (2015).

17 Li, J. *et al.* miR-134 inhibits epithelial to mesenchymal transition by targeting FOXM1 in non-small cell lung cancer cells. *FEBS letters* **586**, 3761-3765, doi:10.1016/j.febslet.2012.09.016 (2012).

18 Chae, H. D., Lee, M. R. & Broxmeyer, H. E. 5-Aminoimidazole-4-carboxyamide ribonucleoside induces G(1)/S arrest and Nanog downregulation via p53 and enhances erythroid differentiation. *Stem cells (Dayton, Ohio)* **30**, 140-149, doi:10.1002/stem.778 (2012).

19 Patel, V. *et al.* The stretch responsive microRNA miR-148a-3p is a novel repressor of IKBKB, NF-kappaB signaling, and inflammatory gene expression in human aortic valve cells. *FASEB journal : official publication of the Federation of American Societies for Experimental Biology* **29**, 1859-1868, doi:10.1096/fj.14-257808 (2015).

20 Xu, X. *et al.* Hepatitis B virus X protein represses miRNA-148a to enhance tumorigenesis. *The Journal of clinical investigation* **123**, 630-645, doi:10.1172/jci64265 (2013).

21 Gao, J. *et al.* MicroRNA expression during osteogenic differentiation of human multipotent mesenchymal stromal cells from bone marrow. *Journal of cellular biochemistry* **112**, 1844-1856, doi:10.1002/jcb.23106 (2011).

22 Shi, C. *et al.* Adipogenic miRNA and meta-signature miRNAs involved in human adipocyte differentiation and obesity. *Oncotarget*, doi:10.18632/oncotarget.8518 (2016).

23 Hsiao, K. Y. *et al.* Coordination of AUF1 and miR-148a destabilizes DNA methyltransferase 1 mRNA under hypoxia in endometriosis. *Molecular human reproduction* **21**, 894-904, doi:10.1093/molehr/gav054 (2015).

24 Fonseca-Sanchez, M. A. *et al.* microRNA-18b is upregulated in breast cancer and modulates genes involved in cell migration. *Oncology reports* **30**, 2399-2410, doi:10.3892/or.2013.2691 (2013).

25 Yang, G. H. *et al.* MicroRNAs are involved in erythroid differentiation control. *Journal of cellular biochemistry* **107**, 548-556, doi:10.1002/jcb.22156 (2009).

26 Jazirehi, A. R., Torres-Collado, A. X. & Nazarian, R. Role of miR-18b/MDM2/p53 circuitry in melanoma progression. *Epigenomics* **5**, 254 (2013).

27 Kushwaha, R., Thodima, V., Tomishima, M. J., Bosl, G. J. & Chaganti, R. S. miR-18b and miR-518b Target FOXN1 during epithelial lineage differentiation in pluripotent cells. *Stem cells and development* **23**, 1149-1156, doi:10.1089/scd.2013.0262 (2014).

28 Jamali, Z. *et al.* MicroRNAs as prognostic molecular signatures in human head and neck squamous cell carcinoma: a systematic review and meta-analysis. *Oral oncology* **51**, 321-331, doi:10.1016/j.oraloncology.2015.01.008 (2015).

29 Li, J. *et al.* miR-193b directly targets STMN1 and uPA genes and suppresses tumor growth and metastasis in pancreatic cancer. *Molecular medicine reports* **10**, 2613-2620, doi:10.3892/mmr.2014.2558 (2014).

30 Gao, X. N. *et al.* MicroRNA-193b regulates c-Kit proto-oncogene and represses cell proliferation in acute myeloid leukemia. *Leukemia research* **35**, 1226-1232, doi:10.1016/j.leukres.2011.06.010 (2011).

31 Mets, E. *et al.* MicroRNA-193b-3p acts as a tumor suppressor by targeting the MYB oncogene in T-cell acute lymphoblastic leukemia. *Leukemia* **29**, 798-806, doi:10.1038/leu.2014.276 (2015).

32 Wang, L. *et al.* MicroRNA-193b inhibits the proliferation, migration and invasion of gastric cancer cells via targeting cyclin D1. *Acta histochemica*, doi:10.1016/j.acthis.2016.02.001 (2016).

33 Chen, J. *et al.* MicroRNA-193b represses cell proliferation and regulates cyclin D1 in melanoma. *The American journal of pathology* **176**, 2520-2529, doi:10.2353/ajpath.2010.091061 (2010).

34 Gregory, P. A. *et al.* The miR-200 family and miR-205 regulate epithelial to mesenchymal transition by targeting ZEB1 and SIP1. *Nature cell biology* **10**, 593-601, doi:10.1038/ncb1722 (2008).

35 Hur, K. *et al.* MicroRNA-200c modulates epithelial-to-mesenchymal transition (EMT) in human colorectal cancer metastasis. *Gut* **62**, 1315-1326, doi:10.1136/gutjnl-2011-301846 (2013).

36 Luo, Z. *et al.* MicroRNA-200C and -150 play an important role in endothelial cell differentiation and vasculogenesis by targeting transcription repressor ZEB1. *Stem cells (Dayton, Ohio)* **31**, 1749-1762, doi:10.1002/stem.1448 (2013).

37 Wendlandt, E. B., Graff, J. W., Gioannini, T. L., McCaffrey, A. P. & Wilson, M. E. The role of microRNAs miR-200b and miR-200c in TLR4 signaling and NF-kappaB activation. *Innate immunity* **18**, 846-855, doi:10.1177/1753425912443903 (2012).

38 Miyoshi, N. *et al.* Reprogramming of mouse and human cells to pluripotency using mature microRNAs. *Cell stem cell* **8**, 633-638, doi:10.1016/j.stem.2011.05.001 (2011).

39 Gregory, P. A. *et al.* An autocrine TGF-beta/ZEB/miR-200 signaling network regulates establishment and maintenance of epithelial-mesenchymal transition. *Molecular biology of the cell* **22**, 1686-1698, doi:10.1091/mbc.E11-02-0103 (2011).

40 Choi, Y. C., Yoon, S., Jeong, Y., Yoon, J. & Baek, K. Regulation of vascular endothelial growth factor signaling by miR-200b. *Molecules and cells* **32**, 77-82, doi:10.1007/s10059-011-1042-2 (2011).

41 Chan, Y. C., Khanna, S., Roy, S. & Sen, C. K. miR-200b targets Ets-1 and is down-regulated by hypoxia to induce angiogenic response of endothelial cells. *The Journal of biological chemistry* **286**, 2047-2056, doi:10.1074/jbc.M110.158790 (2011).

42 Cesi, V. *et al.* TGFbeta-induced c-Myb affects the expression of EMT-associated genes and promotes invasion of ER+ breast cancer cells. *Cell cycle (Georgetown, Tex.)* **10**, 4149-4161, doi:10.4161/cc.10.23.18346 (2011).

43 Tian, L. *et al.* MicroRNA-205 suppresses proliferation and promotes apoptosis in laryngeal squamous cell carcinoma. *Medical oncology (Northwood, London, England)* **31**, 785, doi:10.1007/s12032-013-0785-3 (2014).

44 McDermott, S. P., Eppert, K., Lechman, E. R., Doedens, M. & Dick, J. E. Comparison of human cord blood engraftment between immunocompromised mouse strains. *Blood* **116**, 193-200, doi:10.1182/blood-2010-02-271841 (2010).

45 Jin, Y. *et al.* MiR-214 regulates the pathogenesis of patients with coronary artery disease by targeting VEGF. *Molecular and cellular biochemistry* **402**, 111-122, doi:10.1007/s11010-014-2319-5 (2015).

46 Wang, F., Lv, P., Liu, X., Zhu, M. & Qiu, X. microRNA-214 enhances the invasion ability of breast cancer cells by targeting p53. *International journal of molecular medicine* **35**, 1395-1402, doi:10.3892/ijmm.2015.2123 (2015).

47 Nagata, M., Muto, S. & Horie, S. Molecular Biomarkers in Bladder Cancer: Novel Potential Indicators of Prognosis and Treatment Outcomes. **2016**, 8205836, doi:10.1155/2016/8205836 (2016).

48 Sharma, T., Hamilton, R. & Mandal, C. C. miR-214: a potential biomarker and therapeutic for different cancers. *Future oncology (London, England)* **11**, 349-363, doi:10.2217/fon.14.193 (2015).

49 Yang, L. *et al.* MiR-214 Attenuates Osteogenic Differentiation of Mesenchymal Stem Cells via Targeting FGFR1. *Cellular physiology and biochemistry : international journal of experimental cellular physiology, biochemistry, and pharmacology* **38**, 809-820, doi:10.1159/000443036 (2016).

50 Zhao, C. *et al.* miR-214 promotes osteoclastogenesis by targeting Pten/PI3k/Akt pathway. *RNA biology* **12**, 343-353, doi:10.1080/15476286.2015.1017205 (2015).

51 Kong, Y. *et al.* Slit-miR-218-Robo axis regulates retinal neovascularization. *International journal of molecular medicine* **37**, 1139-1145, doi:10.3892/ijmm.2016.2511 (2016).

52 Nam, R. K. *et al.* MiR-301a regulates E-cadherin expression and is predictive of prostate cancer recurrence. *The Prostate* **76**, 869-884, doi:10.1002/pros.23177 (2016).

53 Ma, X., Becker Buscaglia, L. E., Barker, J. R. & Li, Y. MicroRNAs in NF-kappaB signaling. *Journal of molecular cell biology* **3**, 159-166, doi:10.1093/jmcb/mjr007 (2011).

54 Yang, Y. & Wang, J. K. The functional analysis of MicroRNAs involved in NF-kappaB signaling. *European review for medical and pharmacological sciences* **20**, 1764-1774 (2016).

55 Barroso-del Jesus, A., Lucena-Aguilar, G. & Menendez, P. The miR-302-367 cluster as a potential stemness regulator in ESCs. *Cell cycle (Georgetown, Tex.)* **8**, 394-398, doi:10.4161/cc.8.3.7554 (2009).

56 Lin, S. L. *et al.* Regulation of somatic cell reprogramming through inducible mir-302 expression. *Nucleic acids research* **39**, 1054-1065, doi:10.1093/nar/gkq850 (2011).

57 Nakano, T., Kodama, H. & Honjo, T. Generation of lymphohematopoietic cells from embryonic stem cells in culture. *Science (New York, N.Y.)* **265**, 1098-1101 (1994).

58 Sun, M., Chen, H., Liu, J., Tong, C. & Meng, T. MicroRNA-34a inhibits human trophoblast cell invasion by targeting MYC. *BMC cell biology* **16**, 21, doi:10.1186/s12860-015-0068-2 (2015).

59 Kajikhina, K., Tsuneto, M. & Melchers, F. Environments of hematopoiesis and B-lymphopoiesis in foetal liver. *Clinical and experimental rheumatology* **33**, S91-93 (2015).

60 Schaefer, A. & Hordijk, P. L. Cell-stiffness-induced mechanosignaling - a key driver of leukocyte transendothelial migration. *Journal of cell science* **128**, 2221-2230, doi:10.1242/jcs.163055 (2015).

61 https://[www.thermofisher.com/uk/en/home/brands/thermo-scientific/molecular-biology/molecular-biology-learning-center/molecular-biology-resource-library/thermo-scientific-web-tools/multiple-primer-analyzer.html](http://www.thermofisher.com/uk/en/home/brands/thermo-scientific/molecular-biology/molecular-biology-learning-center/molecular-biology-resource-library/thermo-scientific-web-tools/multiple-primer-analyzer.html).

62 Vienberg, S., Geiger, J., Madsen, S. & Dalgaard, L. T. MicroRNAs in Metabolism. *Acta physiologica (Oxford, England)*, doi:10.1111/apha.12681 (2016).

63 Rosa, A. *et al.* The interplay between the master transcription factor PU.1 and miR-424 regulates human monocyte/macrophage differentiation. *Proceedings of the National Academy of Sciences of the United States of America* **104**, 19849-19854, doi:10.1073/pnas.0706963104 (2007).

64 Shen, X. *et al.* MiR-424 regulates monocytic differentiation of human leukemia U937 cells by directly targeting CDX2. *Biotechnology letters* **35**, 1799-1806, doi:10.1007/s10529-013-1264-9 (2013).

65 Chamorro-Jorganes, A. *et al.* MicroRNA-16 and microRNA-424 regulate cell-autonomous angiogenic functions in endothelial cells via targeting vascular endothelial growth factor receptor-2 and fibroblast growth factor receptor-1. *Arteriosclerosis, thrombosis, and vascular biology* **31**, 2595-2606, doi:10.1161/atvbaha.111.236521 (2011).

66 Nakashima, T. *et al.* Down-regulation of mir-424 contributes to the abnormal angiogenesis via MEK1 and cyclin E1 in senile hemangioma: its implications to therapy. *PloS one* **5**, e14334, doi:10.1371/journal.pone.0014334 (2010).

67 Ghosh, G. *et al.* Hypoxia-induced microRNA-424 expression in human endothelial cells regulates HIF-alpha isoforms and promotes angiogenesis. *The Journal of clinical investigation* **120**, 4141-4154, doi:10.1172/jci42980 (2010).

68 Li, Y. J., Ping, C., Tang, J. & Zhang, W. MicroRNA-455 suppresses non-small cell lung cancer through targeting ZEB1. *Cell biology international* **40**, 621-628, doi:10.1002/cbin.10584 (2016).

69 Zhang, Z. *et al.* MiR-455-3p regulates early chondrogenic differentiation via inhibiting Runx2. *FEBS letters* **589**, 3671-3678, doi:10.1016/j.febslet.2015.09.032 (2015).

70 Zhang, H. *et al.* MicroRNA-455 regulates brown adipogenesis via a novel HIF1an-AMPK-PGC1alpha signaling network. *EMBO reports* **16**, 1378-1393, doi:10.15252/embr.201540837 (2015).

71 Chen, W. X. *et al.* MicroRNA-630 suppresses tumor metastasis through the TGF-beta- miR-630-Slug signaling pathway and correlates inversely with poor prognosis in hepatocellular carcinoma. *Oncotarget*, doi:10.18632/oncotarget.8047 (2016).

72 Li, X., Lin, Y., Yang, X., Wu, X. & He, X. Long noncoding RNA H19 regulates EZH2 expression by interacting with miR-630 and promotes cell invasion in nasopharyngeal carcinoma. *Biochemical and biophysical research communications* **473**, 913-919, doi:10.1016/j.bbrc.2016.03.150 (2016).

73 Sun, Y. *et al.* MiR-630 Inhibits Endothelial-Mesenchymal Transition by Targeting Slug in Traumatic Heterotopic Ossification. *Scientific reports* **6**, 22729, doi:10.1038/srep22729 (2016).

74 Giles, K. M. *et al.* microRNA-7-5p inhibits melanoma cell proliferation and metastasis by suppressing RelA/NF-kappaB. *Oncotarget*, doi:10.18632/oncotarget.9421 (2016).

75 Horsham, J. L. *et al.* MicroRNA-7: A miRNA with expanding roles in development and disease. *The international journal of biochemistry & cell biology* **69**, 215-224, doi:10.1016/j.biocel.2015.11.001 (2015).

76 Zhong, X. *et al.* Identification of microRNAs regulating reprogramming factor LIN28 in embryonic stem cells and cancer cells. *The Journal of biological chemistry* **285**, 41961-41971, doi:10.1074/jbc.M110.169607 (2010).

77 Liu, M. *et al.* c-Myc suppressed E-cadherin through miR-9 at the post-transcriptional level. *Cell biology international* **37**, 197-202, doi:10.1002/cbin.10039 (2013).

78 Senyuk, V. *et al.* Critical role of miR-9 in myelopoiesis and EVI1-induced leukemogenesis. *Proceedings of the National Academy of Sciences of the United States of America* **110**, 5594-5599, doi:10.1073/pnas.1302645110 (2013).

79 Zheng, L. *et al.* microRNA-9 suppresses the proliferation, invasion and metastasis of gastric cancer cells through targeting cyclin D1 and Ets1. *PloS one* **8**, e55719, doi:10.1371/journal.pone.0055719 (2013).
