# Supplementary material for: Pluripotent Stem Cell‐Derived Hematopoietic Progenitors Are Unable to Downregulate Key Epithelial‐Mesenchymal Transition‐Associated miRNAs
Source: Stem Cells. 2017 Oct 27;36(1):55–64. doi: 10.1002/stem.2724 (PMC5765482; doi:10.1002/stem.2724)
Supplement: Supplementary file 4 — Supplementary Table 3 [file STEM-36-55-s004.docx]

| **Supplementary table 3: Primers for qPCR** | | |
| --- | --- | --- |
| Gene | Forward primer | Reverse primer |
| SCL | AGCCGGATGCCTTCCCTAT | CCGCACAACTTTGGTGTGG |
| GATA2 | GGGGACCCTGTCTGCAACGC | GGCAGCTGCACTGAAGGGGG |
| cMYB | GACAGCAGGTGCTACCAACA | GCTGCATGTGTGGTTCTGTG |
| RORA | GCACCGCGGCTTAAATGATGT | CTTCTCCTGAAAAAGCCCTTGC |
| RUNX1 | CAATGGATCCCAGGTATTGG | CACTGCCTTTAACCCTCAGC |
| ZEB1 | GCGGAAGACAGAAAATGGAA | GATTCCACACTCATGAGGTC |
| ZEB2 | AATGAAGCAGCCGATCATGG | CAGAACCTGTGTCCACTAC |
